# Supplementary material for: HDAC activity is dispensable for repression of cell-cycle genes by DREAM and E2F:RB complexes
Source: Nat Commun. 2024 May 24;15:4450. doi: 10.1038/s41467-024-48724-0 (PMC11126580; doi:10.1038/s41467-024-48724-0)
Supplement: Supplementary file 1 — Supplementary Information [file 41467_2024_48724_MOESM1_ESM.pdf]

Supplementary Fig. 1

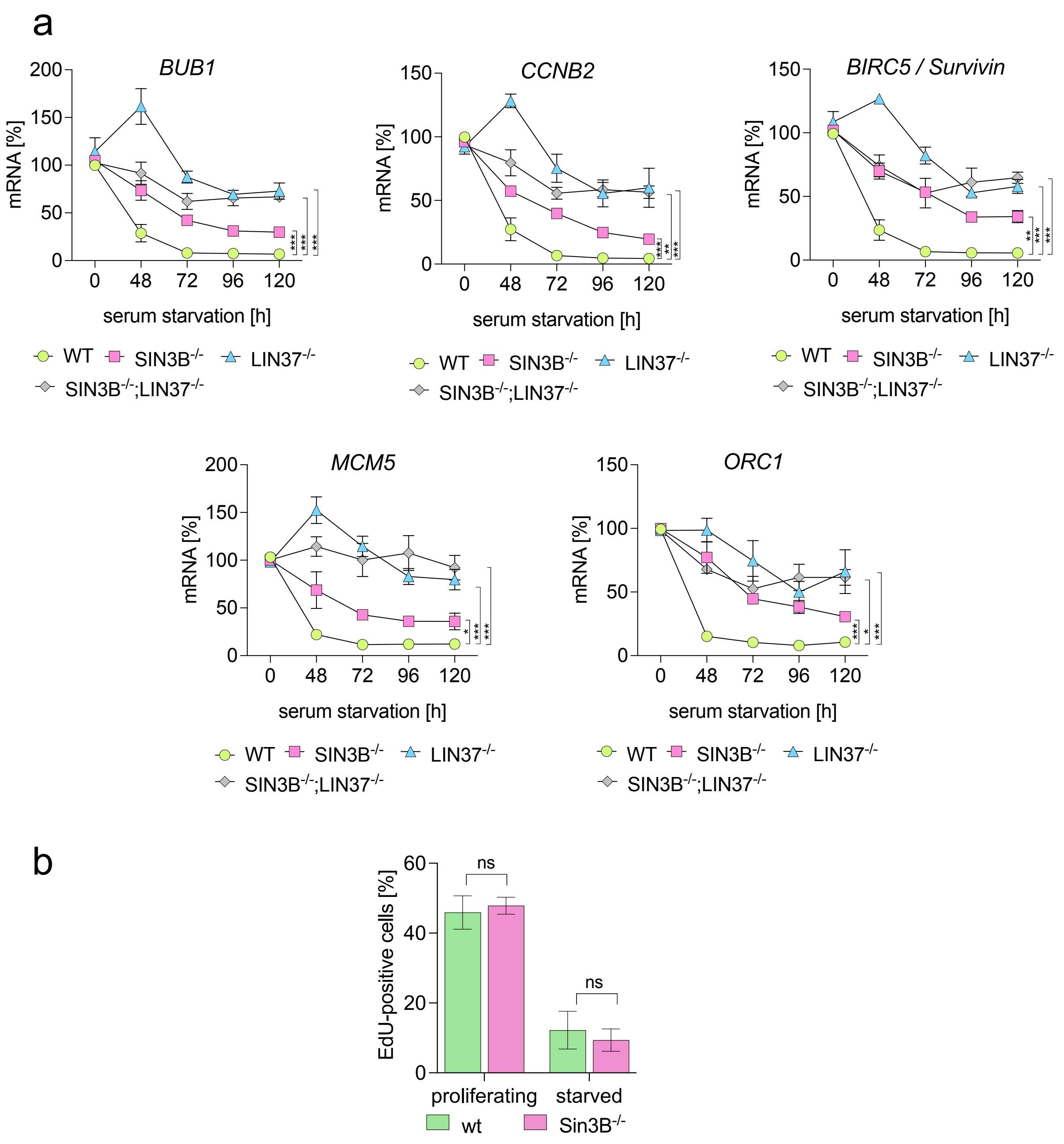

Supplementary Fig. 1: (a) mRNA expression of G2/M (BUB1, CCNB2, BIRC5) and G1/S (MCM5, ORC1) cell-cycle genes was analyzed by RT-qPCR in wild-type and knockout T98G lines arrested by serum-starvation. Two independent SIN3B$^{-/-}$, LIN37$^{-/-}$, and SIN3B$^{-/-}$;LIN37$^{-/-}$ clones were compared with two wild-type (WT) clones measured with two technical replicates each. The 0 hour time points of one WT, SIN3B$^{-/-}$, LIN37$^{-/-}$, and SIN3B$^{-/-}$;LIN37$^{-/-}$ clone were set to 100%, and all other data points for each genotype were calculated relative to the 100% value. Averages ±SEM are shown. Significances of differences in gene expression at the 120 h time point were analyzed with the two-tailed Student's T-Test. (b) EdU incorporation was analyzed in wild-type and Sin3B$^{-/-}$ C2C12 lines. Averages ±SD of 3 clones each after 48 h of serum deprivation are shown. Source data are provided as a Source Data file.

Supplementary Fig. 2

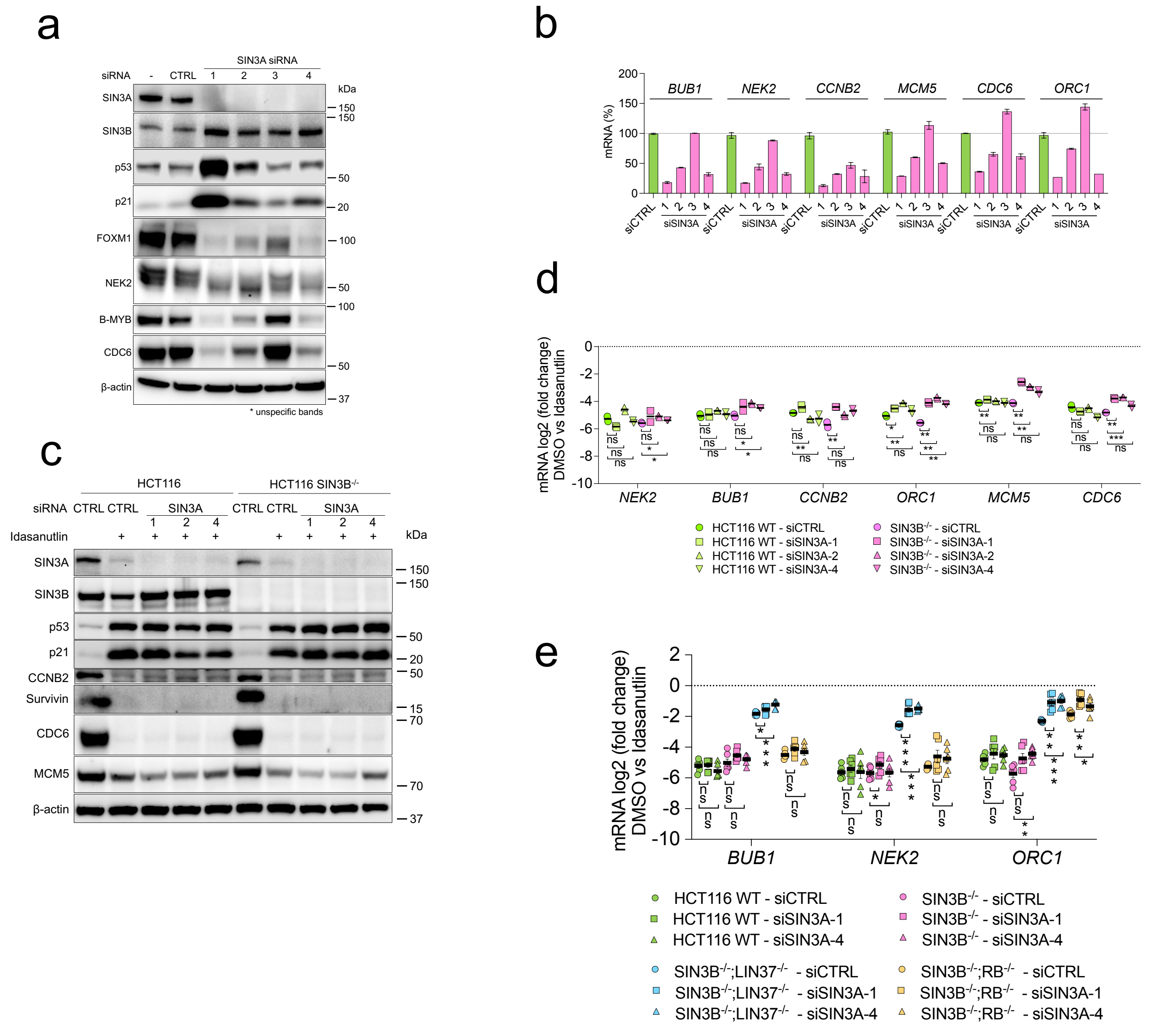

Supplementary Fig. 2: Combined loss of SIN3A and SIN3B increases cell-cycle gene mRNA expression in arrested HCT116 cells, while loss of SIN3A alone has only minor effects. (A) HCT116 cells were transfected with four SIN3A siRNAs or a non-targeting control siRNA (CTRL), and protein expression was analyzed after 48 h by Western blotting. Mean values of a representative experiment (two experiments total) with two technical replicates (± SD) are shown. (B) mRNA expression of representative cell-cycle genes in cells treated as described in (A) was analyzed by RT-qPCR (one representative experiment, two experiments total, mean values ± SD). (C) HCT116 wild-type and SIN3B$^{-/-}$ cells were transfected for 48 h with three SIN3A-targeting siRNAs or a non-targeting control siRNA, and 5 µM Idasanutlin was applied for the final 24 h. Protein levels were analyzed by Western blotting, and (D) mRNA expression of cell-cycle genes was measured by RT-qPCR. One representative experiment out of two replicates with two technical replicates is shown. (E) mRNA expression of cell-cycle genes that were not identified as significantly upregulated by RNA-seq in arrested HCT116 cells depleted of SIN3A and SIN3B was analyzed by RT-qPCR. Averages of three biological and two technical replicates are given (mean values ± SEM). Significances were calculated with the two-tailed Student's T-Test (ns – not significant, * p ≤.05, ** p ≤ .01, *** p ≤ .001). Source data are provided as a Source Data file.

Supplementary Figure 3

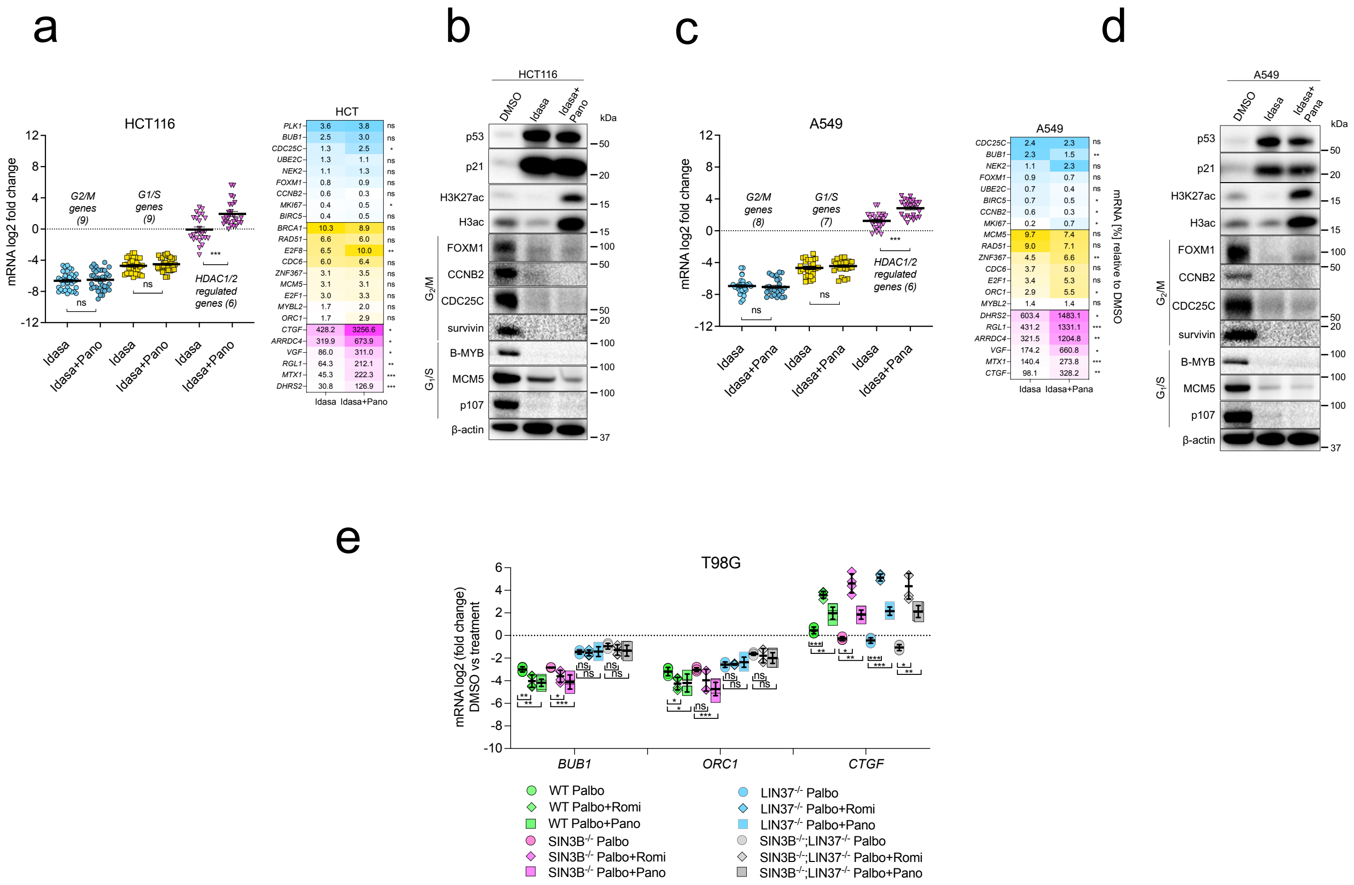

Supplementary Fig. 3: HDACi by the pan-HDAC inhibitor Panobinostat does not lead to a general upregulation of DREAM and E2F:RB target genes in arrested cells. (A, B) HCT116 and (C, D) A549 were treated with 5 µM Idasanutlin for 48 h and 20 nM Panobinostat for the final 24 h. (A, C) mRNA expression was evaluated by RT-qPCR and compared to DMSO-treated cells for each respective line. Expression data are shown as gene-set clusters (left) and individual genes (right). The datasets contain two biological replicates with two technical each presented as mean values ± SEM. (B, D) Protein expression and histone acetylation were evaluated via Western blotting with the indicated antibodies. A biological replicate of each Western blot experiment produced similar results. (E) The indicated T98G wild-type and knockout lines (two clones each) were treated with 10 µM Palbociclib for 48 h and with 4 nM Romidepsin or 20 nM Panobinostat for the final 24 h when indicated. mRNA levels were evaluated by RT-qPCR (two technical replicates) and compared to DMSO-treated cells. Mean values ± SD are shown. Significances in (A), (C), and (E) were calculated with the two-tailed Students T-Test (ns – not significant, * p ≤.05, ** p ≤ .01, *** p ≤ .001). Source data are provided as a Source Data file.

Supplementary Fig. 4

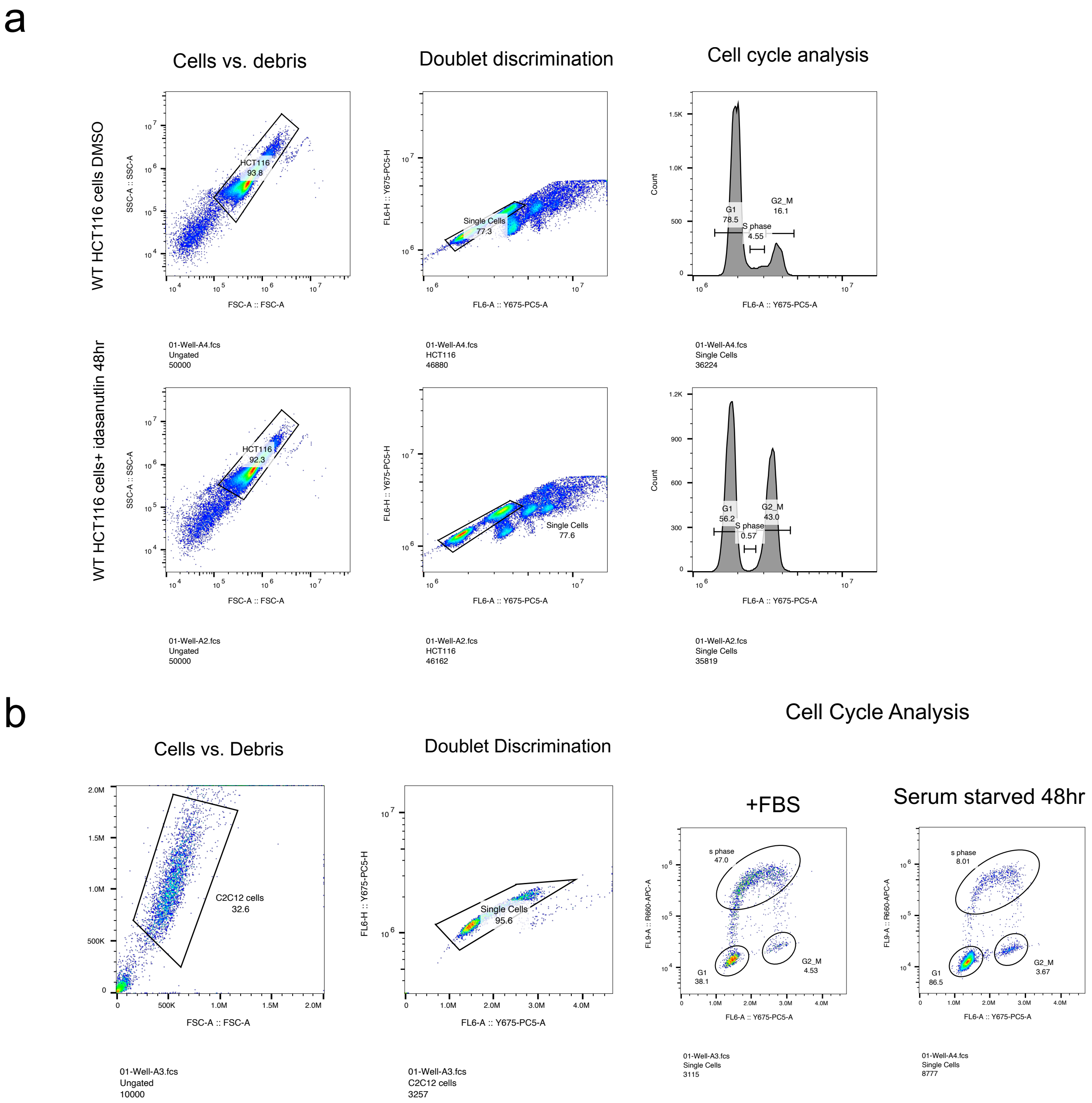

Supplementary Fig. 4: Gating and analysis strategies for flow cytometry data. a) Strategy for collecting cell cycle data after DNA staining with propidium iodide (PI). We analyzed these data with manually set gates instead of using an algorithm, because automatized methods usually do not perform well with arrested cell populations that contain an extremely low number of S phase cells. All data shown in Fig. 2e, 2f, 4h, 5c, 5e, 6d, 6e were generated and analyzed with this approach. b) Strategy for analyzing S phase cells after EdU incorporation. Data shown in Supplementary Fig. 1b were generated with this approach.

Uncropped blots - Supplementary Fig. 2a

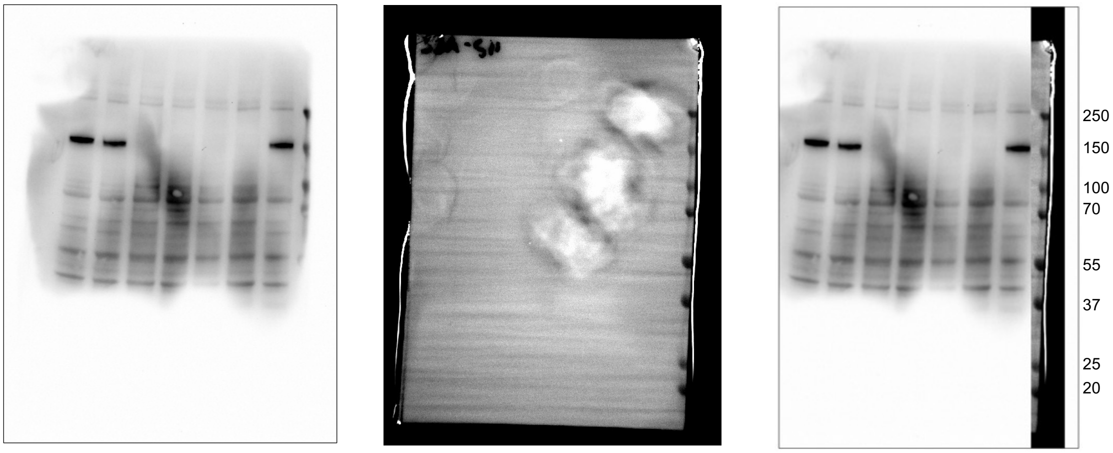

SIN3A

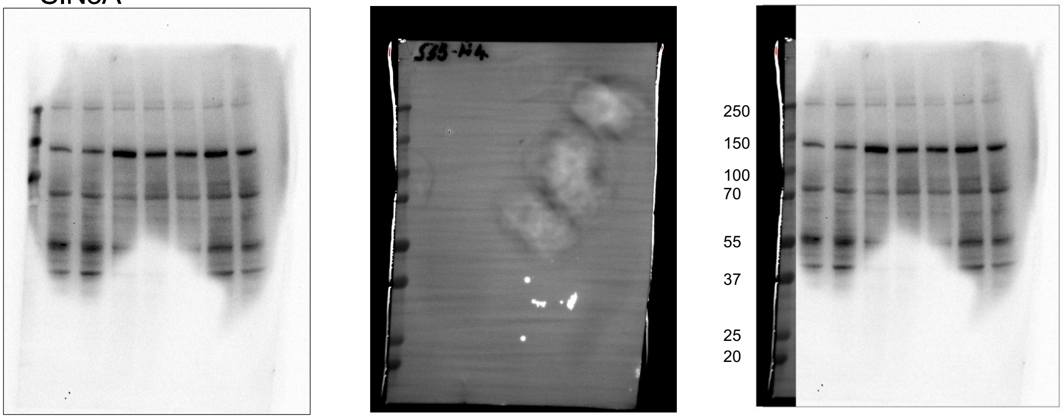

SIN3B-H4

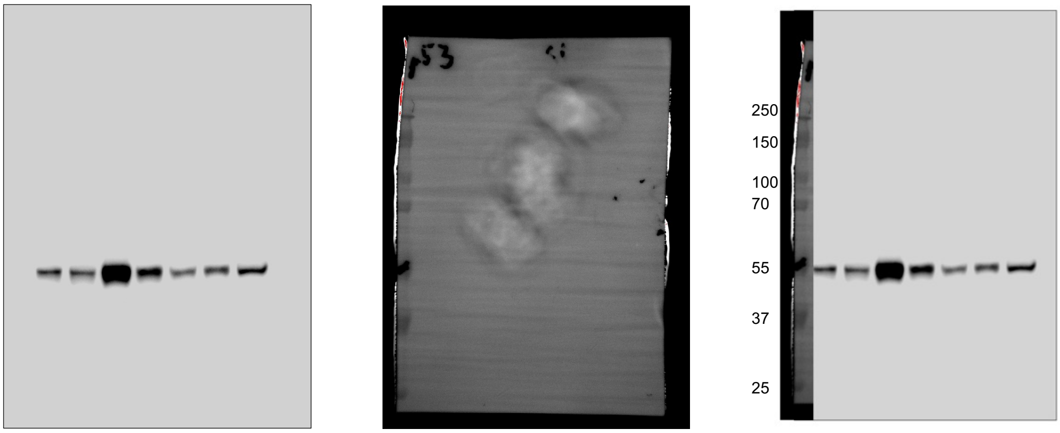

p53

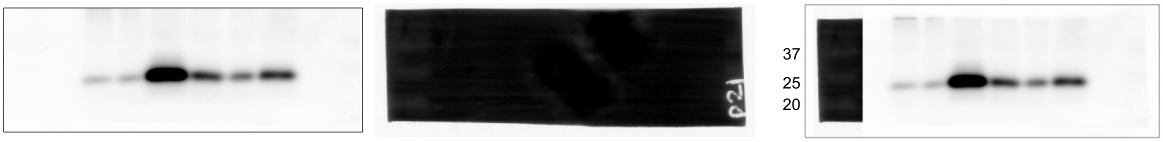

p21

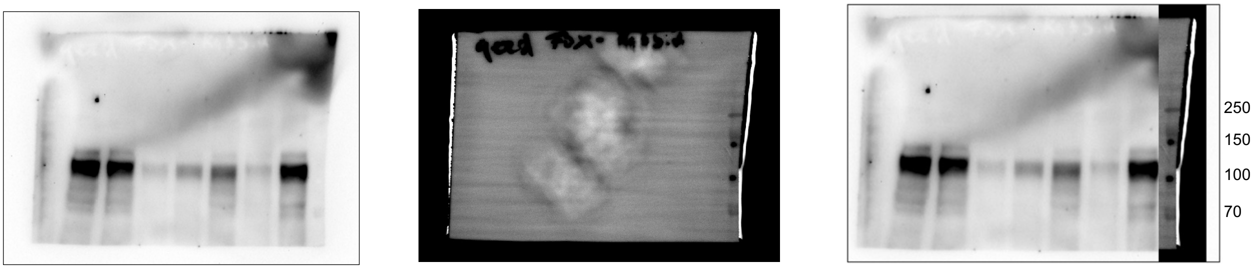

FOXM1

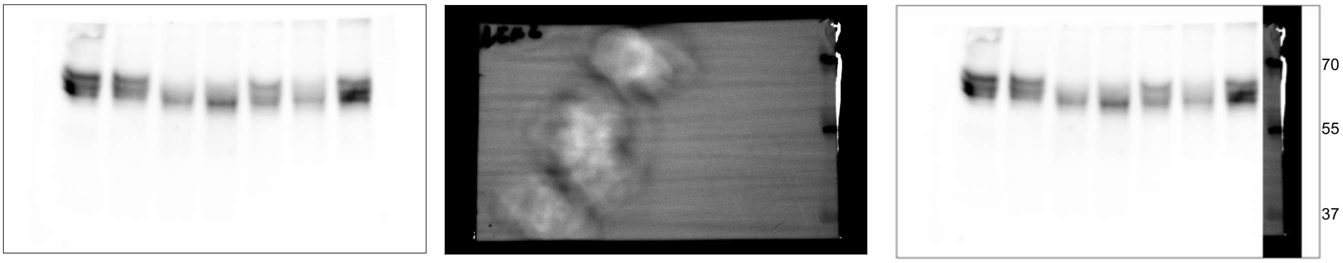

NEK2

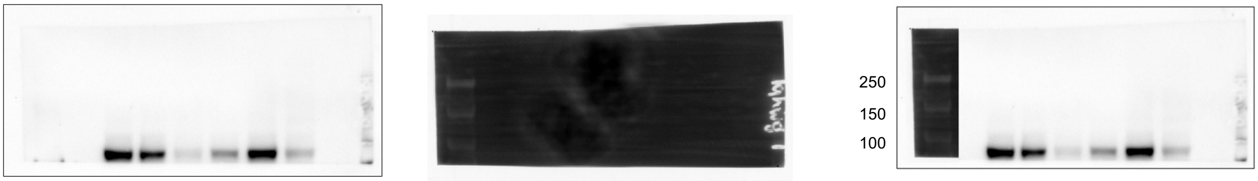

B-MYB

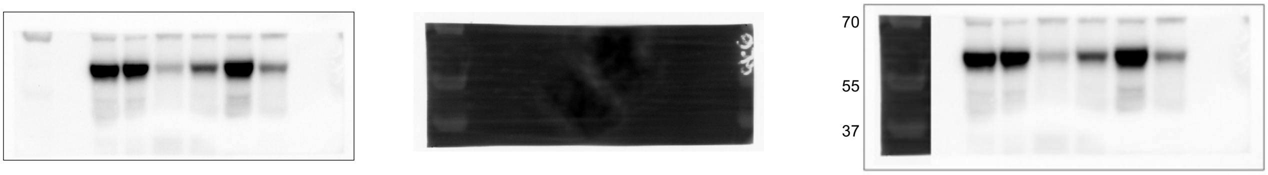

CDC6

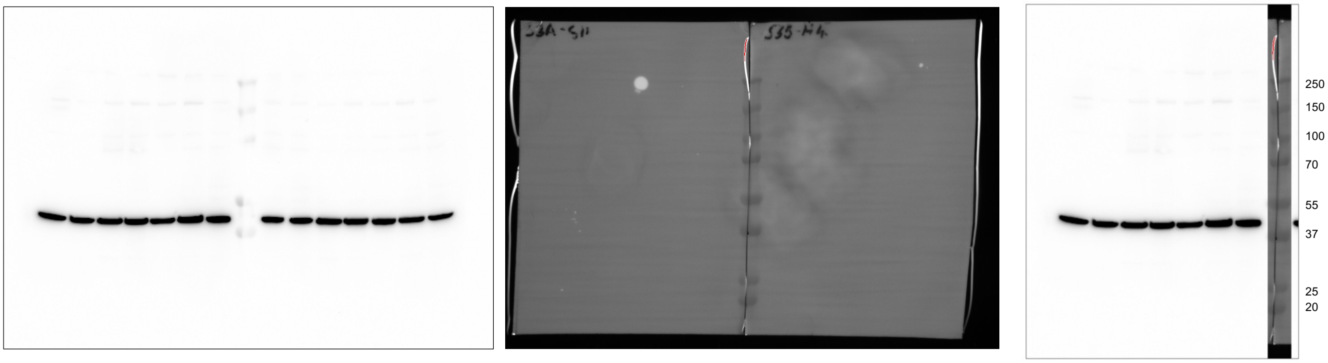

actin

Uncropped blots - Supplementary Fig. 2c

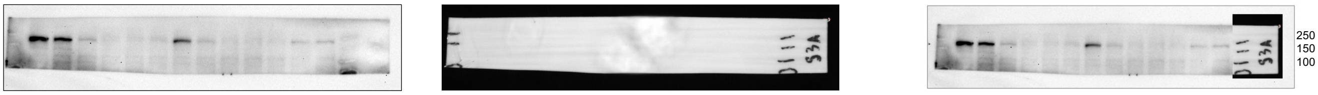

SIN3A

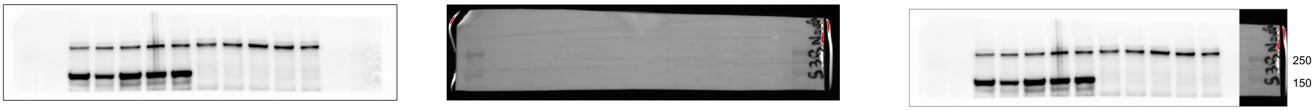

SIN3B - polyclonal

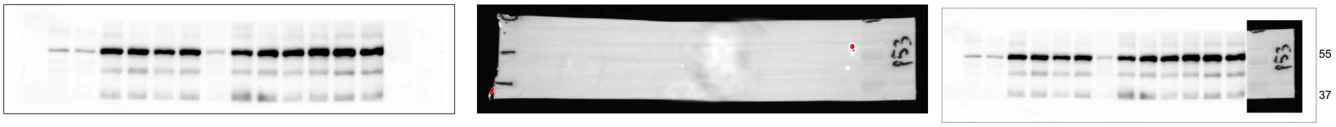

p53

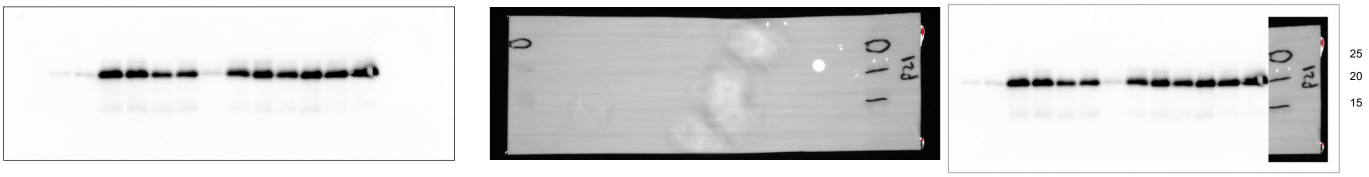

p21

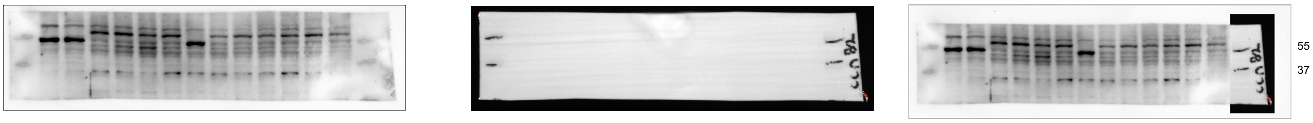

CCNB2

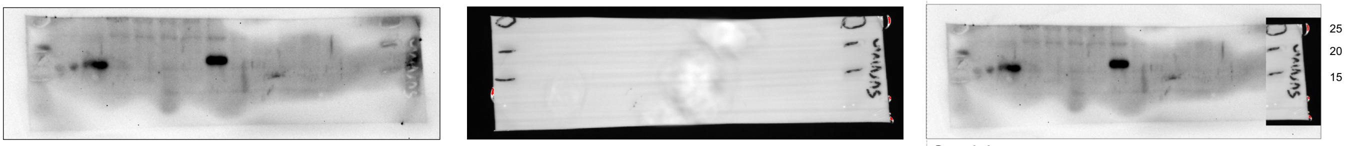

Survivin

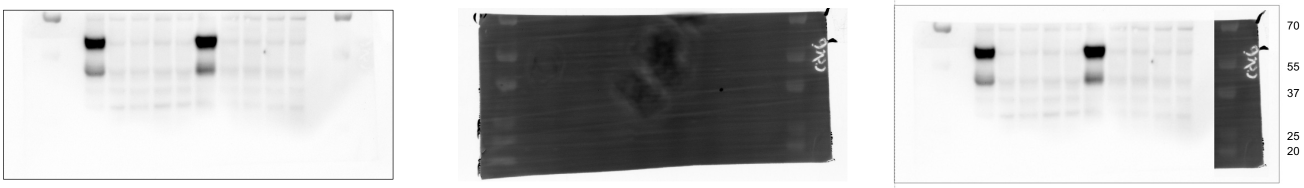

CDC6

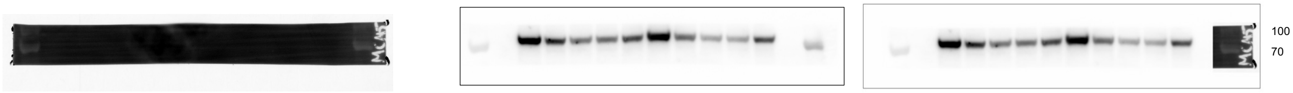

MCM5

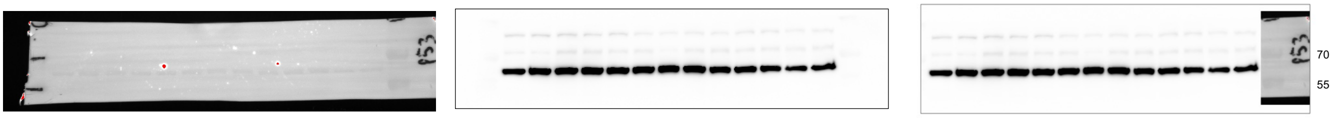

actin

Uncropped blots - Supplementary Fig. 3b and 3d

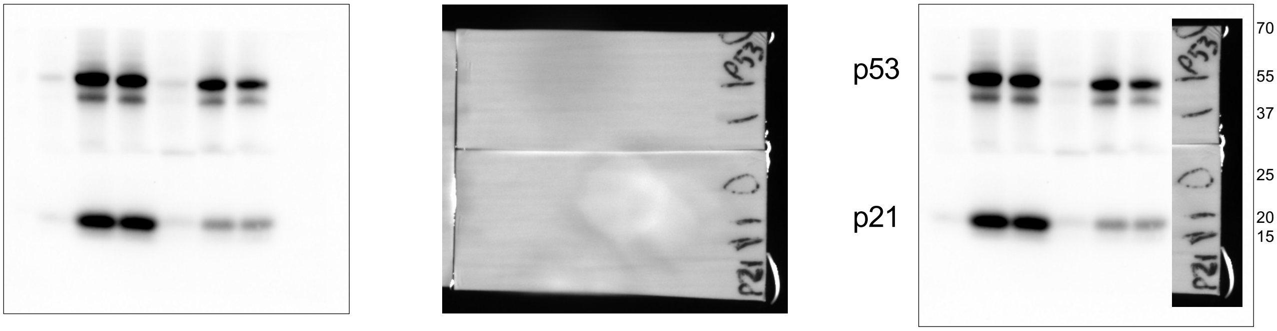

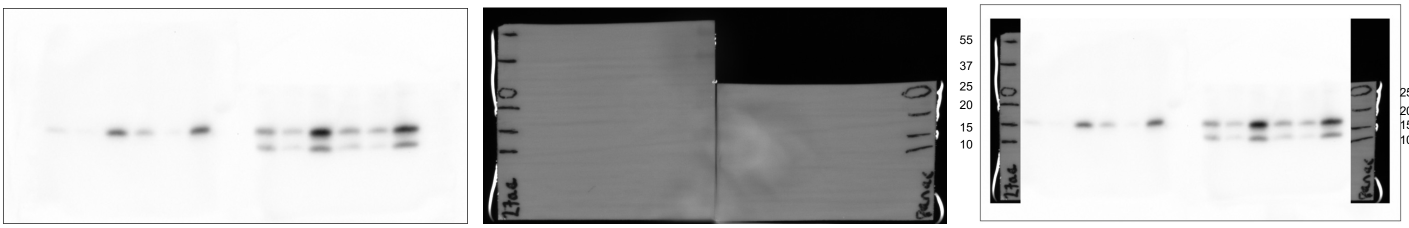

H3K27ac H3ac

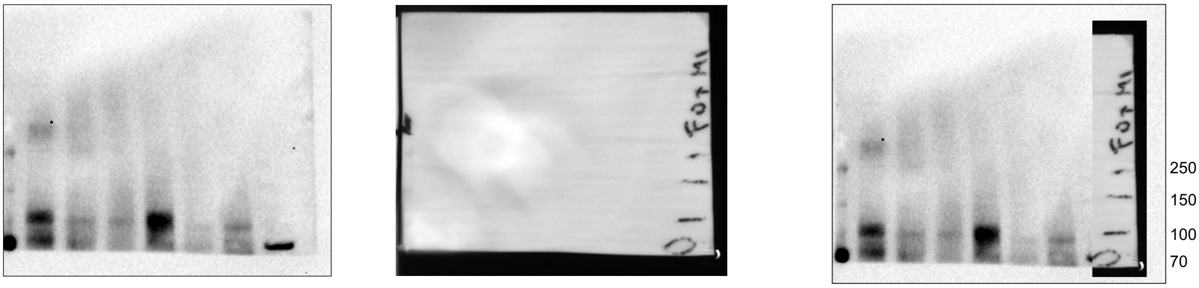

FOXM1

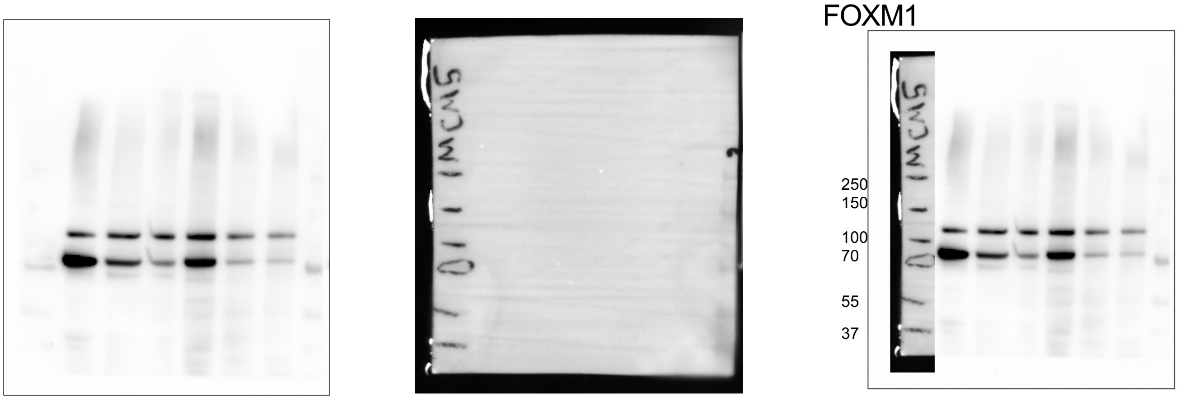

MCM5

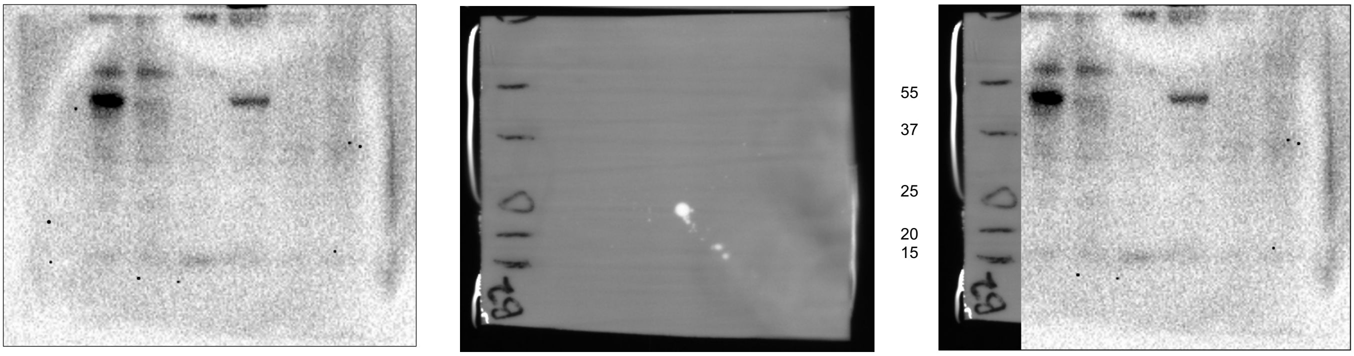

CCNB2

Uncropped blots - Supplementary Fig. 3b and 3d

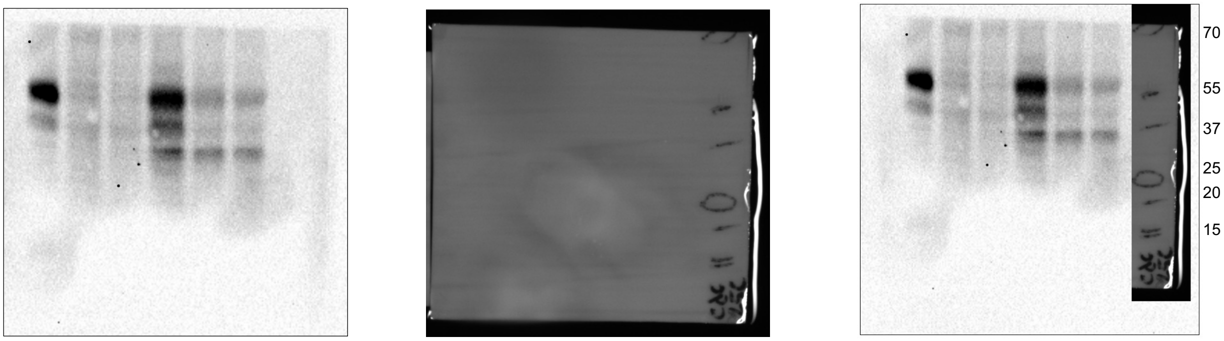

CDC25C

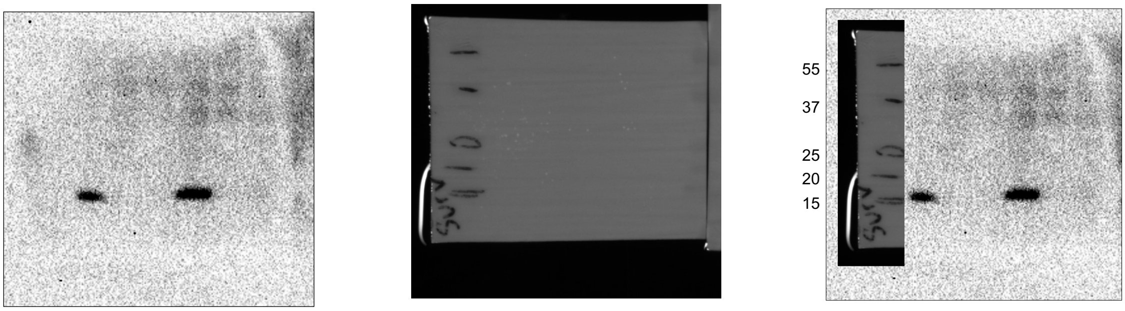

Survivin

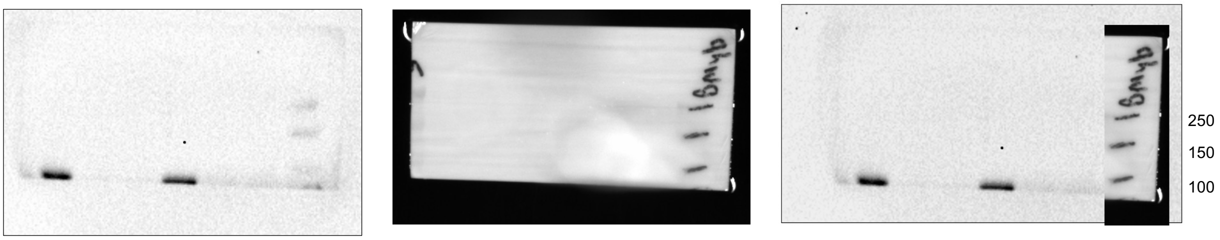

B-MYB

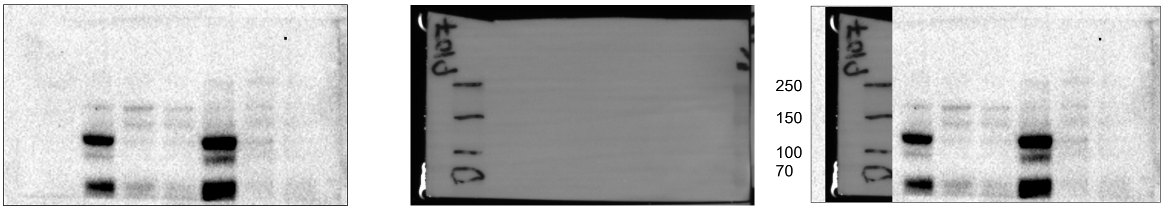

p107

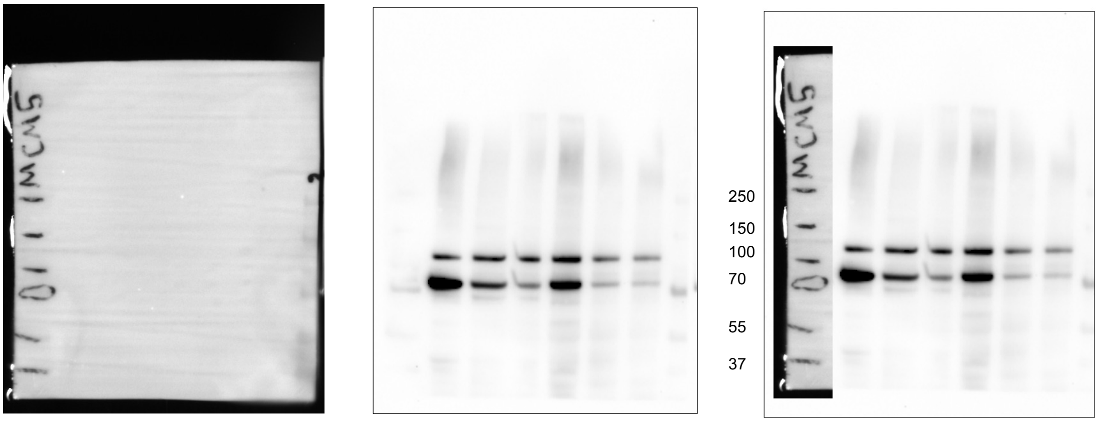

MCM5

Uncropped blots - Supplementary Fig. 3b and 3d

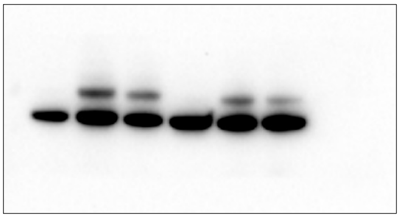

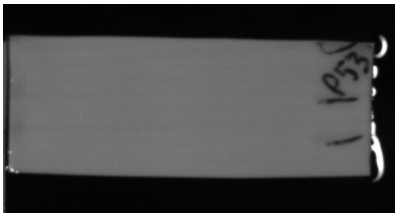

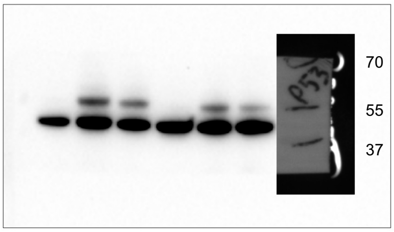

actin
