# Supplementary material for: HDAC activity is dispensable for repression of cell-cycle genes by DREAM and E2F:RB complexes
Source: Nat Commun. 2024 May 24;15:4450. doi: 10.1038/s41467-024-48724-0 (PMC11126580; doi:10.1038/s41467-024-48724-0)
Supplement: Supplementary file 3 — Description of Additional Supplementary Files [file 41467_2024_48724_MOESM3_ESM.pdf]

Description of Additional Supplementary Files

File Name: Supplementary Data 1
Description: Sequences of oligonucleotides used in this study.

File Name: Supplementary Data 2
Description: Antibodies used in this study.

File Name: Supplementary Data 3
Description: G1/S, G2/M, and non-DREAM/non-cell cycle regulated control genes selected to generate Fig. 1.

File Name: Supplementary Data 4
Description: Differentially expressed genes in SIN3B-/-, SIN3A knockdown, and SIN3B-/-;SIN3A knockdown HCT116 cells.
