# Supplementary material for: HDAC activity is dispensable for repression of cell-cycle genes by DREAM and E2F:RB complexes
Source: Nat Commun. 2024 May 24;15:4450. doi: 10.1038/s41467-024-48724-0 (PMC11126580; doi:10.1038/s41467-024-48724-0)
Supplement: Supplementary file 8 — Reporting Summary [file 41467_2024_48724_MOESM8_ESM.pdf]

Corresponding author(s): Seth M. Rubin, Gerd A. Müller

Last updated by author(s): Apr 8, 2024

Reporting Summary

Nature Portfolio wishes to improve the reproducibility of the work that we publish. This form provides structure for consistency and transparency in reporting. For further information on Nature Portfolio policies, see our Editorial Policies and the Editorial Policy Checklist.

Statistics

For all statistical analyses, confirm that the following items are present in the figure legend, table legend, main text, or Methods section.

n/a	Confirmed	
☐	☒	The exact sample size (*n*) for each experimental group/condition, given as a discrete number and unit of measurement
☐	☒	A statement on whether measurements were taken from distinct samples or whether the same sample was measured repeatedly
☐	☒	The statistical test(s) used AND whether they are one- or two-sided<br>*Only common tests should be described solely by name; describe more complex techniques in the Methods section.*
☒	☐	A description of all covariates tested
☐	☒	A description of any assumptions or corrections, such as tests of normality and adjustment for multiple comparisons
☐	☒	A full description of the statistical parameters including central tendency (e.g. means) or other basic estimates (e.g. regression coefficient) AND variation (e.g. standard deviation) or associated estimates of uncertainty (e.g. confidence intervals)
☐	☒	For null hypothesis testing, the test statistic (e.g. *F*, *t*, *r*) with confidence intervals, effect sizes, degrees of freedom and *P* value noted<br>*Give P values as exact values whenever suitable.*
☒	☐	For Bayesian analysis, information on the choice of priors and Markov chain Monte Carlo settings
☒	☐	For hierarchical and complex designs, identification of the appropriate level for tests and full reporting of outcomes
☒	☐	Estimates of effect sizes (e.g. Cohen's *d*, Pearson's *r*), indicating how they were calculated

Our web collection on statistics for biologists contains articles on many of the points above.

Software and code

Policy information about availability of computer code

Data collection	qPCR data were collected on a Quantstudio3 cycler with firmware version 1.3.3.
Data analysis	PRISM 8.2.1, R 4.2.3, Venny 2.1.0, TFEA.ChIP, ShinyGO 0.77, trimgalore version 0.6.10, cutadapt version 4.2, fastqc v0.12.1, GNU parallel, segemehl version 0.3.4, featureCounts version 2.0.3, DESeq2. Flow cytometry data were analyzed with FlowJo version 10.8.2 (BD Biosciences).

For manuscripts utilizing custom algorithms or software that are central to the research but not yet described in published literature, software must be made available to editors and reviewers. We strongly encourage code deposition in a community repository (e.g. GitHub). See the Nature Portfolio guidelines for submitting code & software for further information.

Data

Policy information about availability of data

All manuscripts must include a data availability statement. This statement should provide the following information, where applicable:

- Accession codes, unique identifiers, or web links for publicly available datasets
- A description of any restrictions on data availability
- For clinical datasets or third party data, please ensure that the statement adheres to our policy

RNA-Seq data generated for this publication were deposited at GEO GSE240734.

Research involving human participants, their data, or biological material

Policy information about studies with human participants or human data. See also policy information about sex, gender (identity/presentation), and sexual orientation and race, ethnicity and racism.

Reporting on sex and gender	N/A
Reporting on race, ethnicity, or other socially relevant groupings	N/A
Population characteristics	N/A
Recruitment	N/A
Ethics oversight	N/A

Note that full information on the approval of the study protocol must also be provided in the manuscript.

Field-specific reporting

Please select the one below that is the best fit for your research. If you are not sure, read the appropriate sections before making your selection.

[x] Life sciences   [ ] Behavioural & social sciences   [ ] Ecological, evolutionary & environmental sciences

For a reference copy of the document with all sections, see nature.com/documents/nr-reporting-summary-flat.pdf

Life sciences study design

All studies must disclose on these points even when the disclosure is negative.

Sample size	No sample size calculation was performed. Experiments were repeated with biological samples that were collected in triplicate or duplicate with more than one clone. These sample sizes were chosen, because they are typical in the field.
Data exclusions	RNA-seq analysis were perfomed with three biological replicates for wild-type, SIN3B-/-, SIN3A knockdown, and SIN3B-/-;SIN3A knockdown cells. One of the wild-type samples had to be excluded because it had a very high adapter content. The final mappable reads of that sample were only a fraction of the other samples. PCA analysis revealed that this sample is a severe outlier, accounting for over 50% of the variance between 12 replicates and 4 conditions. We therefore excluded that wild-type sample from the further analysis.
Replication	Number of replicates is stated in the figure captions.
Randomization	Not applicable. The experimental protocols used in this study do not present any obvious need for randomization. Experiments are performed on cell populations that are identical before treatment, and a subset of cells are used for treatment or control.
Blinding	All experiments were performed without blinding. Data were collected and analyzed on all samples (different treatments and controls) at the same time and using unbiased, quantitative measurements. For these reasons, we deemed blinding of sample identity unnecessary.

Reporting for specific materials, systems and methods

We require information from authors about some types of materials, experimental systems and methods used in many studies. Here, indicate whether each material, system or method listed is relevant to your study. If you are not sure if a list item applies to your research, read the appropriate section before selecting a response.

Materials & experimental systems

n/a	Involved in the study	
[ ]	[x]	Antibodies
[ ]	[x]	Eukaryotic cell lines
[x]	[ ]	Palaeontology and archaeology
[x]	[ ]	Animals and other organisms
[x]	[ ]	Clinical data
[x]	[ ]	Dual use research of concern
[x]	[ ]	Plants

Methods

n/a	Involved in the study	
[x]	[ ]	ChIP-seq
[ ]	[x]	Flow cytometry
[x]	[ ]	MRI-based neuroimaging

Antibodies

Antibodies used	Acetyl-Histone H3 (Lys27) (D5E4) #8173, Cell Signaling; B-MYB, clone LX015.1, #MILL-MABE886, Millipore; CDC25C (H-6), #sc-13138, Santa Cruz Biotechnology; CDC6 (180.2), #sc-9964, Santa Cruz Biotechnology; Cyclin B2 (A-2), #sc-28303, Santa Cruz Biotechnology; FoxM1 (D12D5), #5436S, Cell Signaling; HDAC1 (10E2), #sc-81598, Santa Cruz Biotechnology; Histone H3 (D1H2), #4499, Cell Signaling; Histone H3ac (pan-acetyl), #61637, Active Motif; LIN37-T3 (custom-made at Pineda Antikörper-Service, Berlin, Germany); LIN9, #A300-BL2981, Bethyl Laboratories Inc; MCM5 (E-10), #sc-165994, Santa Cruz Biotechnology; MKLP-1 /KIF23 (C-12), #sc-390113, Santa Cruz Biotechnology; NEK2 (D-8), #sc-55601, Santa Cruz Biotechnology; p107 (SD9), #sc-250, Santa Cruz Biotechnology; p21 (F-5), #sc-6246, Santa Cruz Biotechnology; p53 (DO-1), #sc-126, Santa Cruz Biotechnology; Rabbit (DA1E) mAb IgG XP® Isotype Control, #66362S, Cell Signaling; RB (C-2), #sc-74562, Santa Cruz Biotechnology; RBBP4, #A301-206A, Bethyl Laboratories Inc; SIN3A (D1B7), #7691S, Cell Signaling; SIN3A (G11), #sc-5299, Santa Cruz Biotechnology; SIN3B (H-4), #sc-13145, Santa Cruz Biotechnology; SIN3B polyclonal, #NBP2-13309, Novus Biologicals LLC; ß-Actin- Direct-Blot™ HRP, #664804, BioLegend; Survivin (D-8), #sc-17779, Santa Cruz Biotechnology; Tri-Methyl-Histone H3 (Lys27) (C36B11), #9733, Cell Signaling; UBE2C (B-8), #sc-271050, Santa Cruz Biotechnology
Validation	Commercially available antibodies were validated by manufacturer for immunoprecipitation and immunoblotting. Data are included on manufacturers web site and typically contain a western blot and example immunoprecipitation. Citations are also included on the relevant web pages. Validation by gene knockout of the custom made LIN37-T3 antibody is reported in PMID: 28920576

Eukaryotic cell lines

Policy information about cell lines and Sex and Gender in Research

Cell line source(s)	HCT116, C2C12, T98G, A549, and BJ-hTERT cells were purchased from the ATCC.
Authentication	Authentication performed by ATCC and according to ATCC verification procedures, which includes Mycoplasma detection, STR profiling, and Sanger sequencing
Mycoplasma contamination	Cells were tested negative for mycoplasma by qPCR following a protocol published in PMID: 11840297
Commonly misidentified lines (See ICLAC register)	none

Flow Cytometry

Plots

Confirm that:

- [ ] The axis labels state the marker and fluorochrome used (e.g. CD4-FITC).
- [ ] The axis scales are clearly visible. Include numbers along axes only for bottom left plot of group (a 'group' is an analysis of identical markers).
- [ ] All plots are contour plots with outliers or pseudocolor plots.
- [x] A numerical value for number of cells or percentage (with statistics) is provided.

Methodology

Sample preparation	EdU assays were performed with the Click-iT® EdU Flow Cytometry Assay Kit (ThermoFisher Scientific, #C10420) according to the manufacturer's information. After the Click reaction, cells were treated with RNAse A (10µg/ml), and DNA was stained with propidium iodide (PI, 10µg/ml). 10,000 cells of each sample were analyzed by flow cytometry (LSR II, BD Biosciences). Data were analyzed with FlowJo v 10.8.2 (BD).
Instrument	LSR II, BD Biosciences
Software	FlowJo 10.8.2
Cell population abundance	na
Gating strategy	EdU-positive cells were gated relative to a negative control (cells w/o EdU incorporation). We are going to provide a figure in the Supplementary information showing the gating strategy.

- [x] Tick this box to confirm that a figure exemplifying the gating strategy is provided in the Supplementary Information.
