# Supplementary material for: HDAC activity is dispensable for repression of cell-cycle genes by DREAM and E2F:RB complexes
Source: Nat Commun. 2024 May 24;15:4450. doi: 10.1038/s41467-024-48724-0 (PMC11126580; doi:10.1038/s41467-024-48724-0)
Supplement: Supplementary file 9 — Source Data [file 41467_2024_48724_MOESM9_ESM.pdf]

Fig. 2a

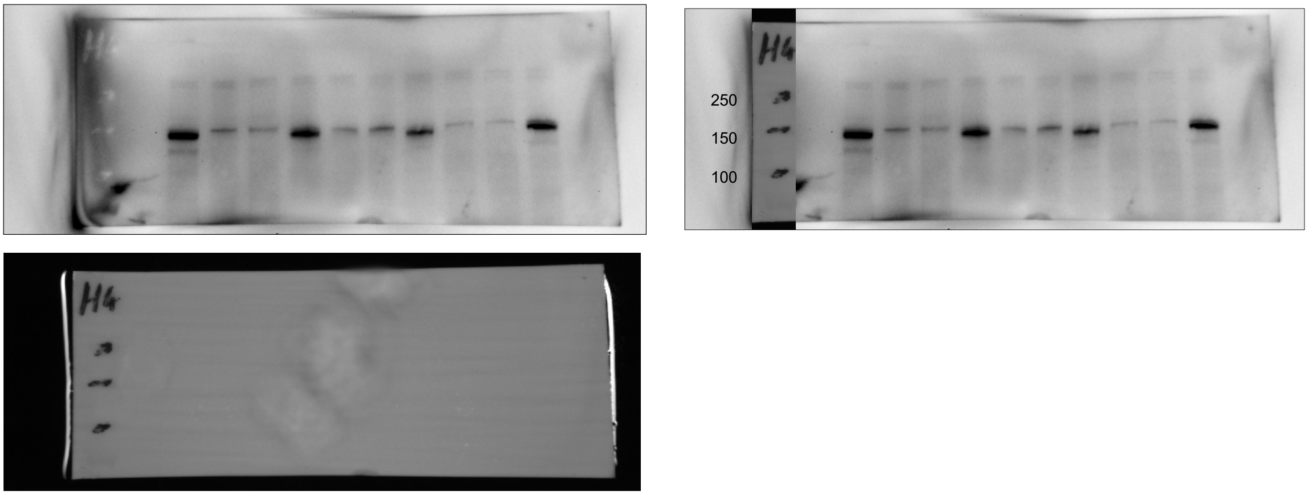

SIN3B-H4, SCBT

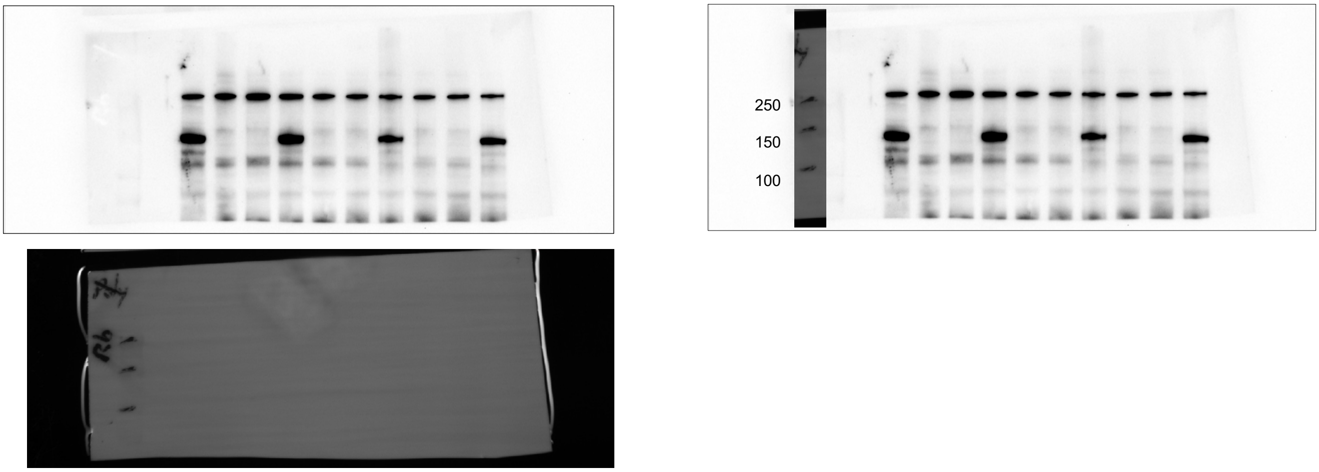

SIN3B polyclonal, Novus

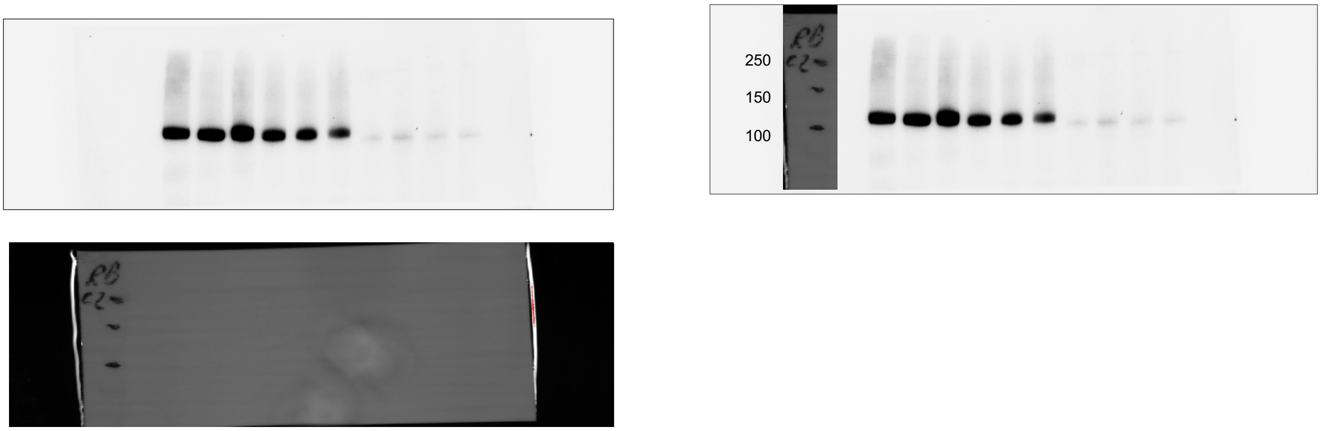

RB-C2, SCBT

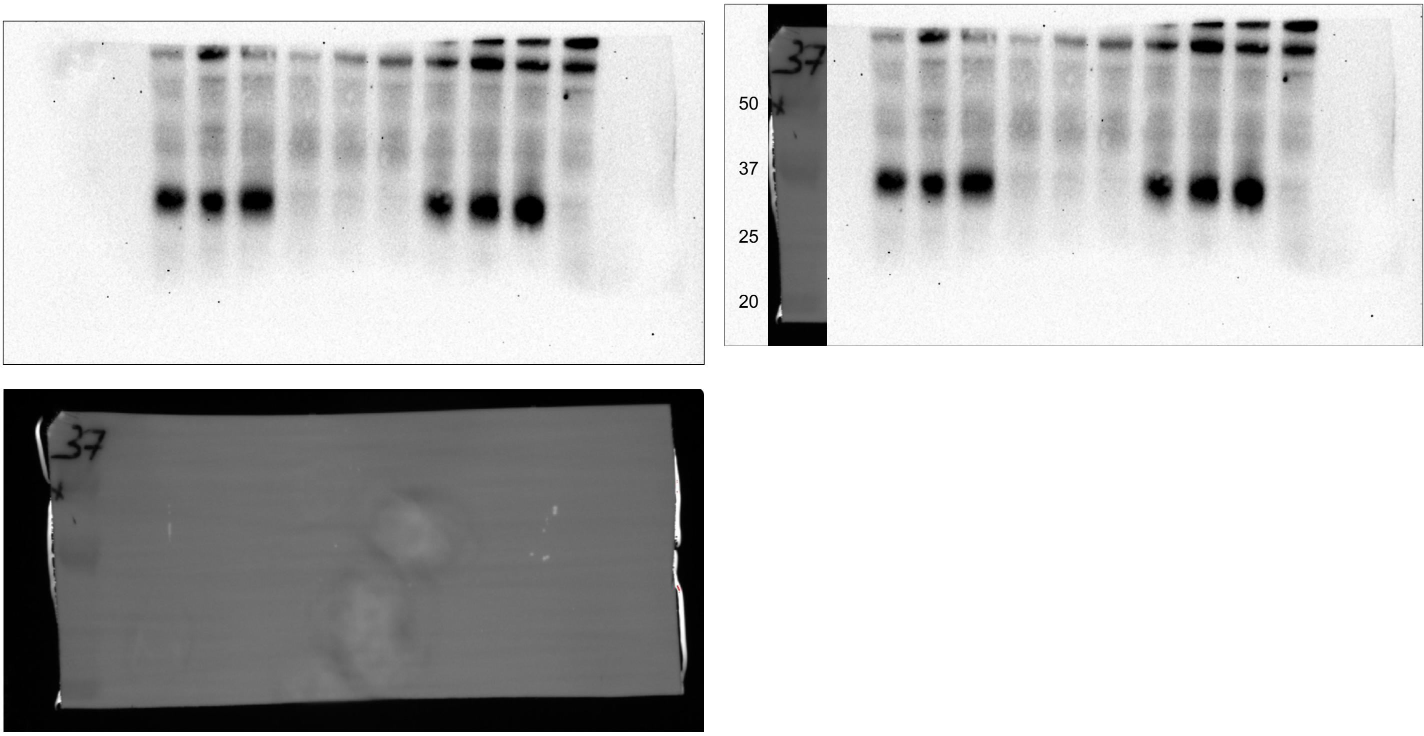

LIN37-T3

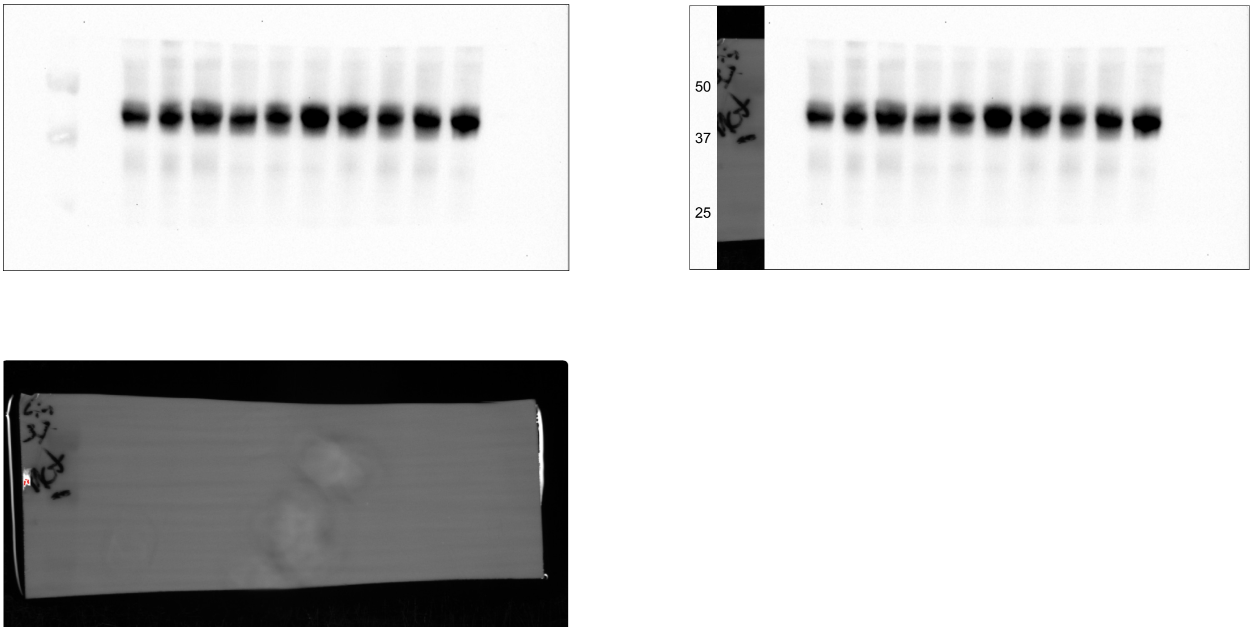

β-actin

Fig. 2d

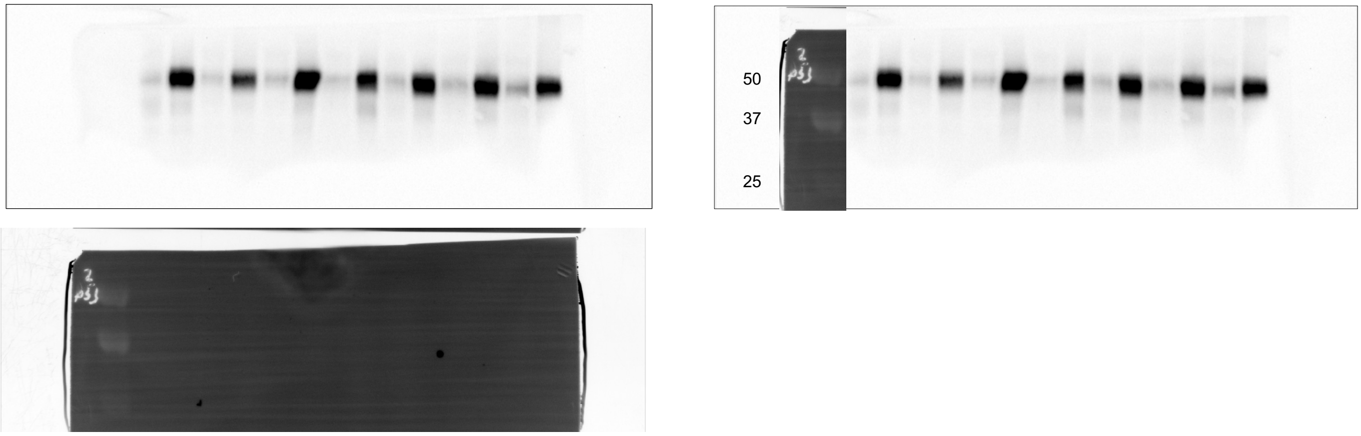

p53, SCBT

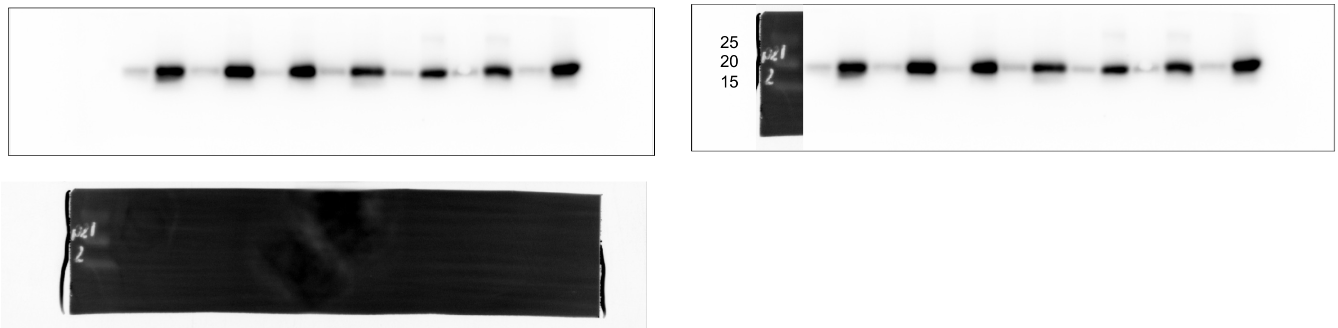

p21, SCBT

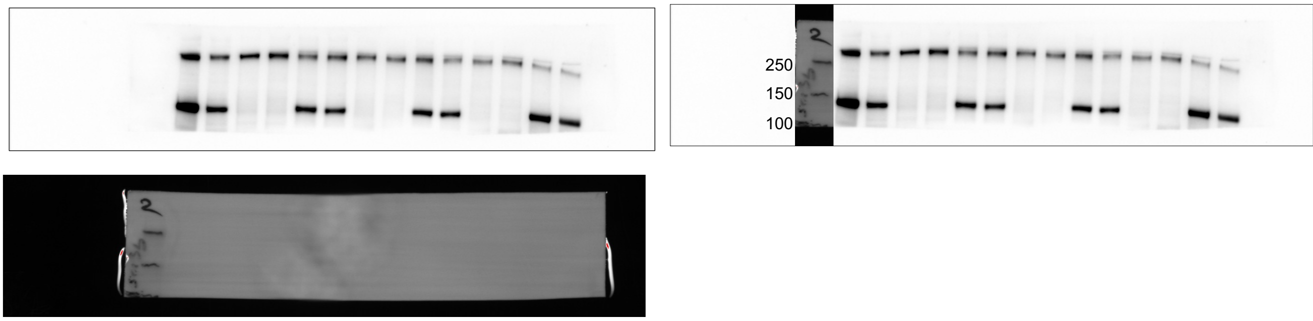

SIN3B polyclonal, Novus

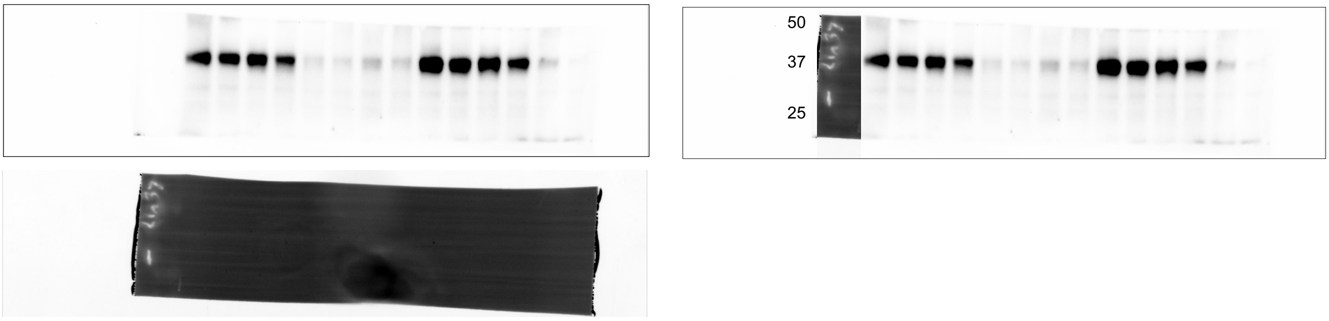

LIN37-T3

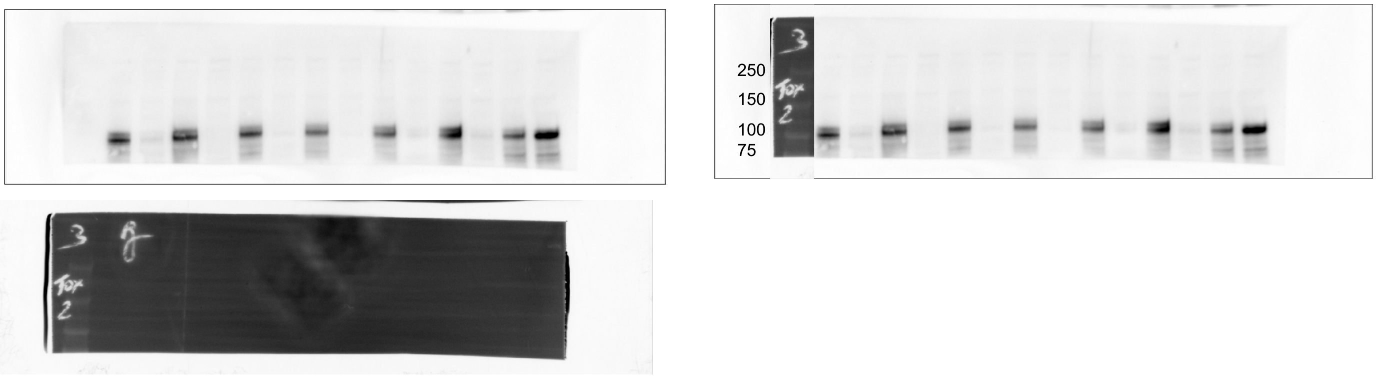

FOXM1, Cell Signaling

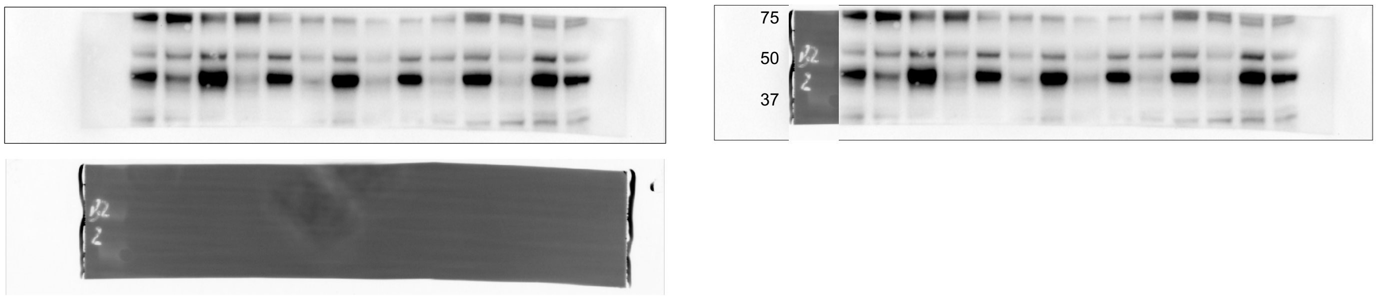

CCNB2, SCBT

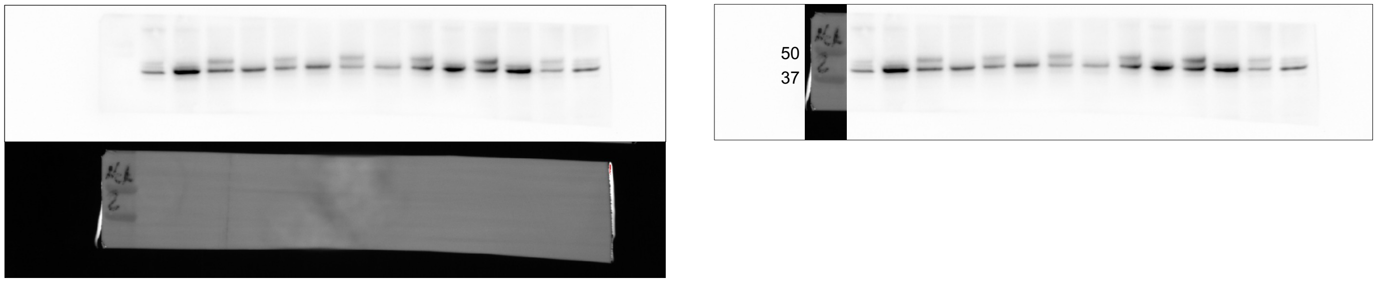

NEK2, SCBT

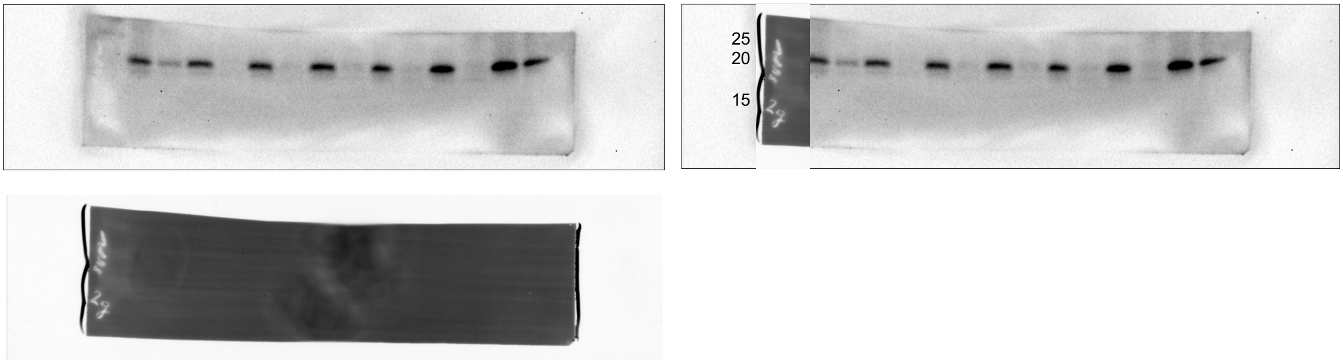

Survivin, SCBT

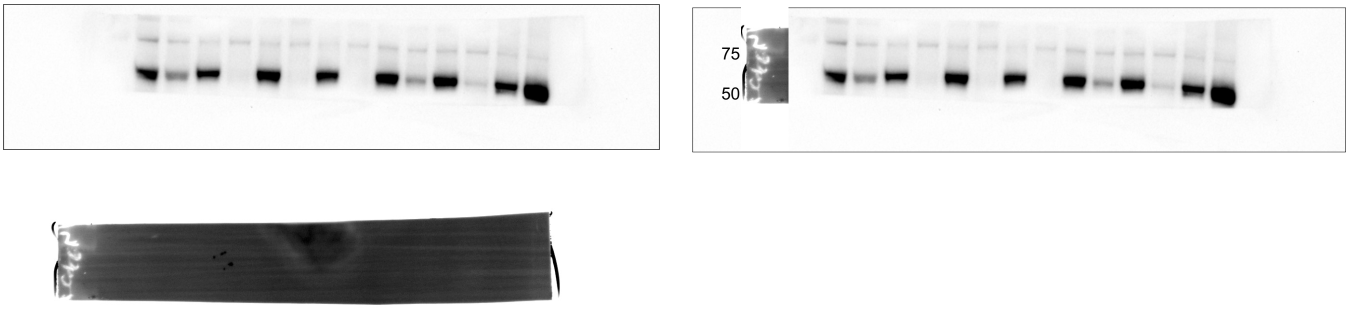

CDC6, SCBT

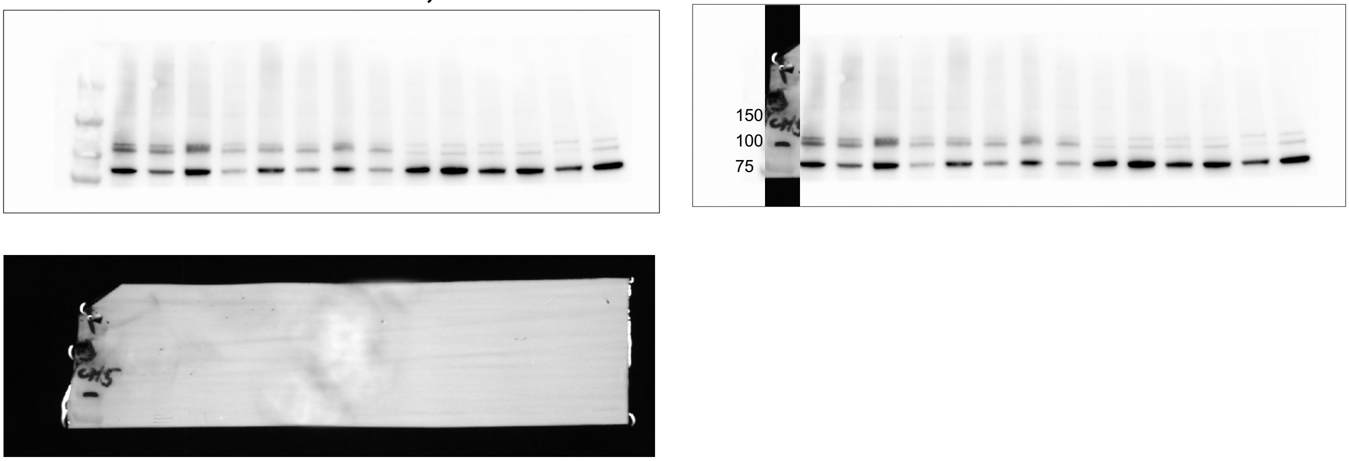

MCM5, SCBT

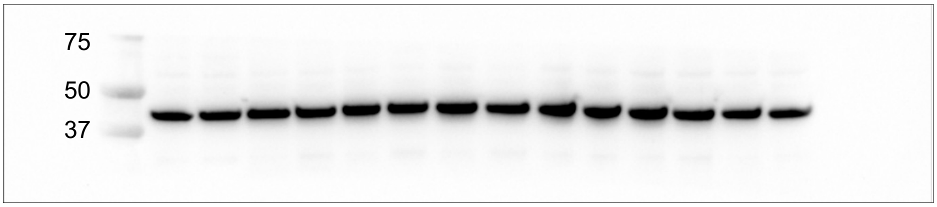

β-actin

Fig. 3b

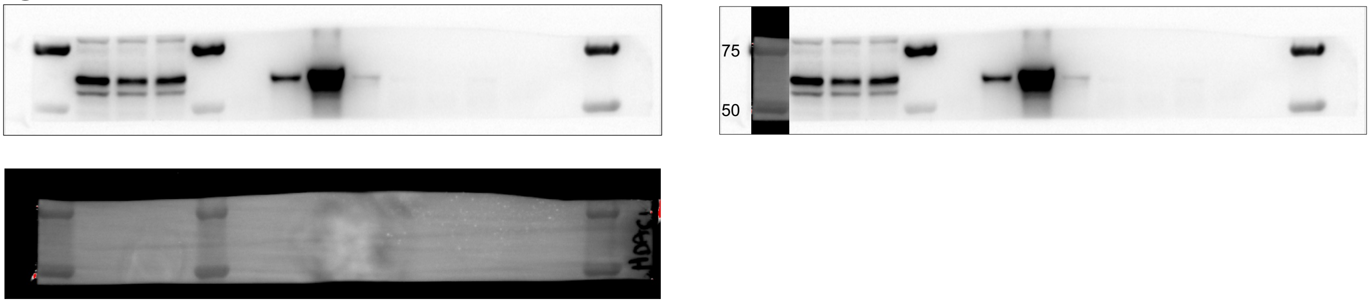

HDAC1, SCBT

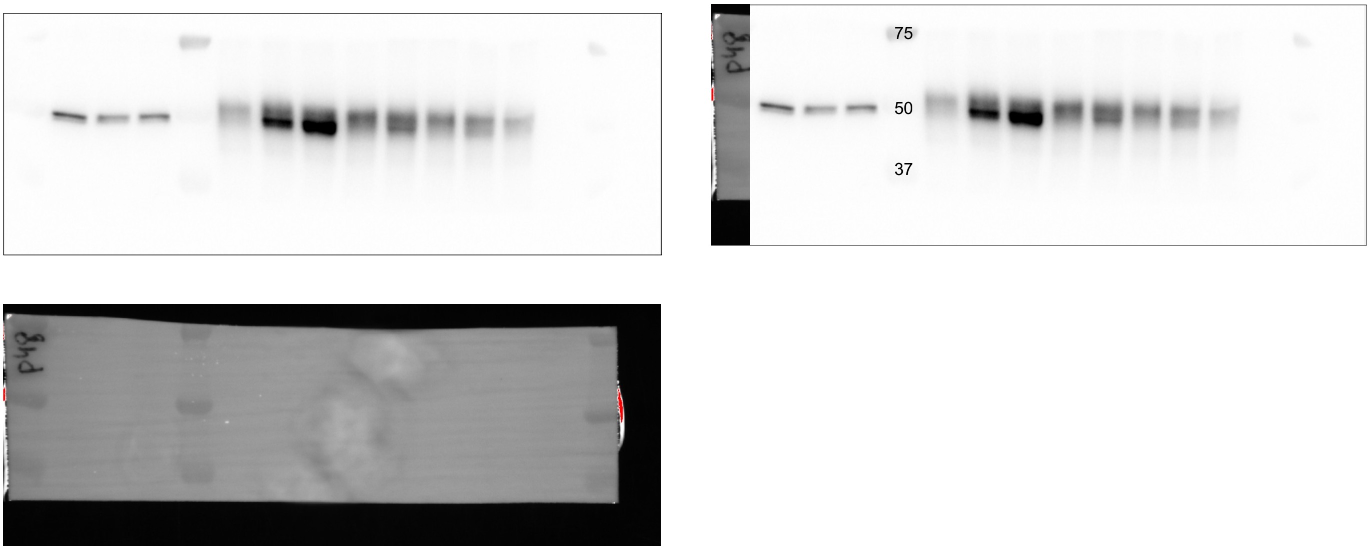

RBBP4, Bethyl

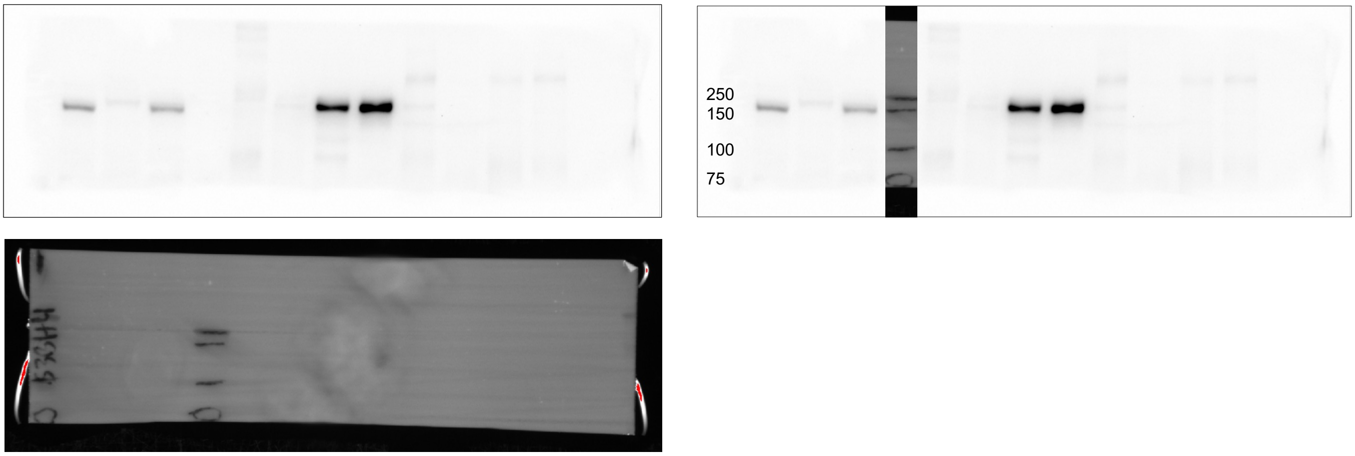

SIN3B-H4, SCBT

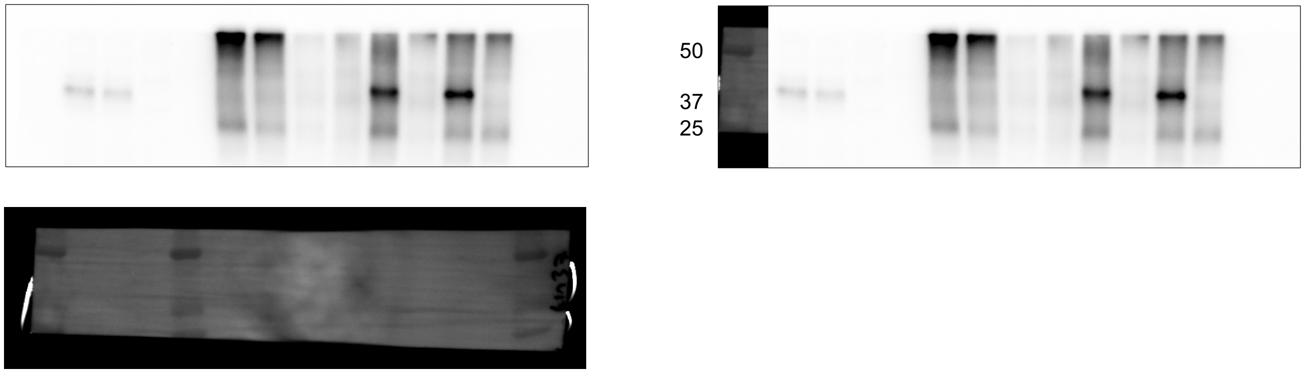

LIN37-T3

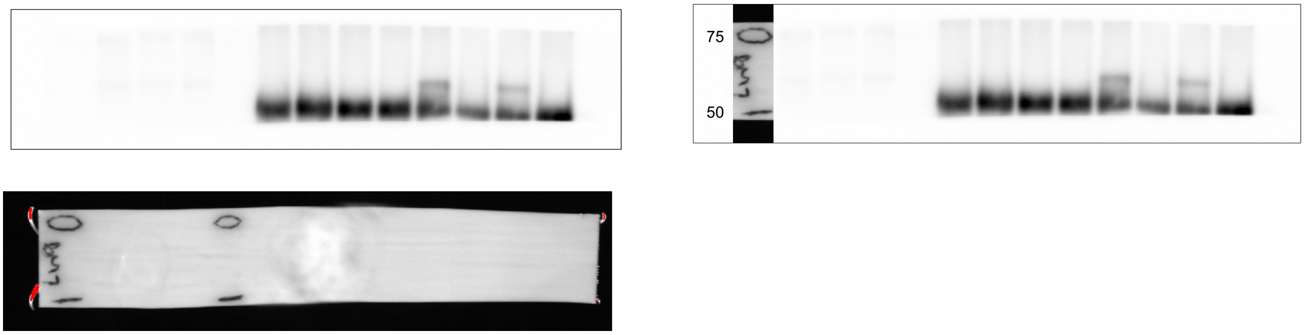

short exposure

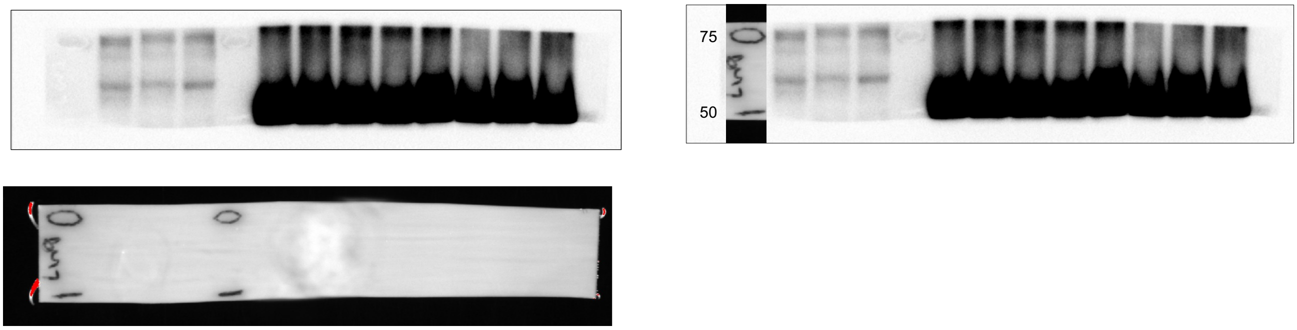

long exposure

LIN9, Bethyl

Fig. 4a

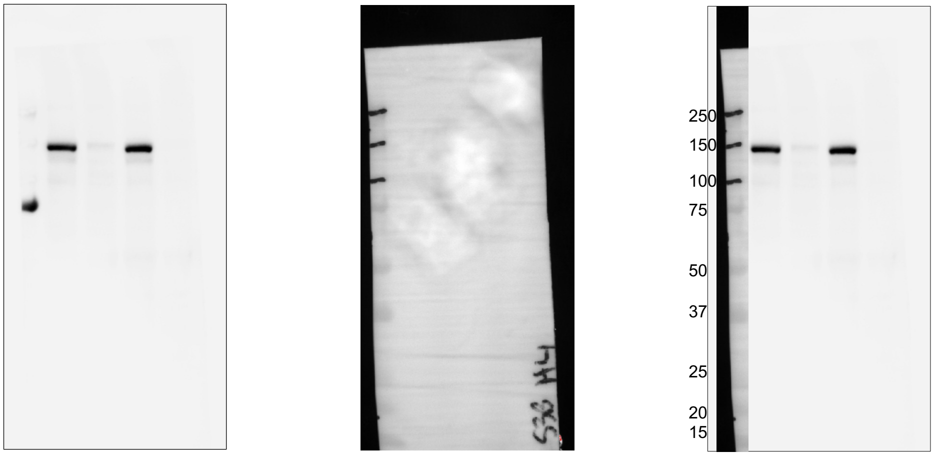

SIN3B-H4, SCBT

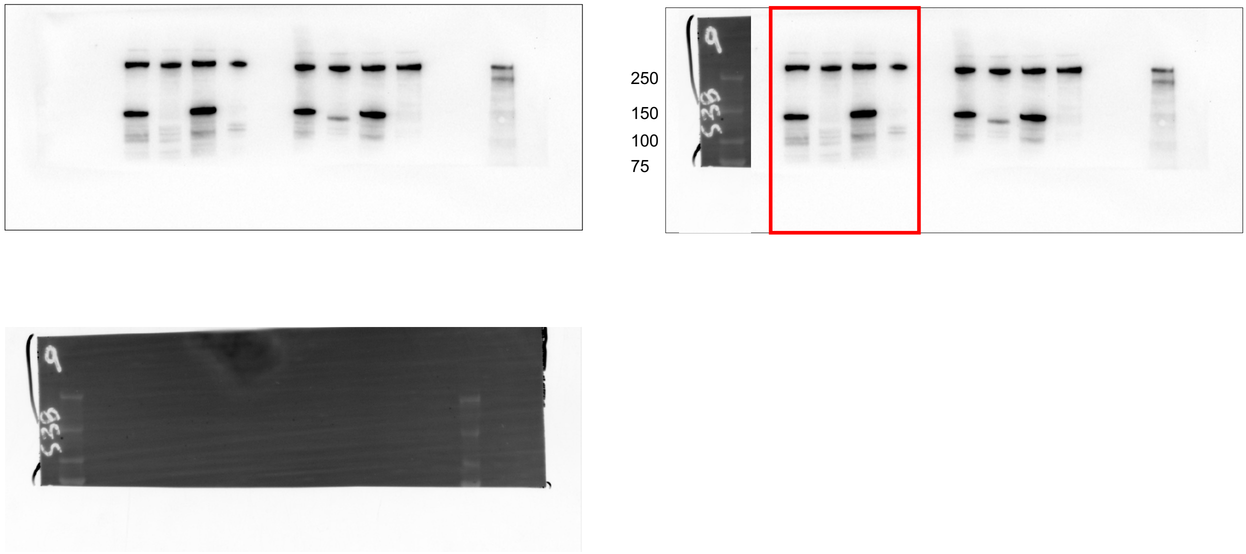

SIN3B polyclonal, Novus

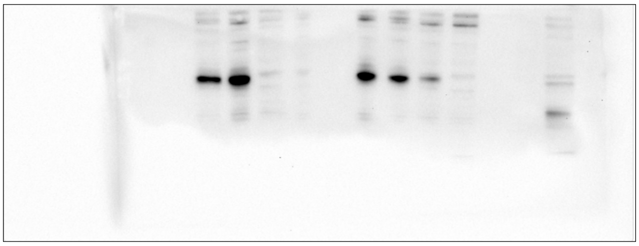

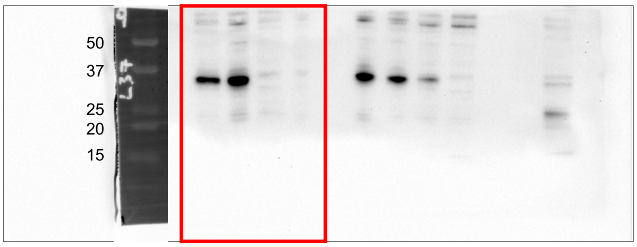

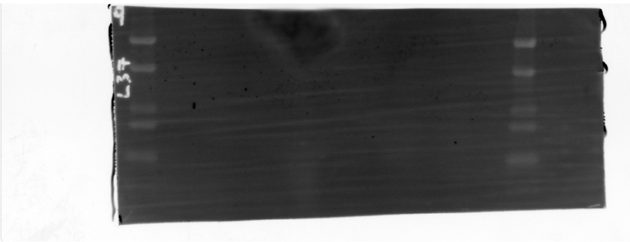

LIN37-T3

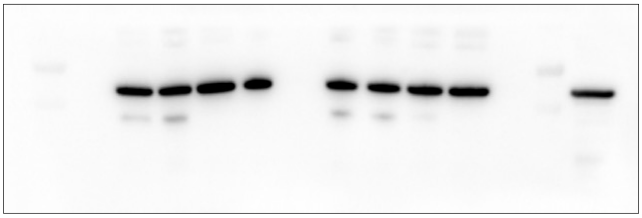

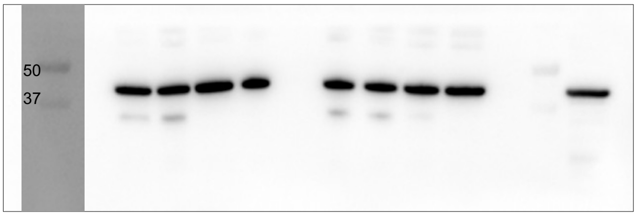

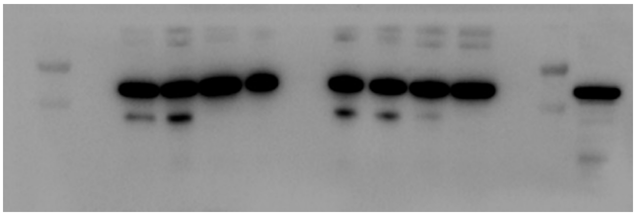

β-actin

Fig. 4c

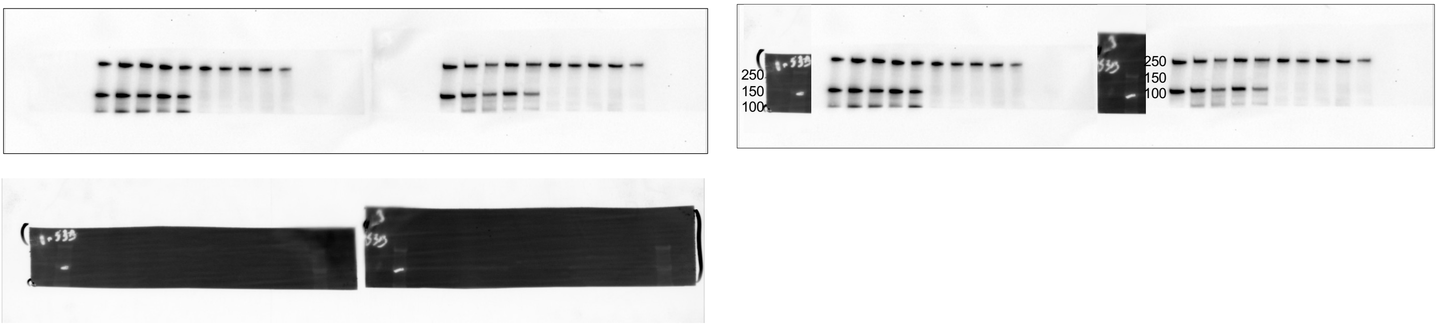

SIN3B polyclonal, Novus

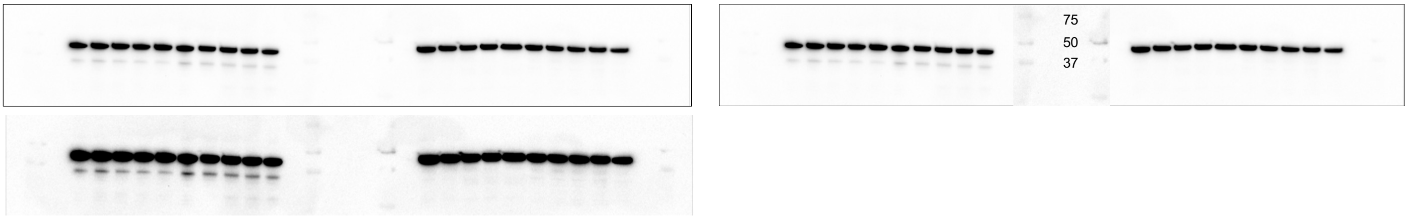

LIN37-T3 + β-actin

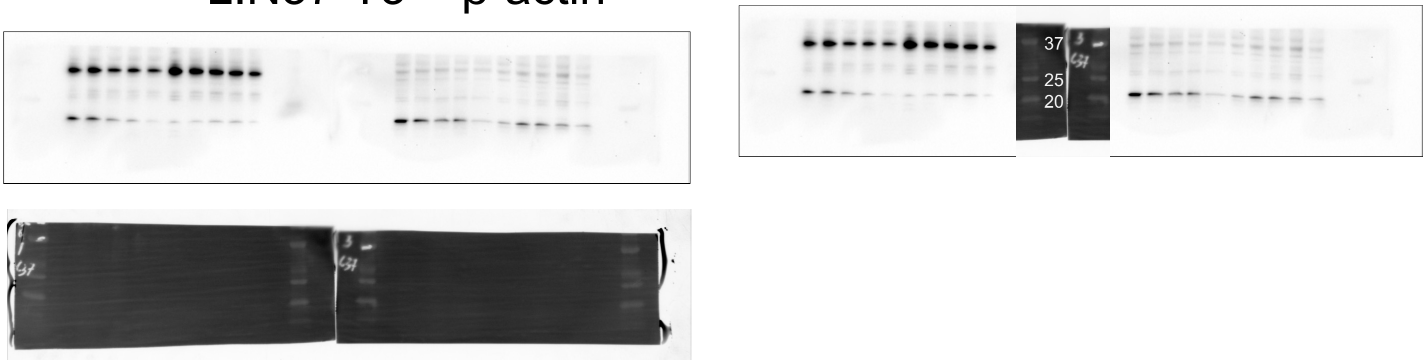

LIN37-T3

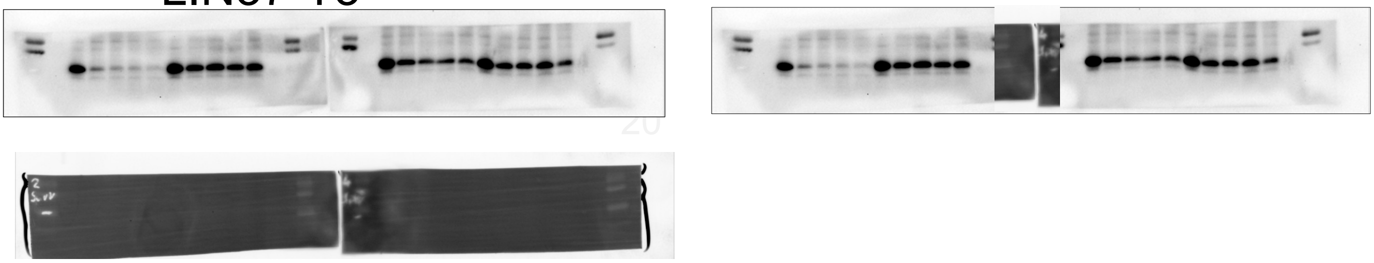

Survivin, SCBT

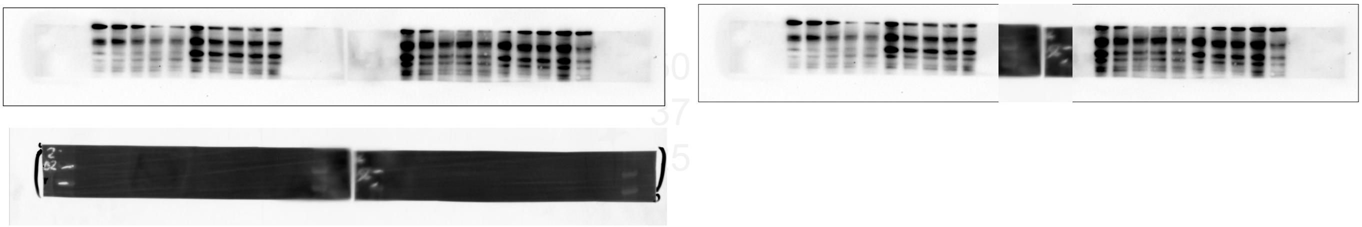

CCNB2, SCBT

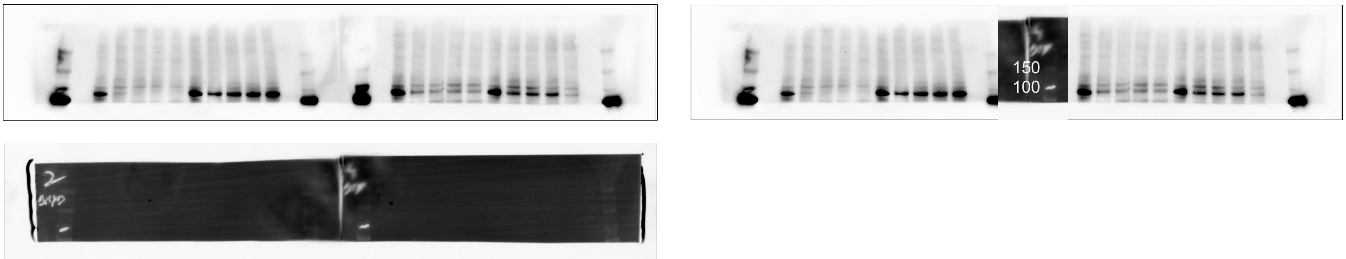

B-MYB, Millipore

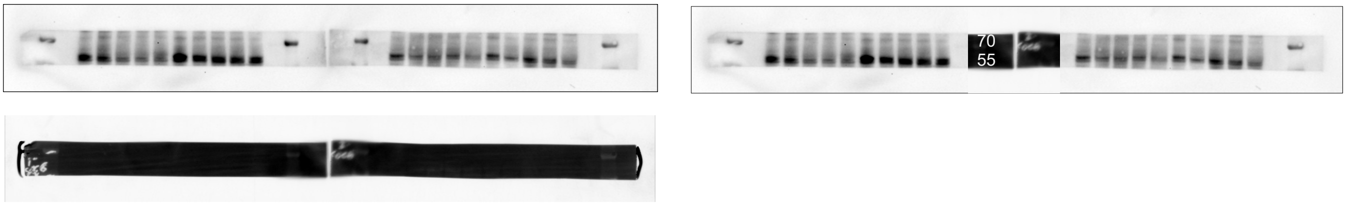

CDC6, SCBT

Fig. 4e

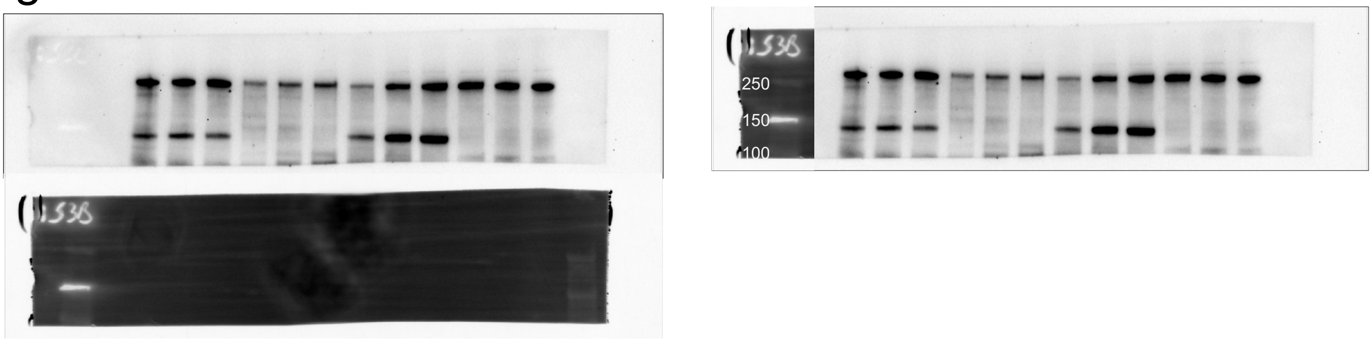

SIN3B polyclonal, Novus

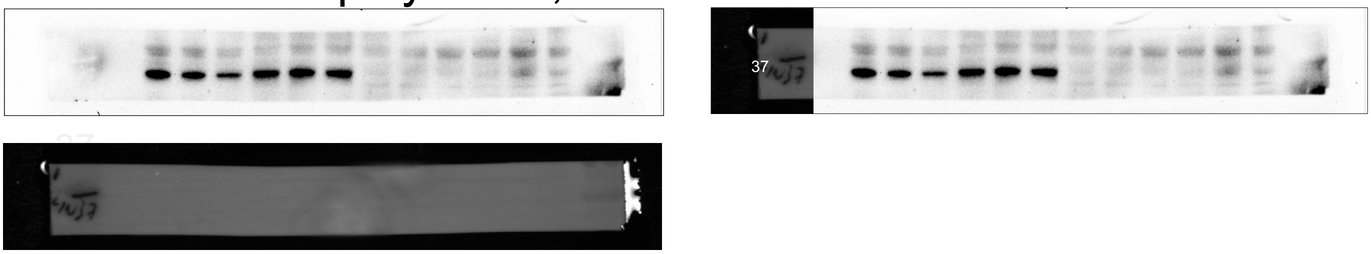

LIN37-T3

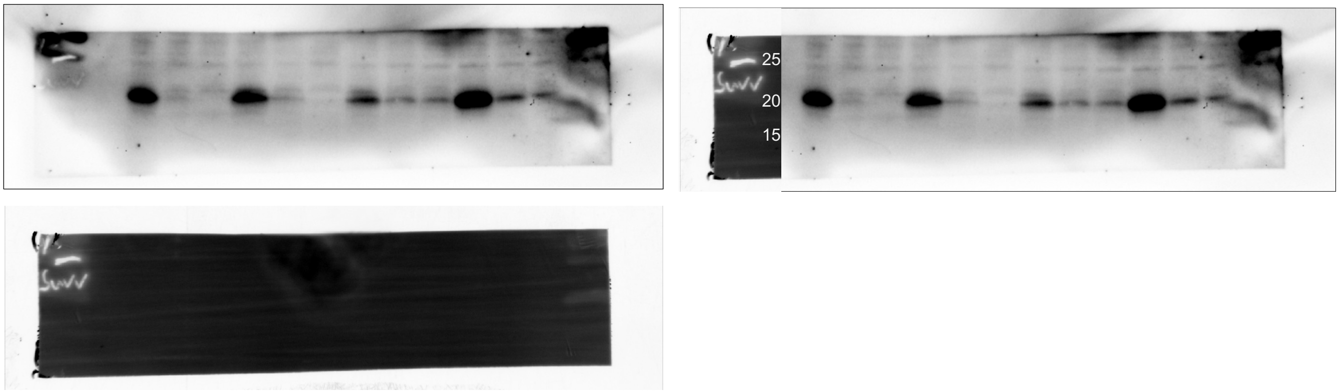

Survivin, SCBT

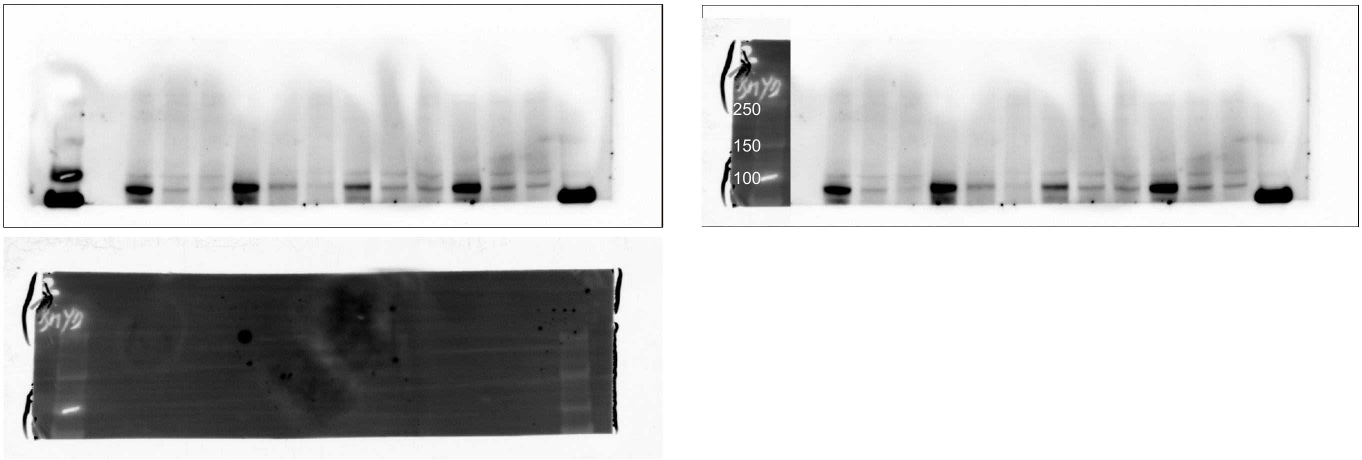

B-MYB, Millipore

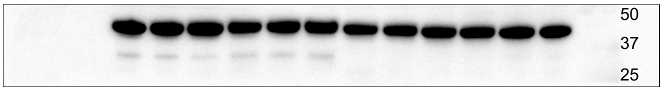

β-actin

Fig. 4g

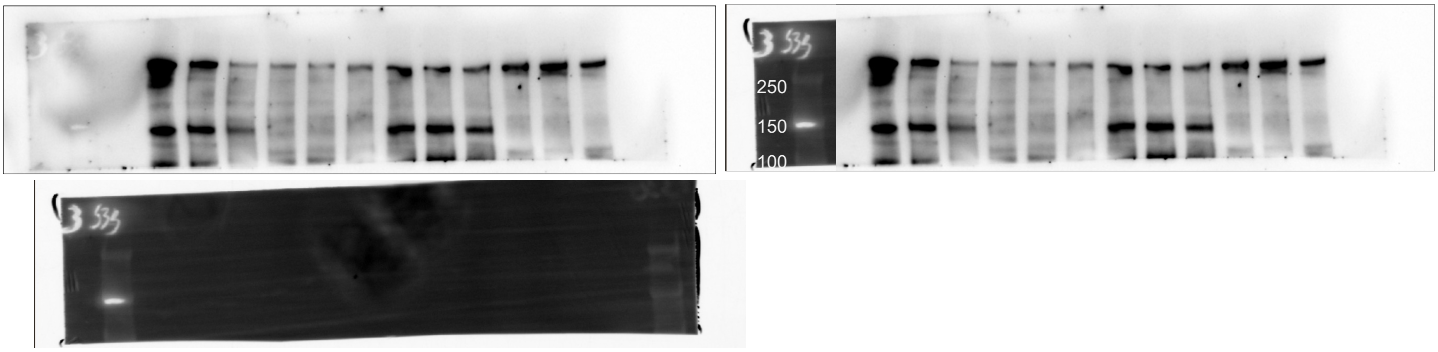

SIN3B polyclonal, Novus

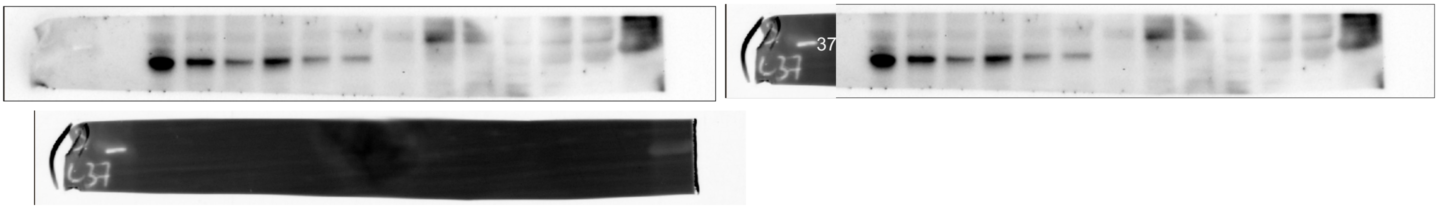

LIN37-T3

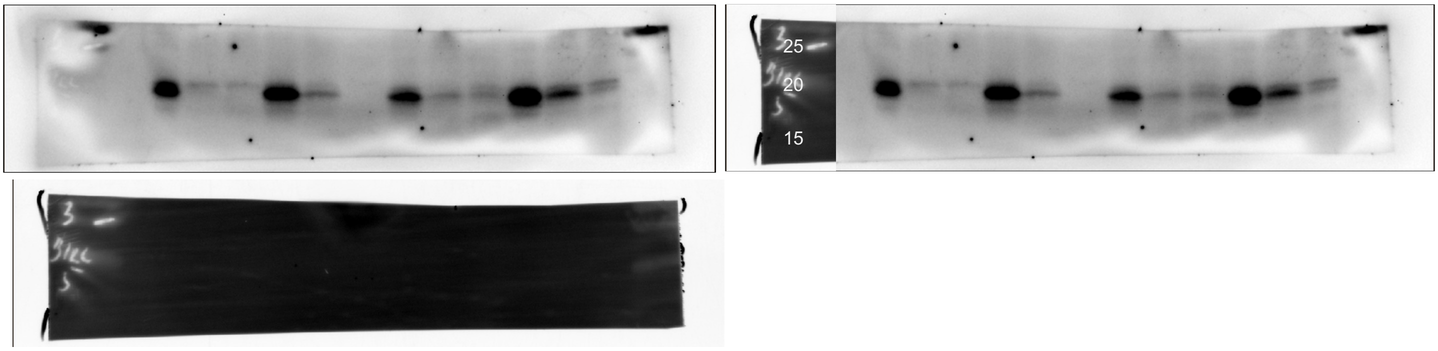

Survivin, SCBT

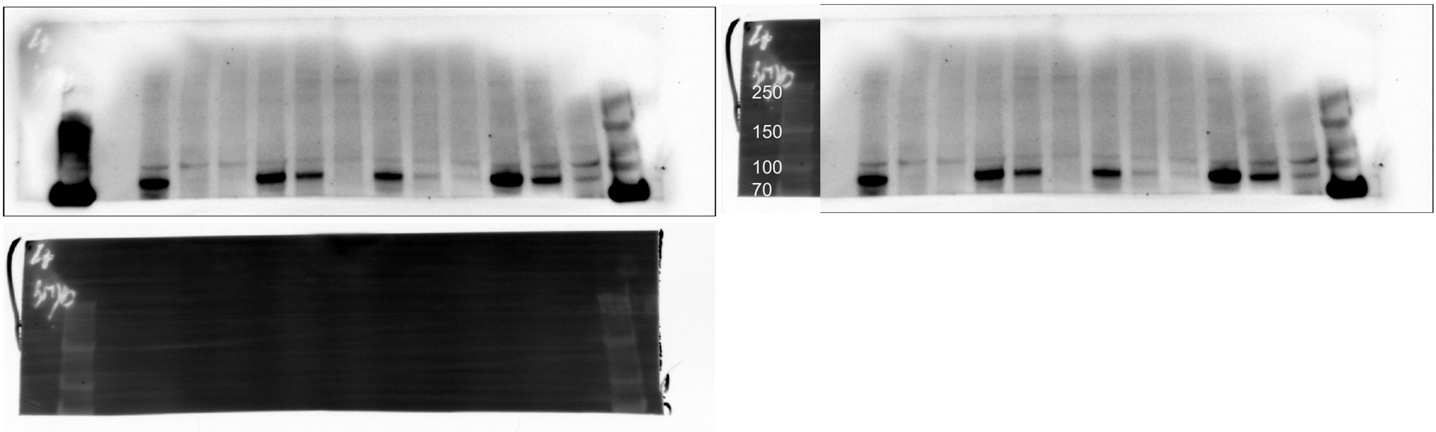

B-MYB, Millipore

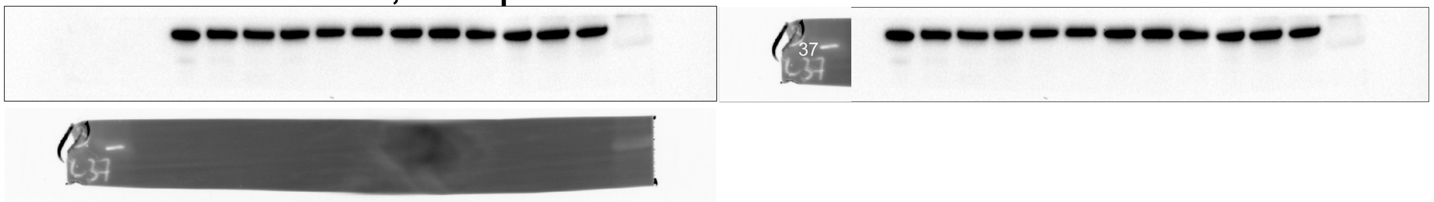

β-actin

Fig. 4j

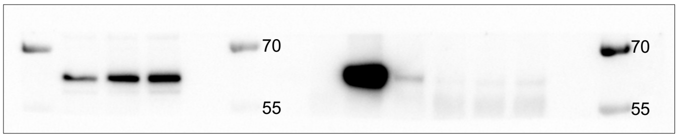

HDAC1, SCBT

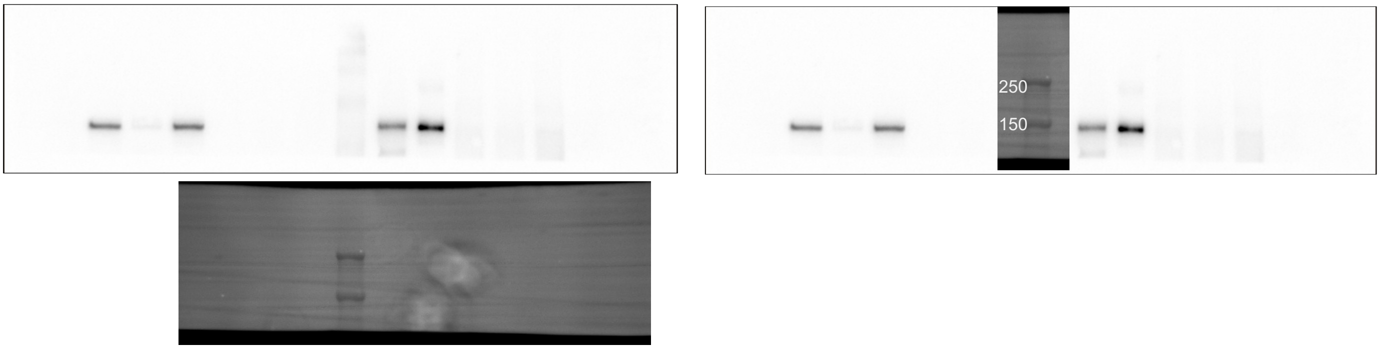

SIN3B-H4, SCBT

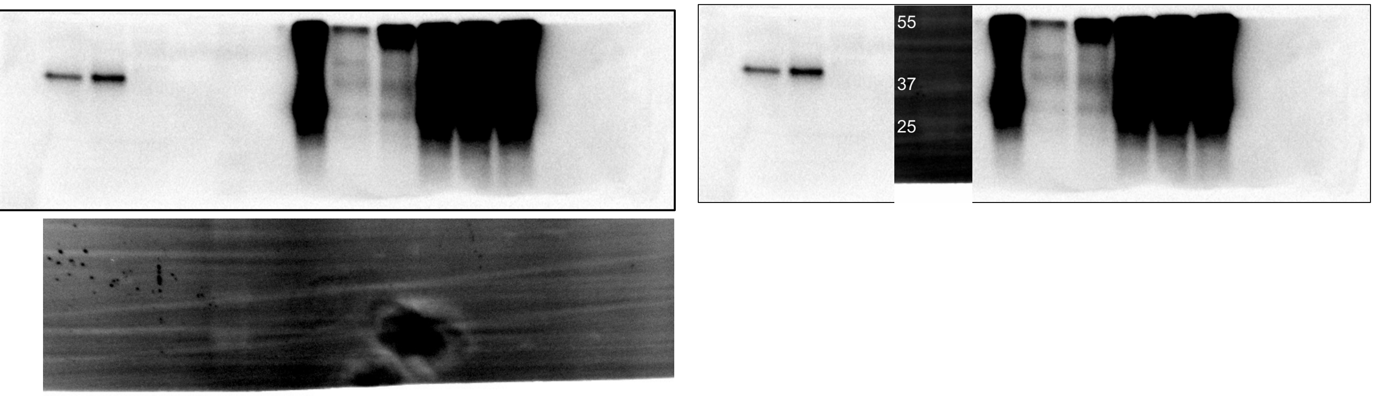

long exposure

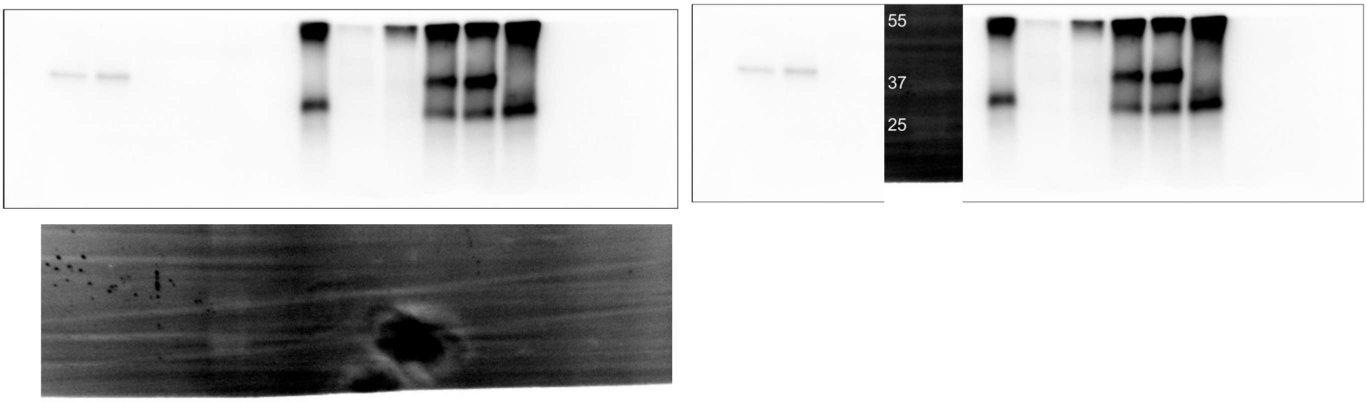

short exposure

LIN37-T3

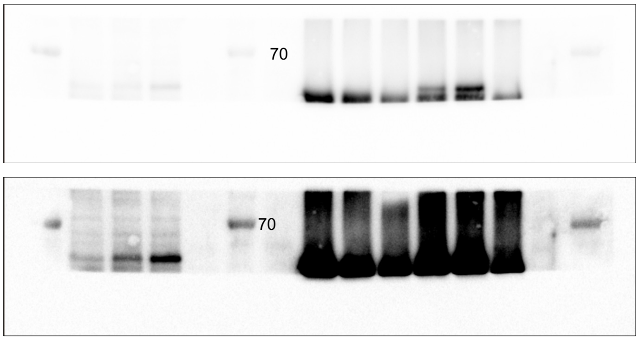

LIN9, Bethyl

Fig. 5a

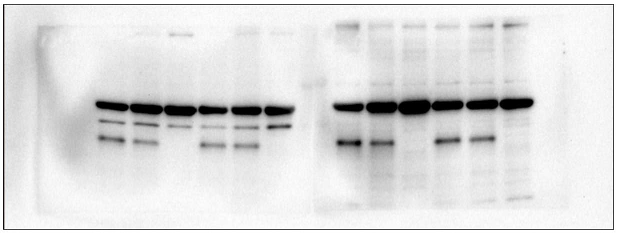

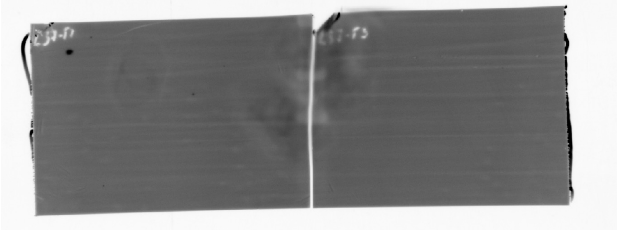

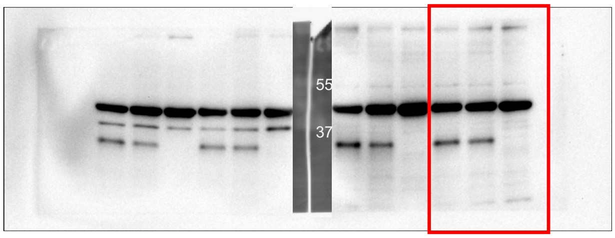

LIN37-T3 + β-actin

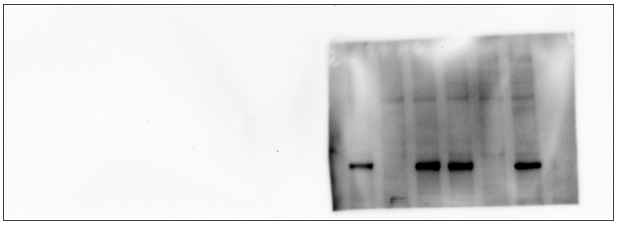

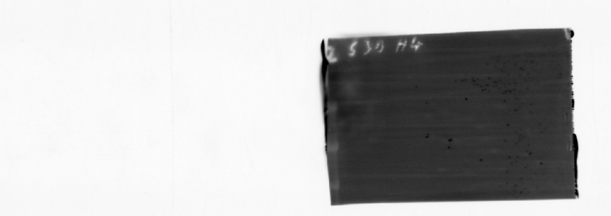

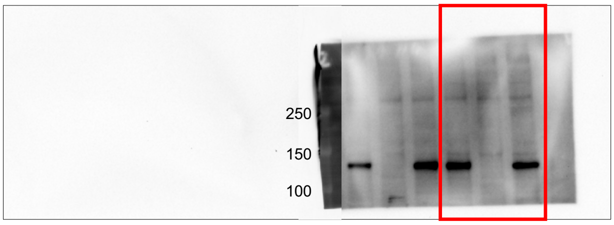

SIN3B-H4, SCBT

Fig. 5f

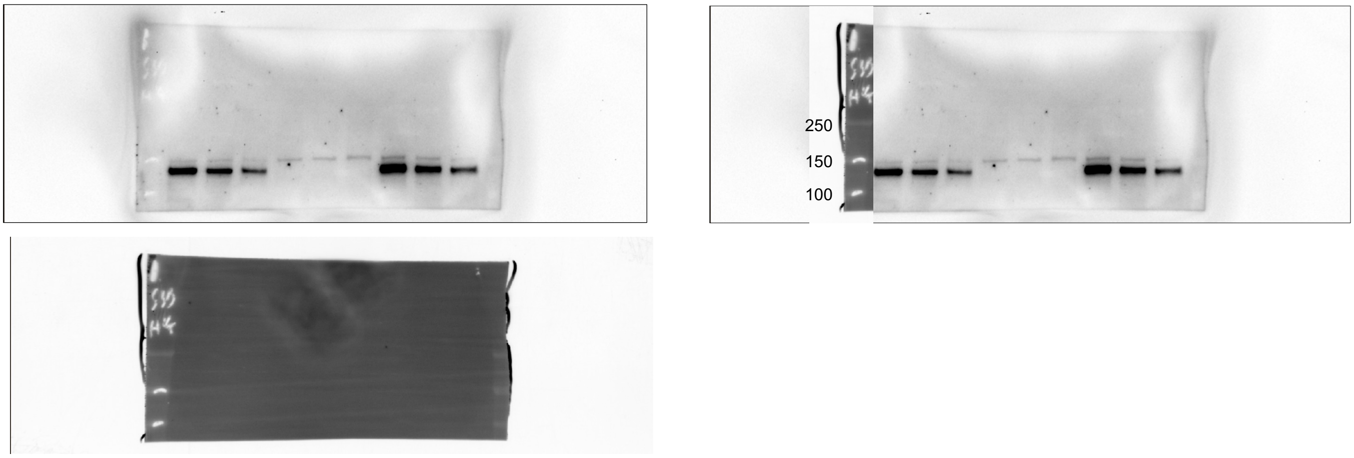

SIN3B-H4, SCBT

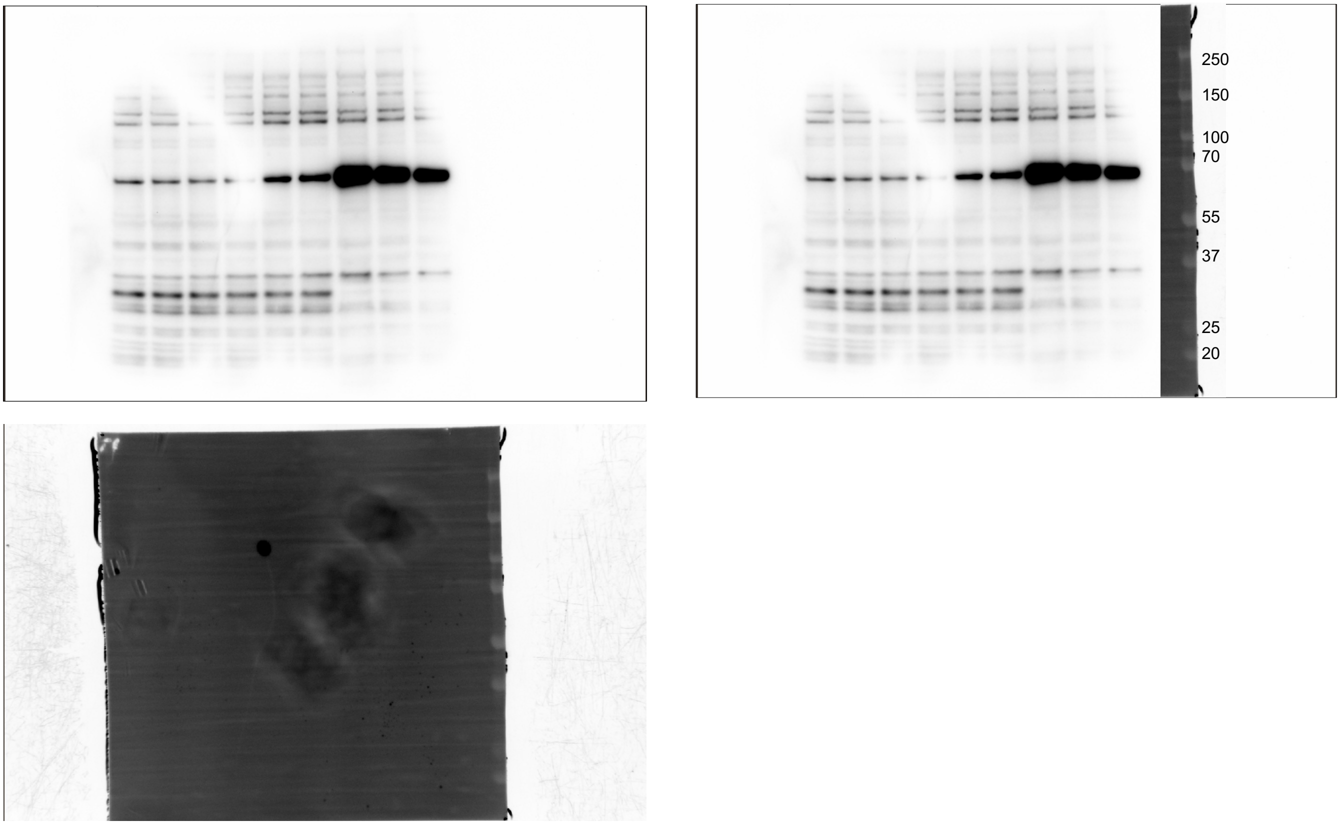

LIN37-T3

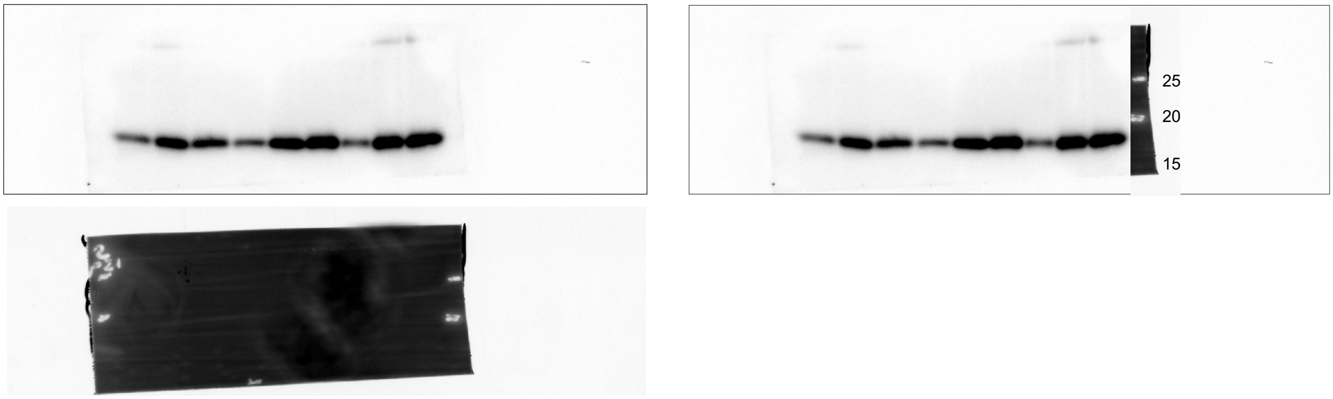

p21, SCBT

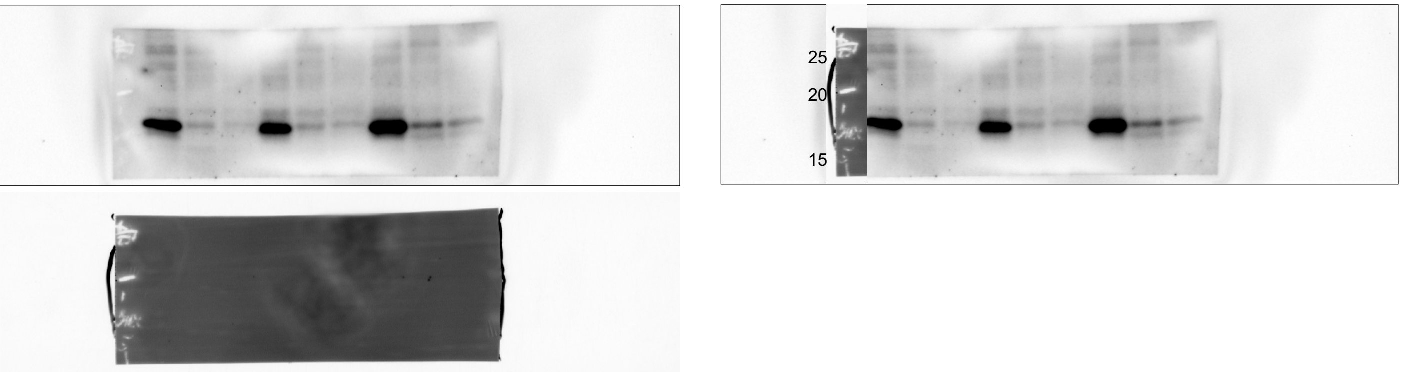

Survivin, SCBT

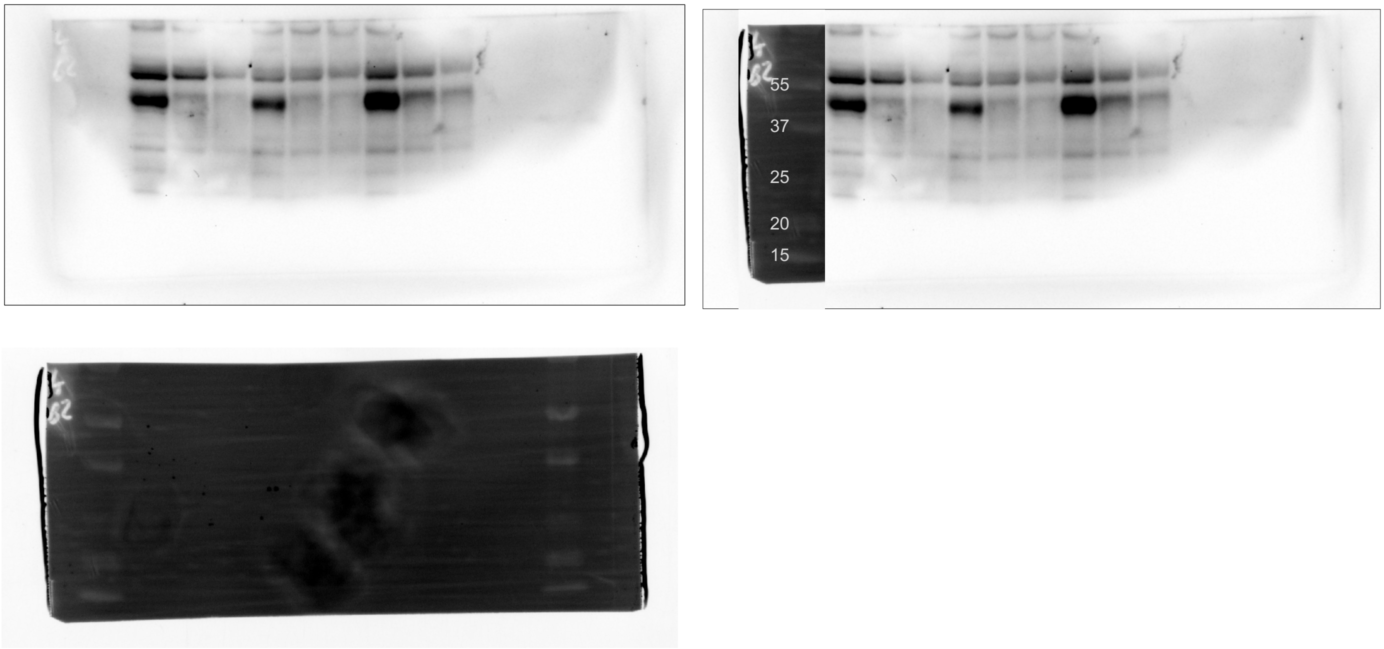

CCNB2, SCBT

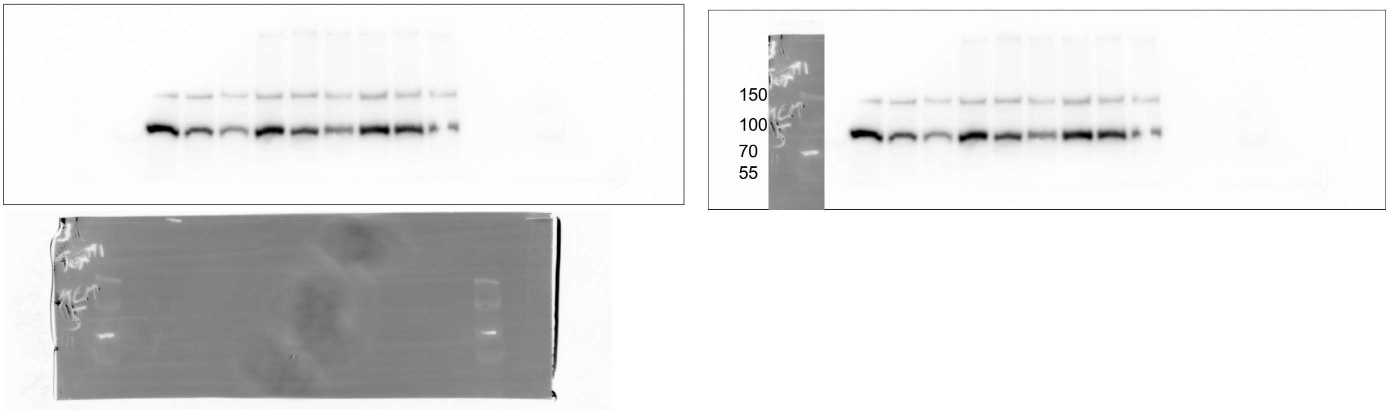

MCM5, SCBT

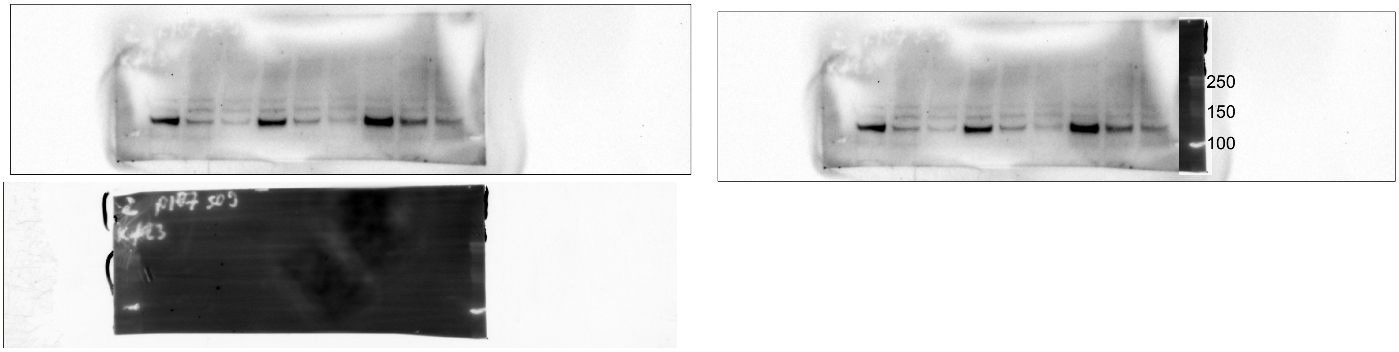

p107, SCBT

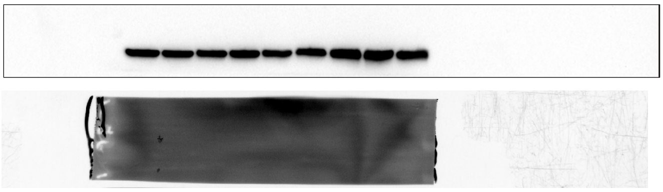

β-actin

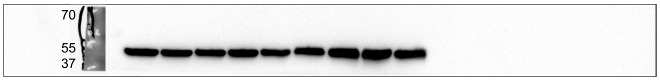

Fig. 6f

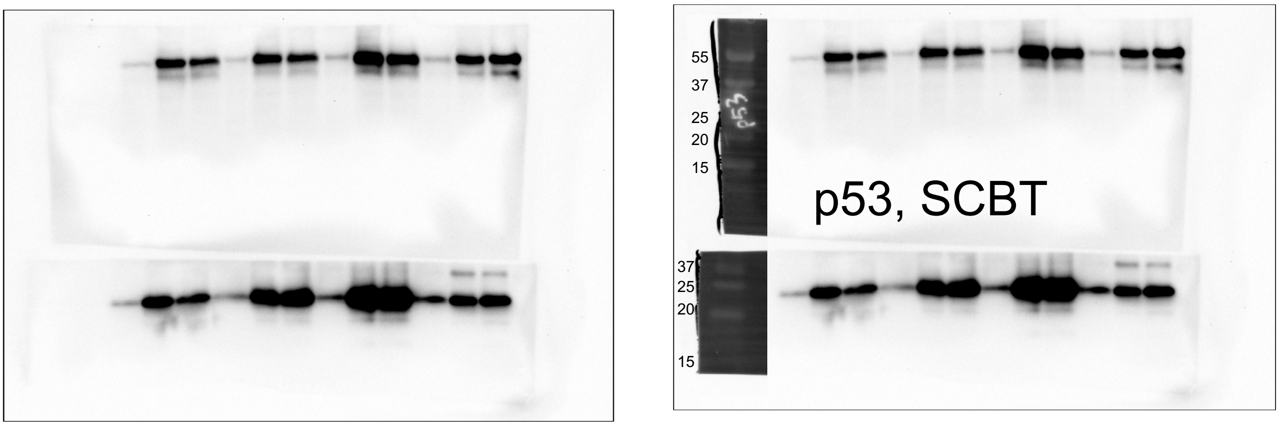

p21, SCBT

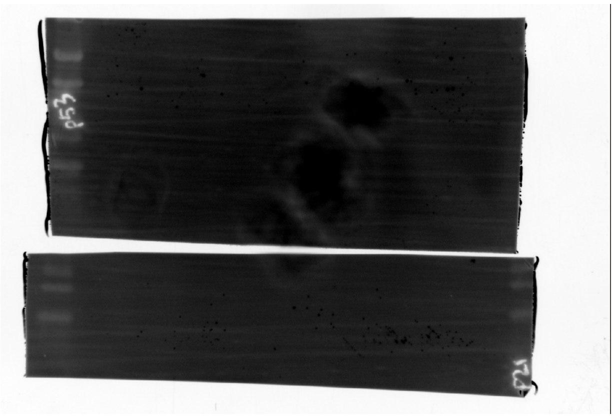

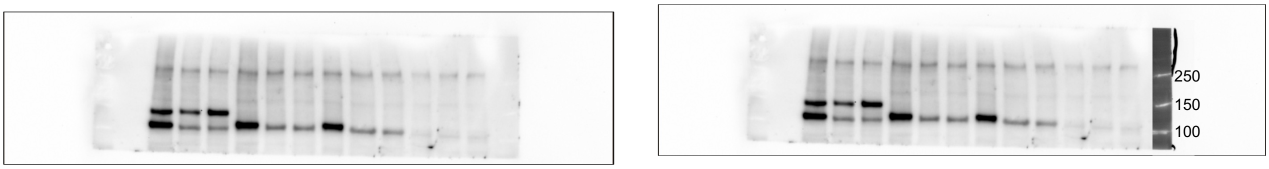

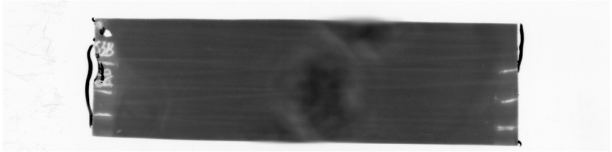

SIN3B polyclonas, Novus + RB-C2, SCBT

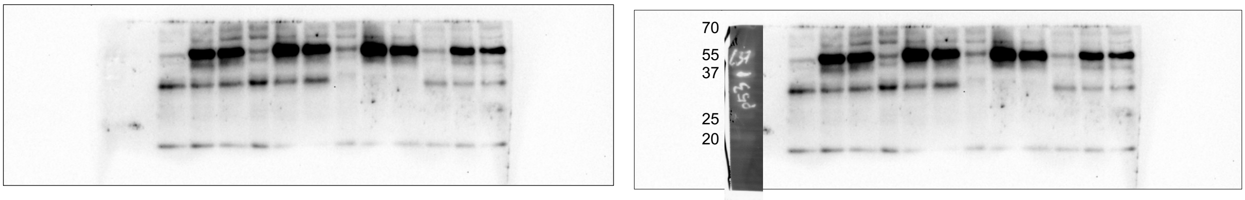

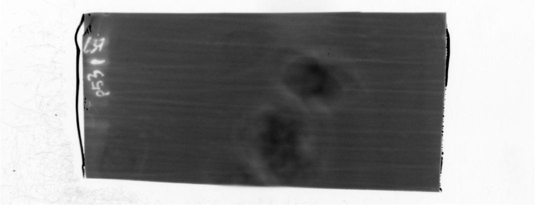

p53, SCBT + LIN37-T3

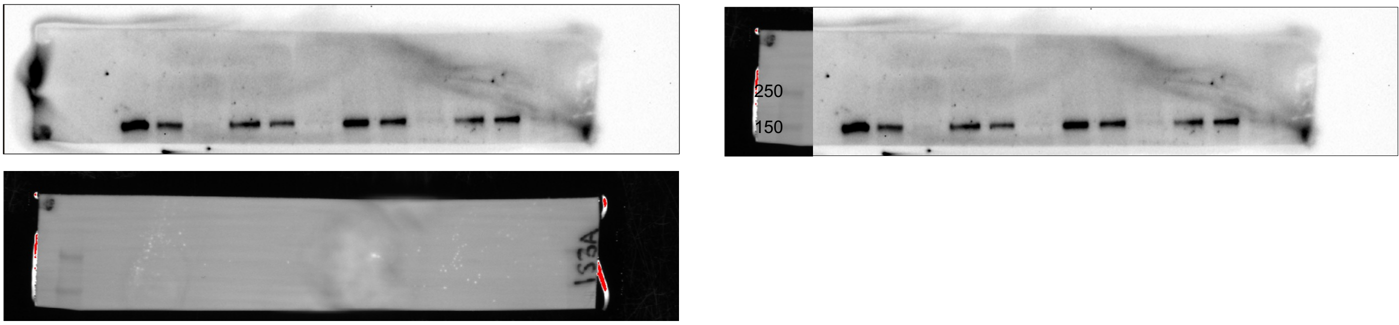

SIN3A-G11, SCBT

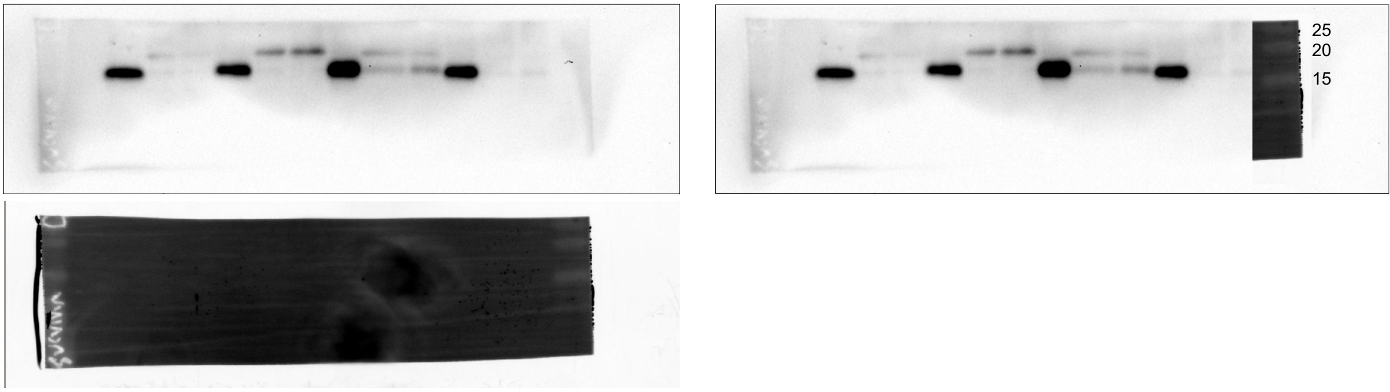

Survivin, SCBT

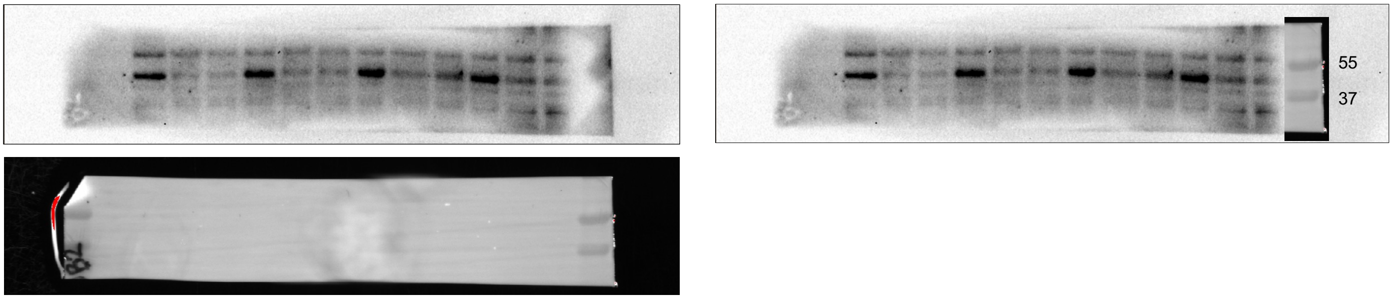

CCNB2, SCBT

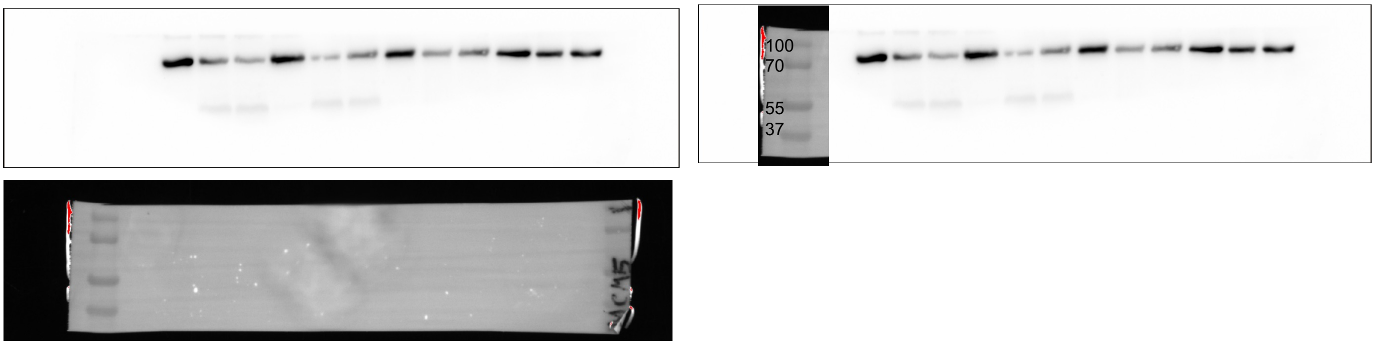

MCM5, SCBT

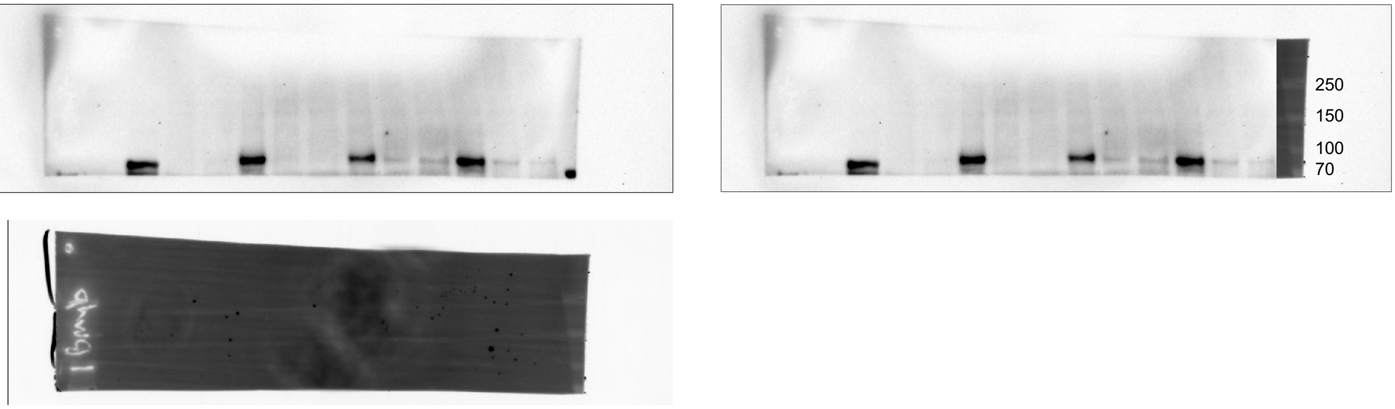

B-MYB, Millipore

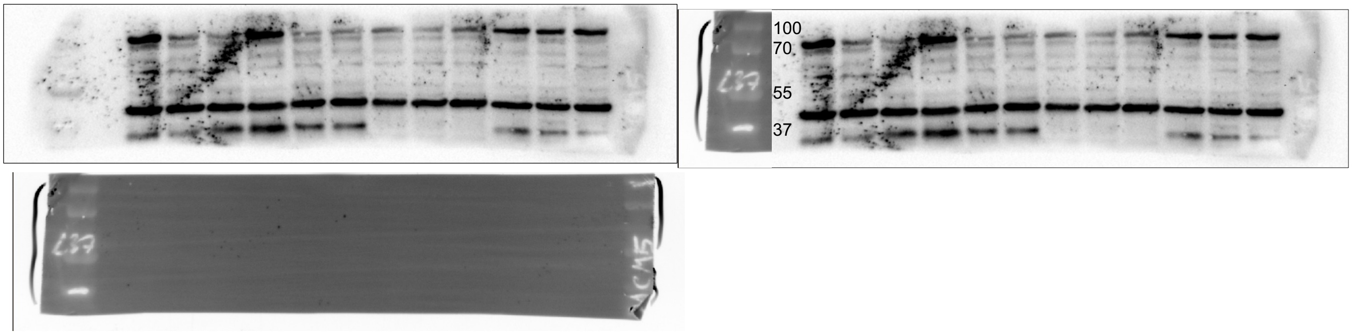

β-actin

Fig. 7c

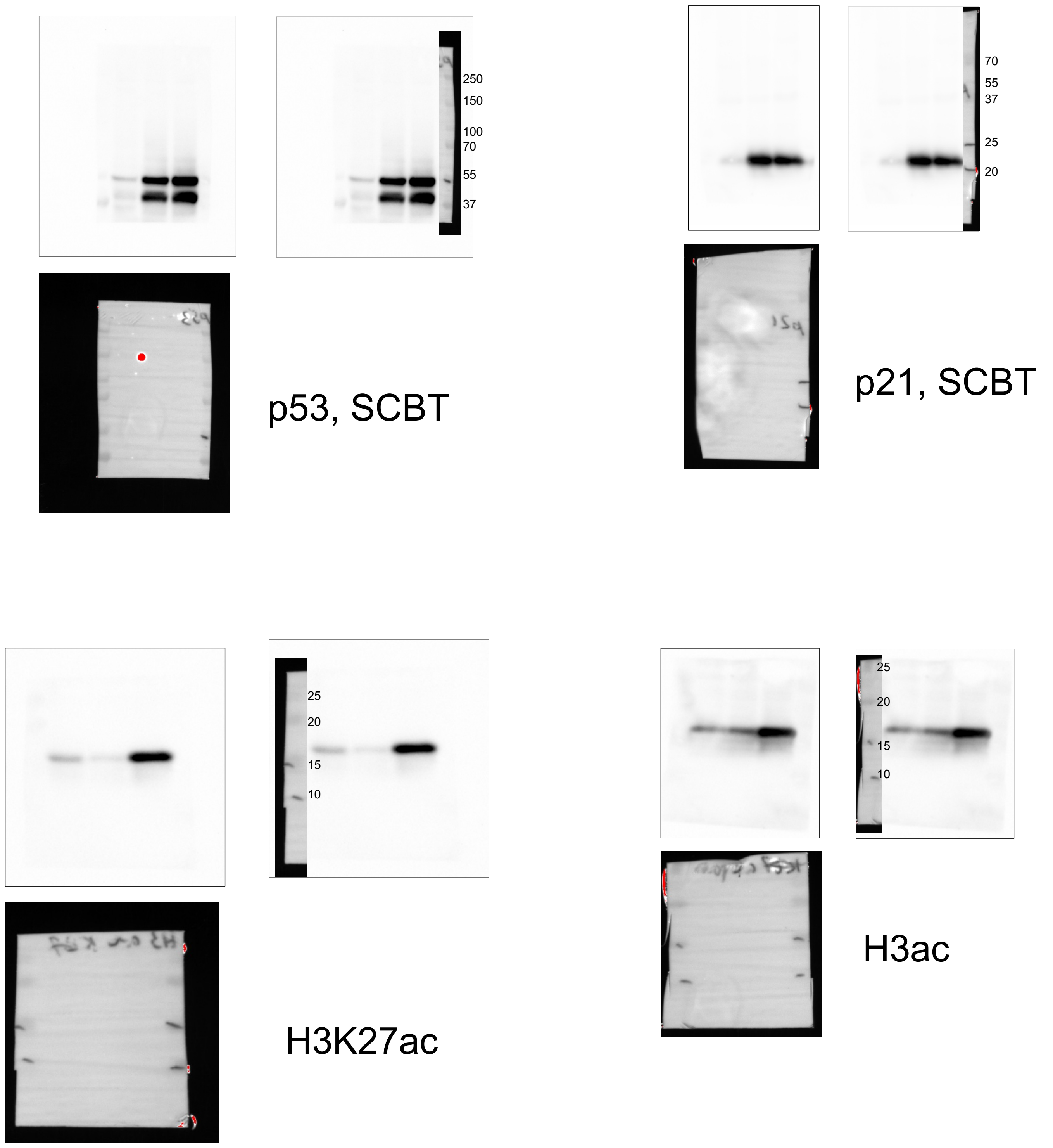
250
150
100
70
55
37
p53, SCBT
70
55
37
25
20
p21, SCBT
25
20
15
10
H3K27ac
25
20
15
10
H3ac

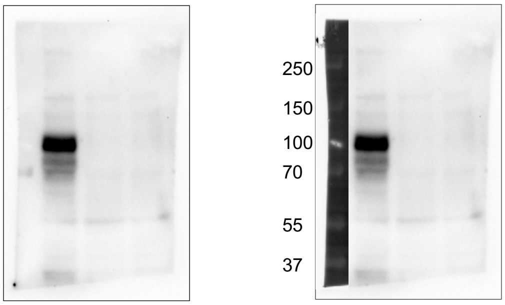

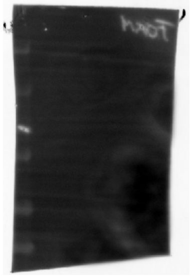

FOXM1, Cell Signaling

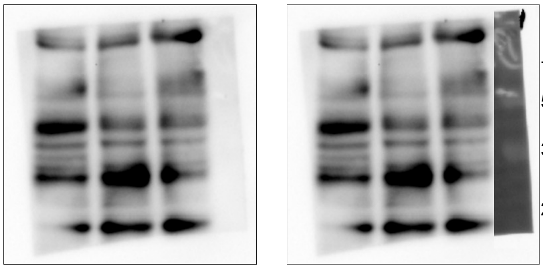

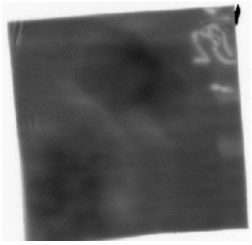

CCNB2, SCBT

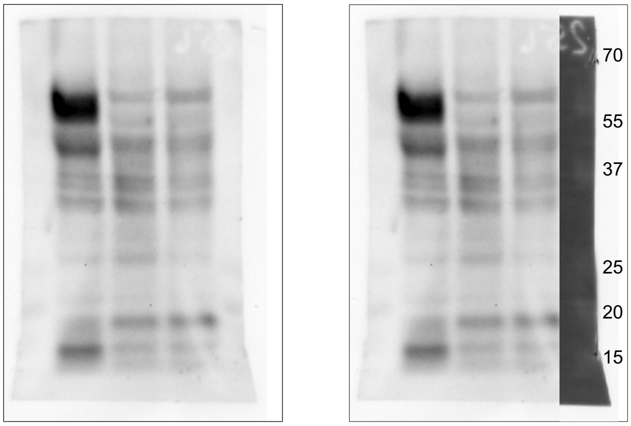

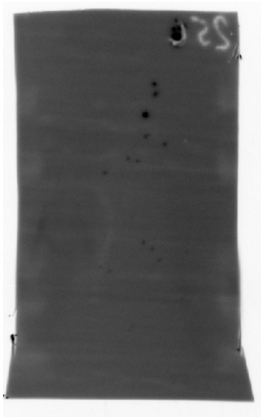

CDC25C, SCBT

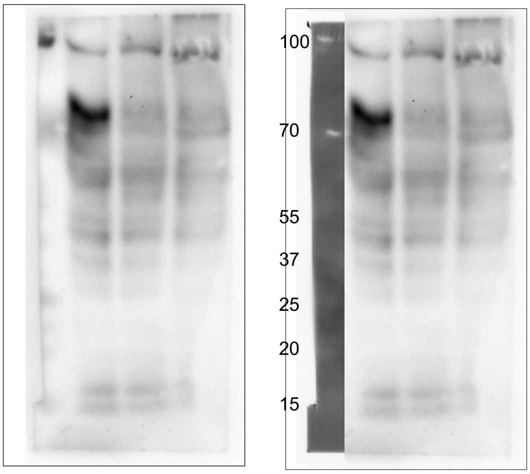

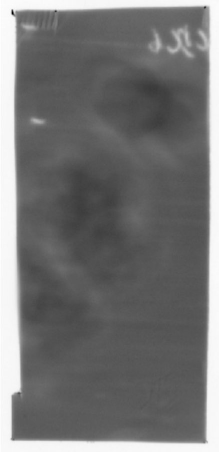

CDC6, SCBT

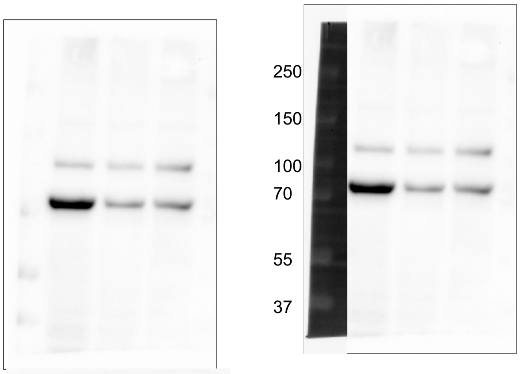

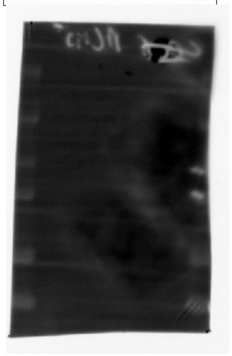

MCM5, SCBT

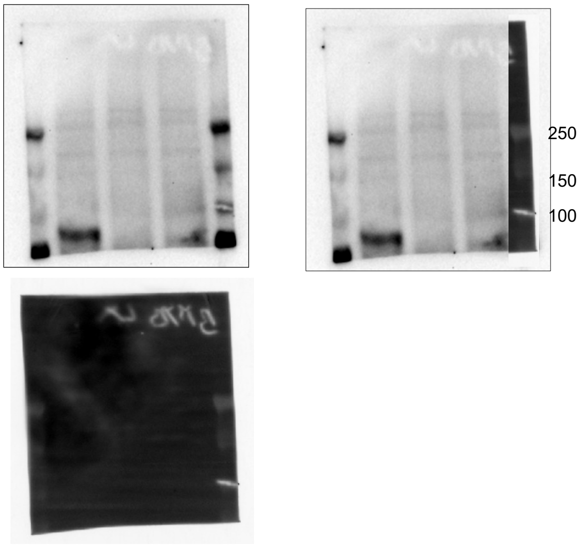

B-MYB, Millipore

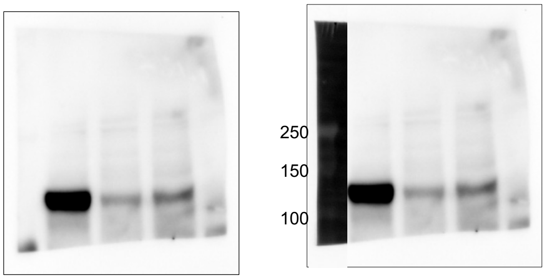

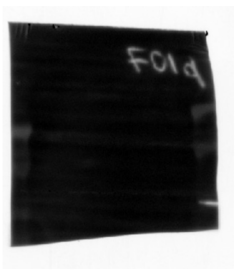

p107, SCBT

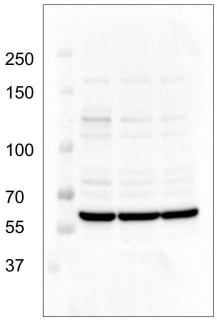

β-actin

Fig. 7e

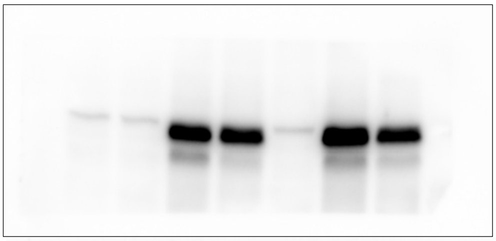

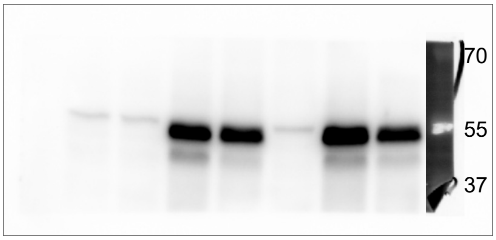

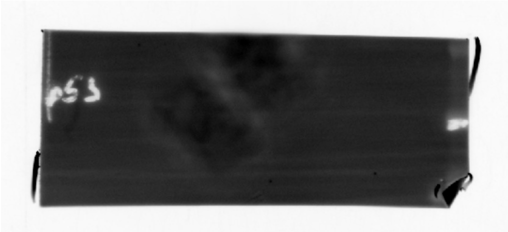

p53, SCBT

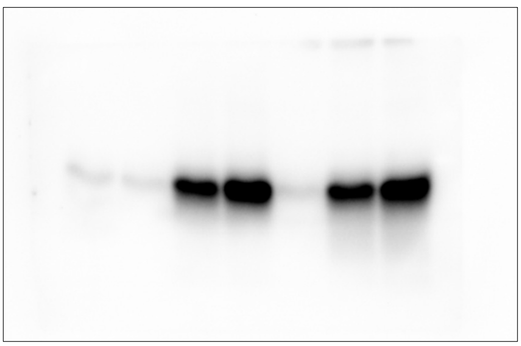

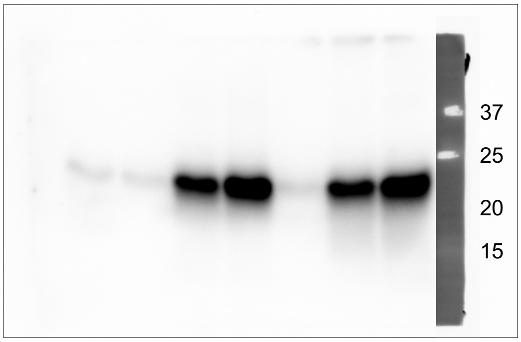

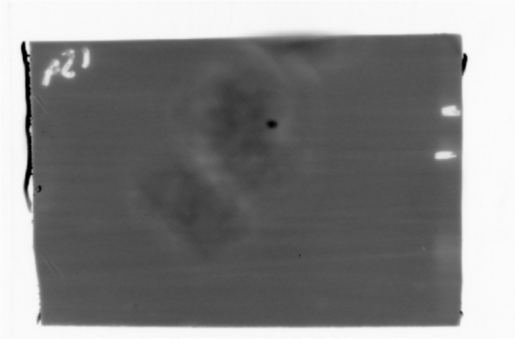

p21, SCBT

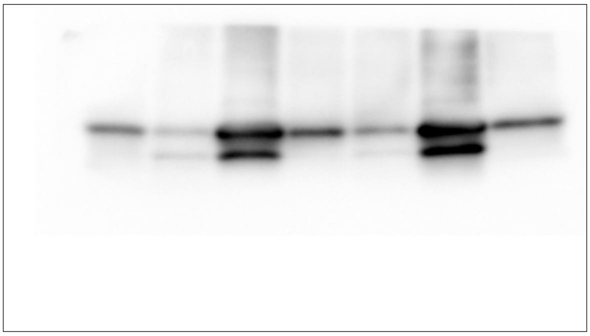

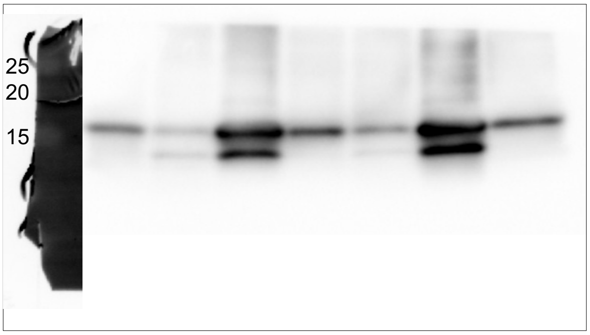

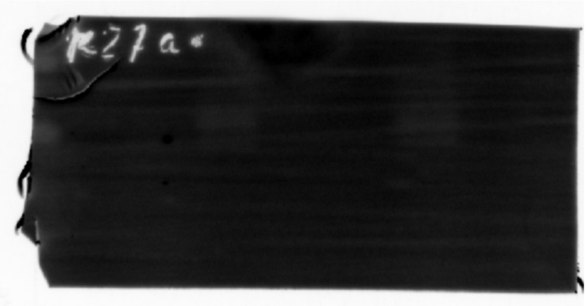

H3K27ac

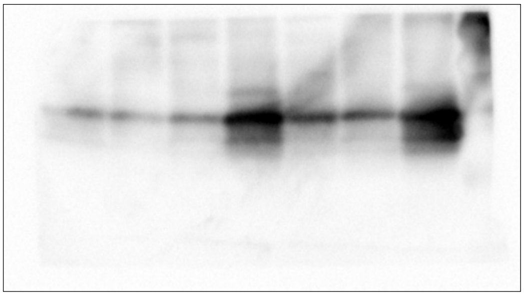

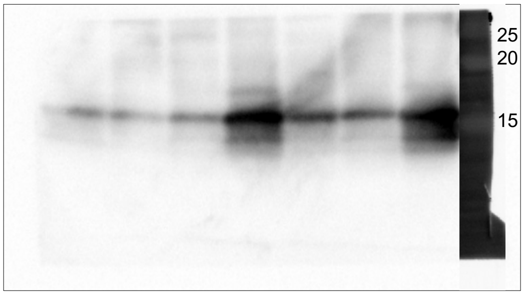

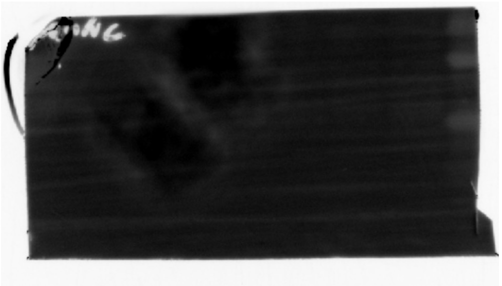

H3ac

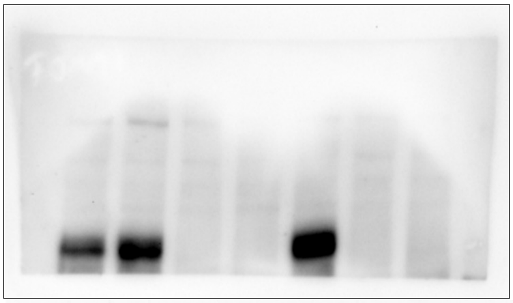

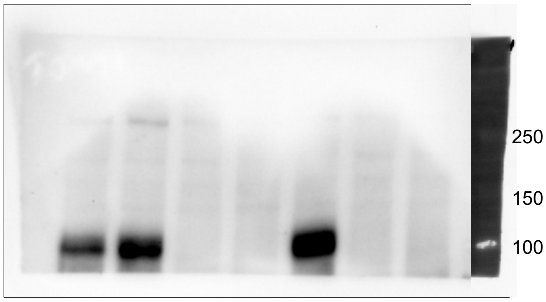

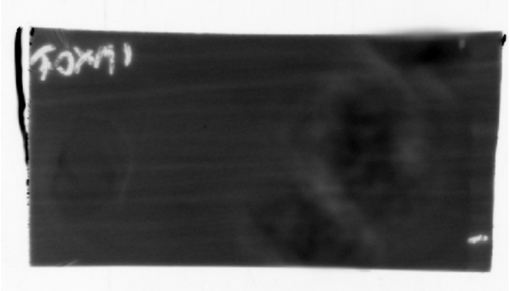

FOXM1

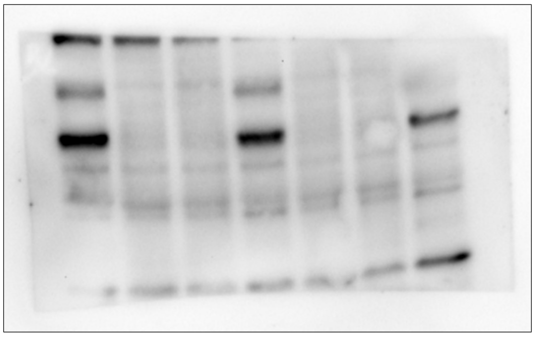

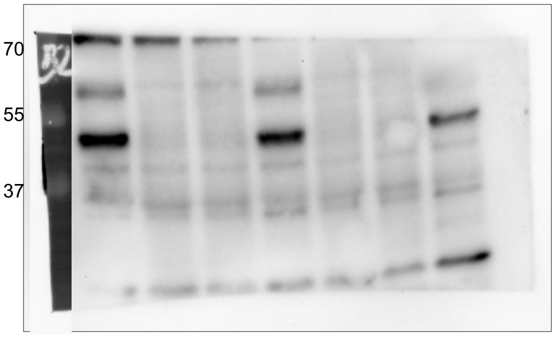

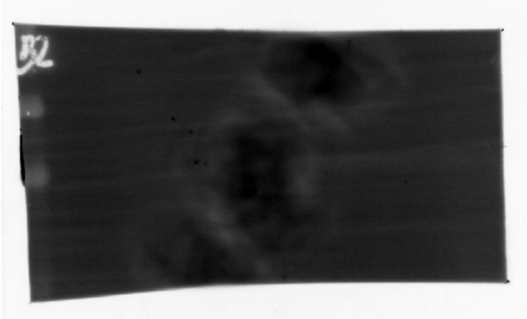

CCNB2

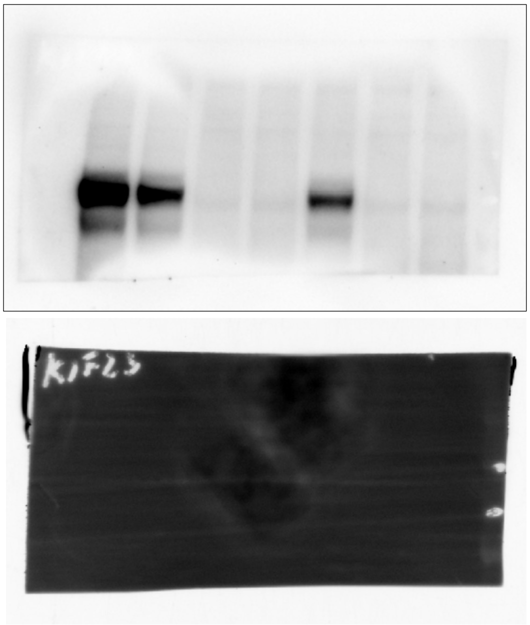

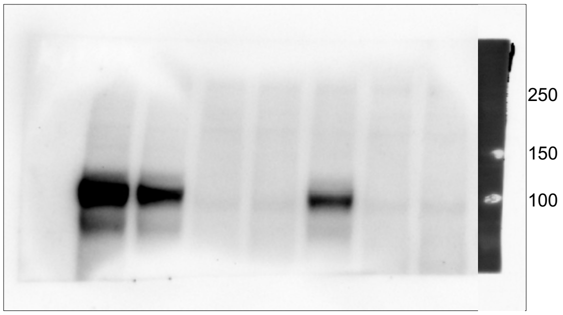

KIF23

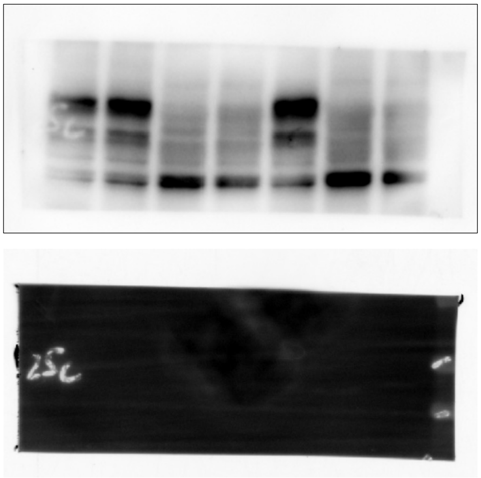

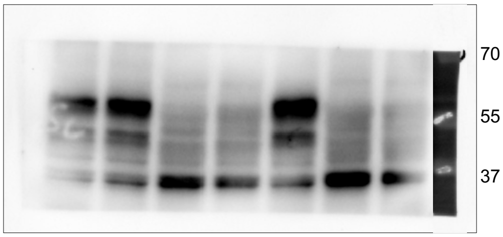

CDC25C

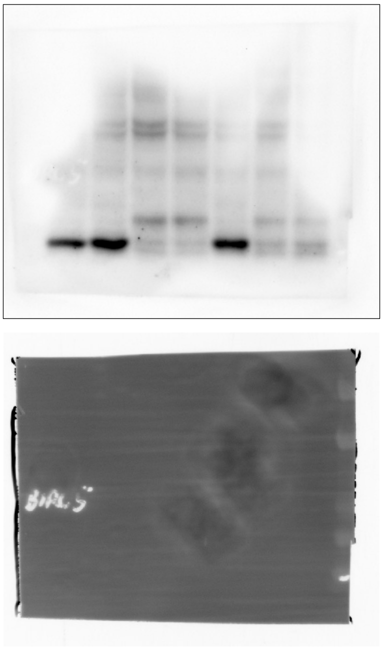

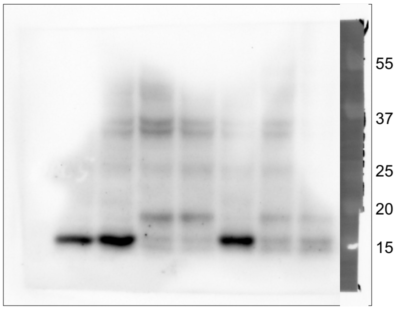

Survivin

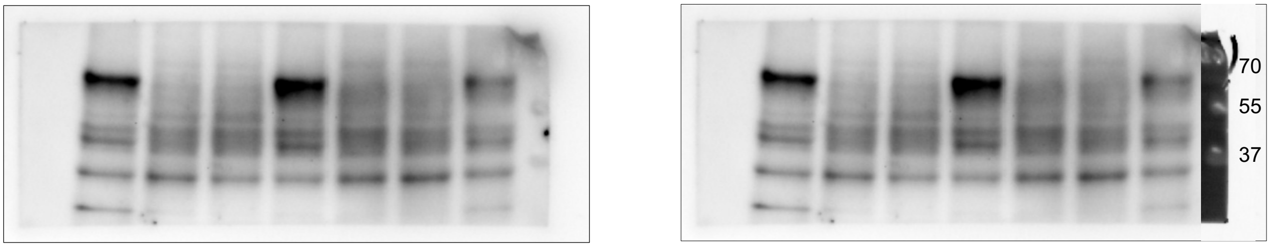

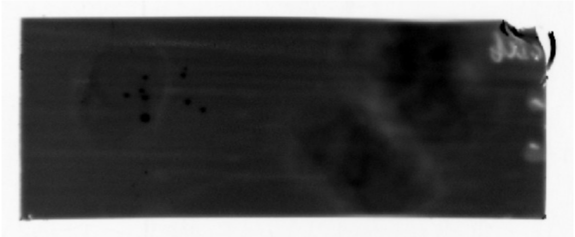

CDC6

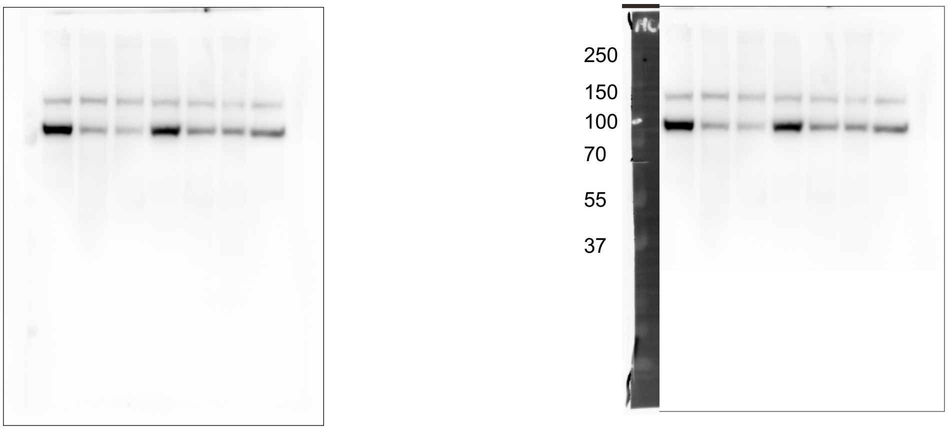

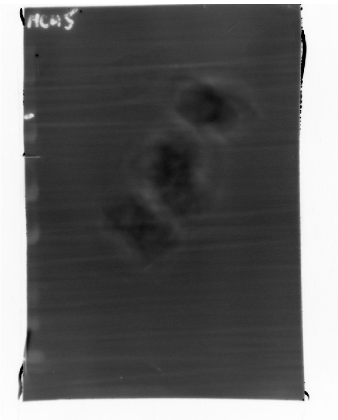

MCM5

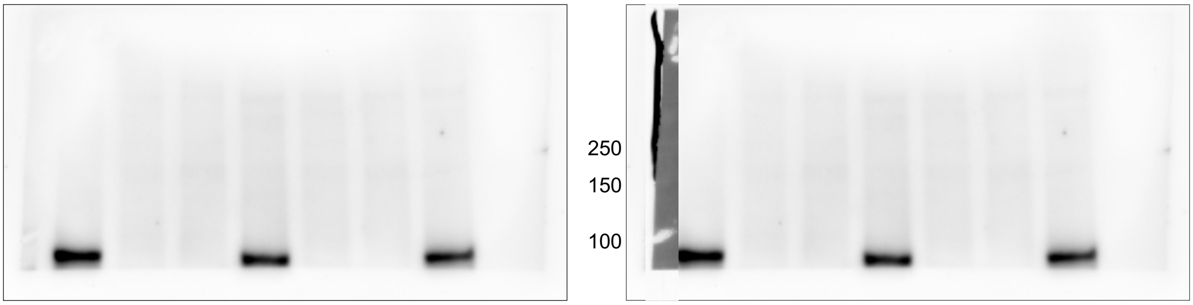

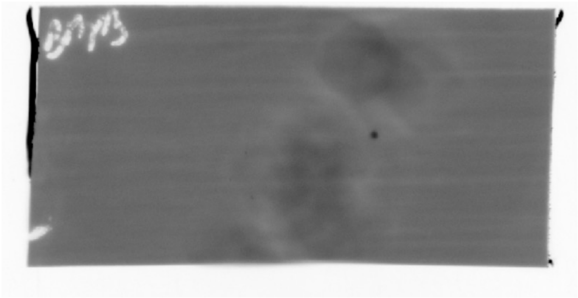

B-MYB

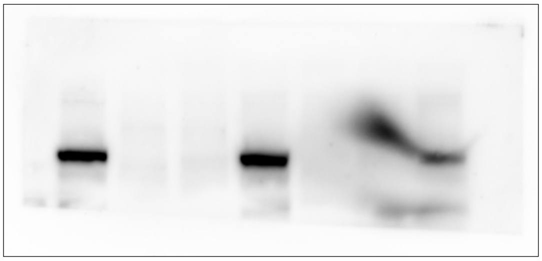

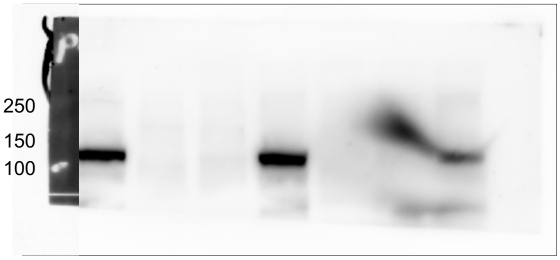

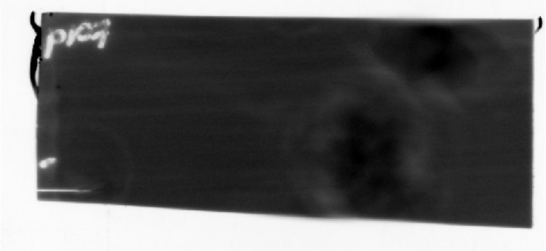

p107

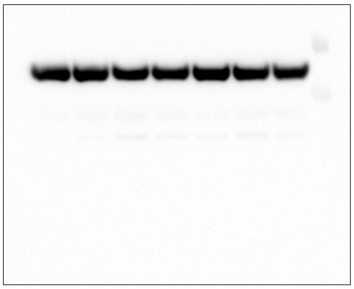

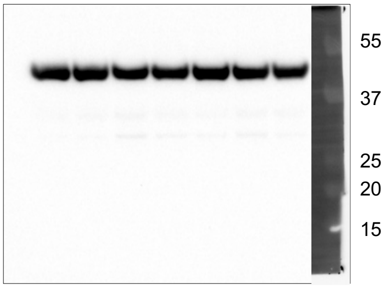

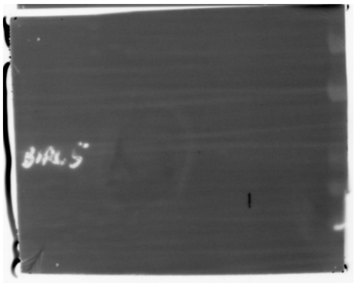

β-actin

Fig. 7h

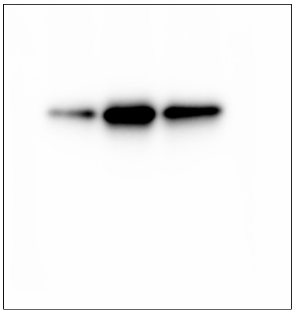

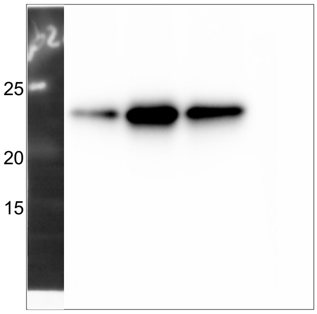

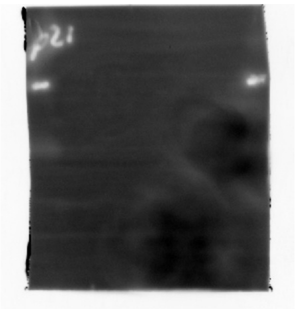

p21

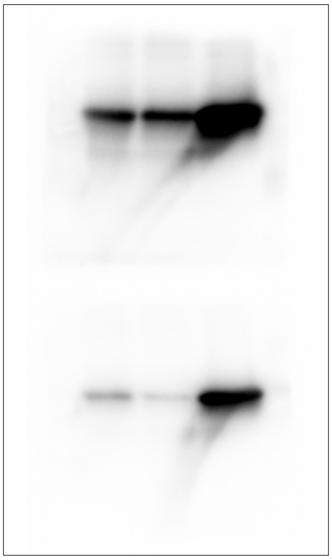

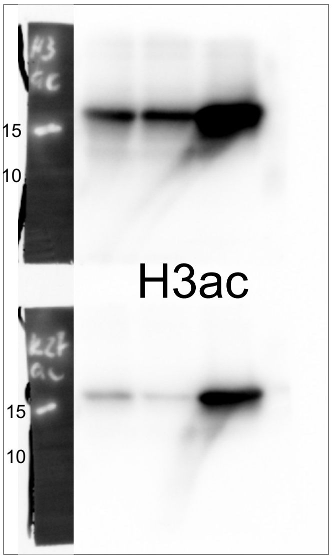

H3K27ac

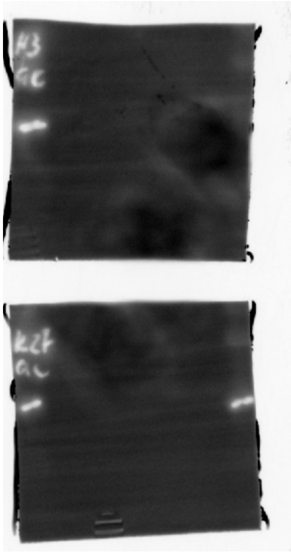

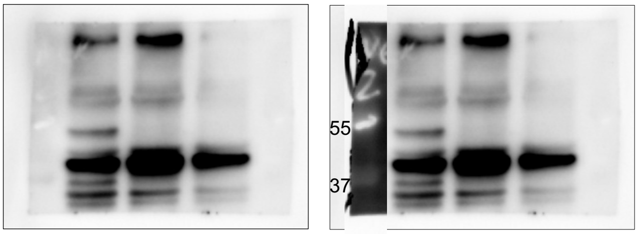

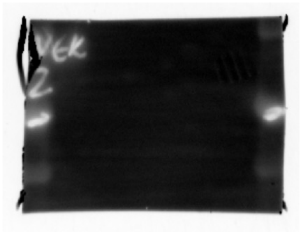

NEK2

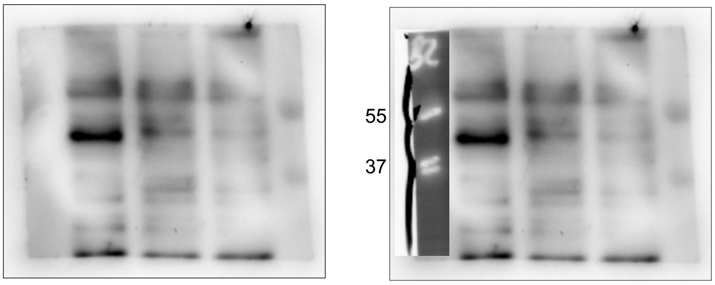

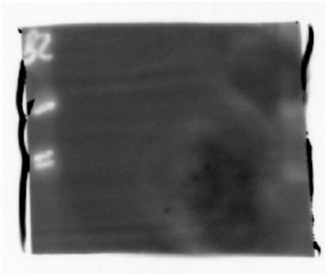

CCNB2

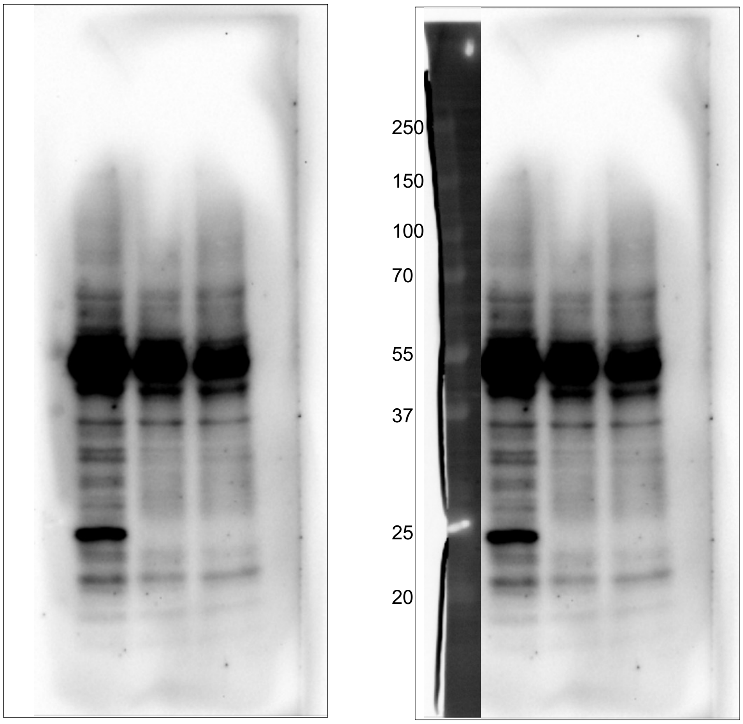

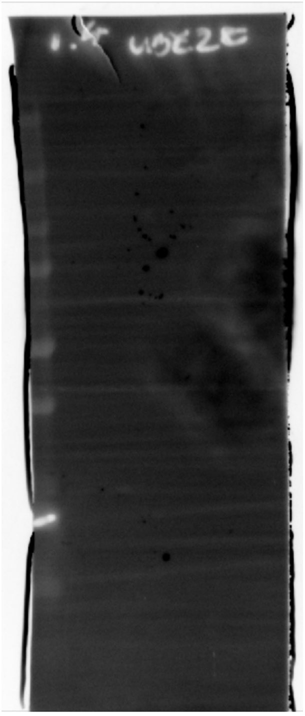

UBE2C

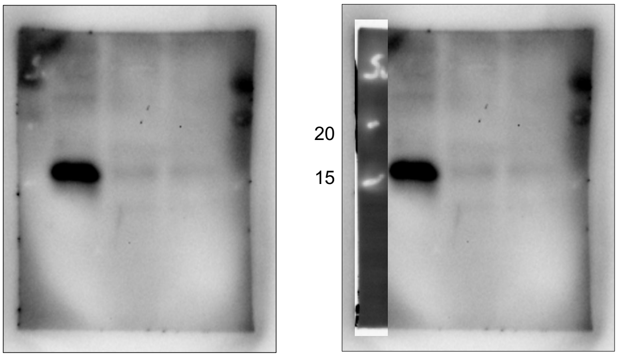

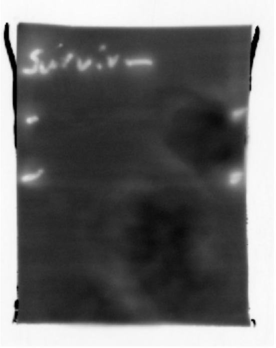

Survivin

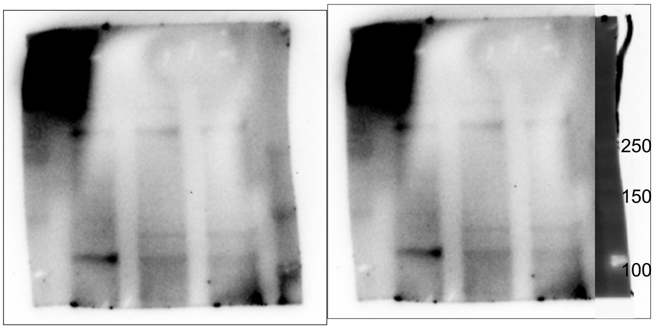

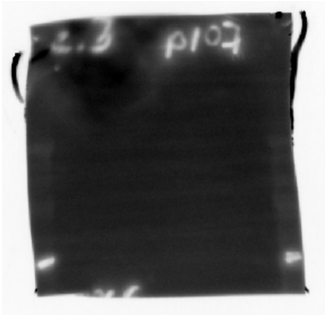

p107

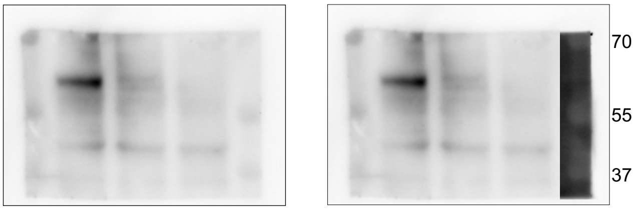

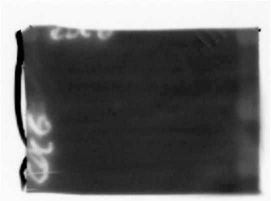

CDC6

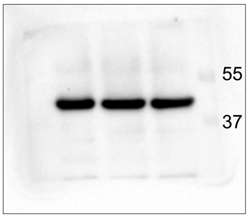

β-actin

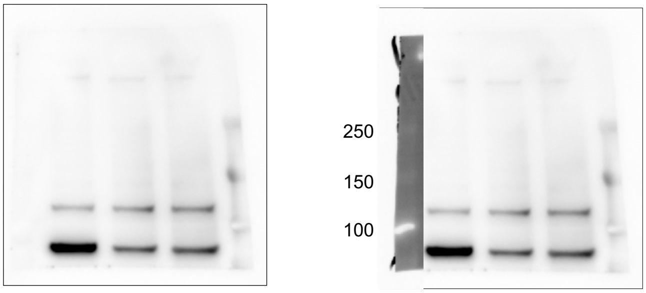

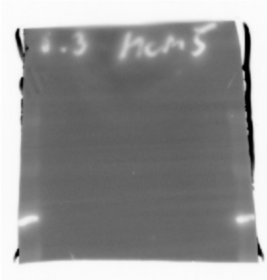

MCM5

Fig. 7i

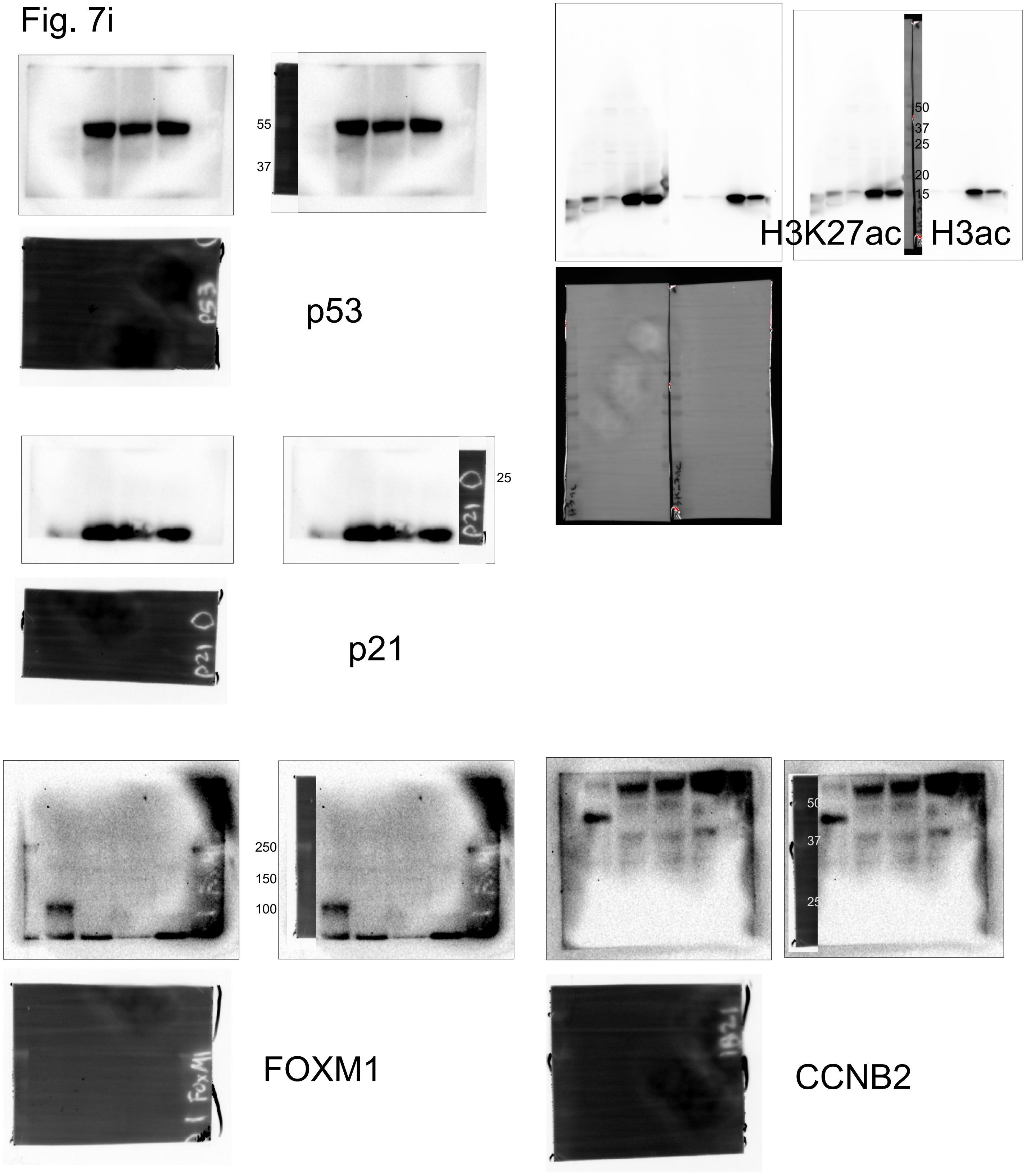

55
37
p53
25
p21
H3K27ac
H3ac
50
37
25
20
15
250
150
100
FOXM1
50
37
25
CCNB2

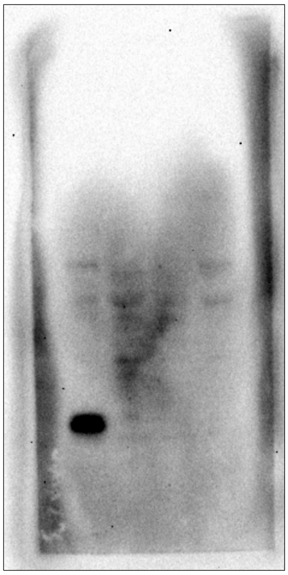

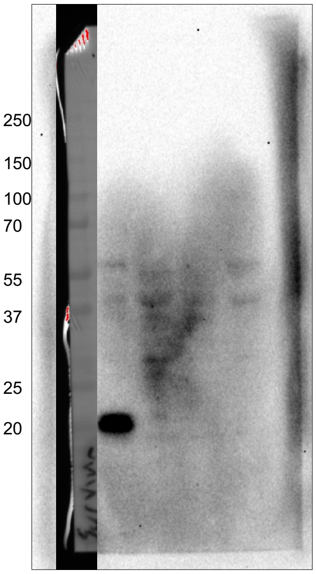

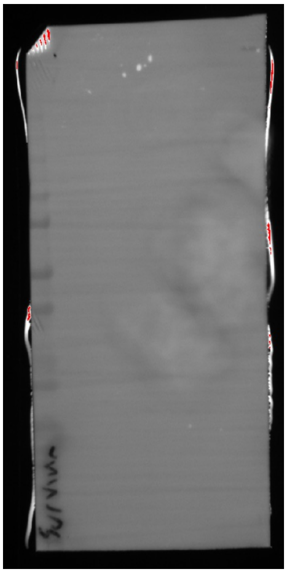

Survivin

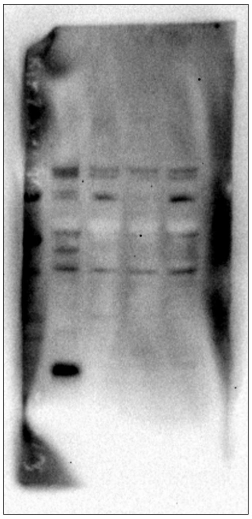

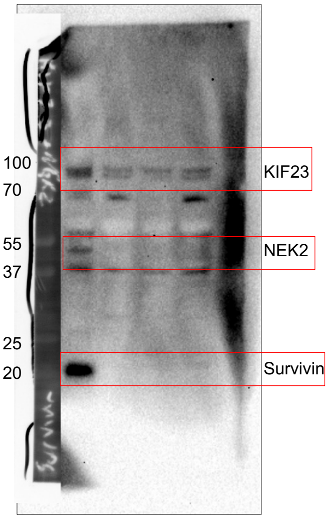

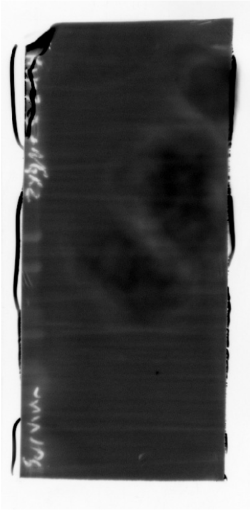

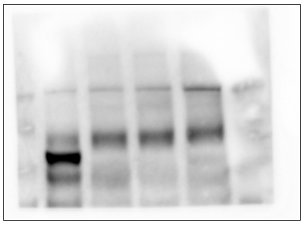

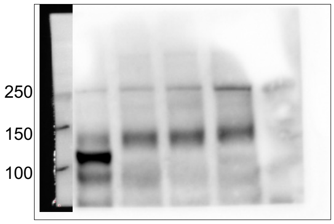

p107

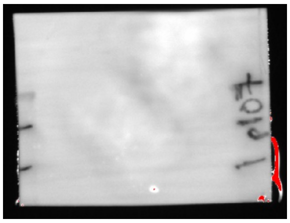

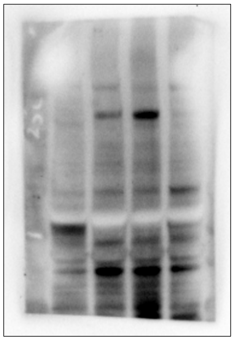

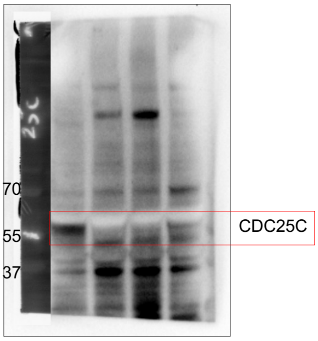

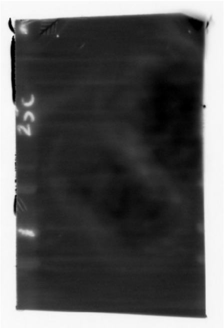

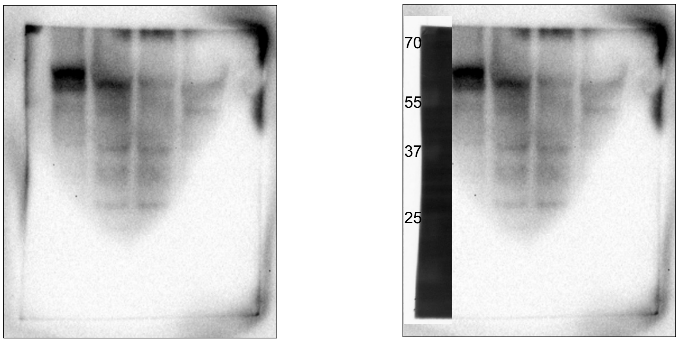

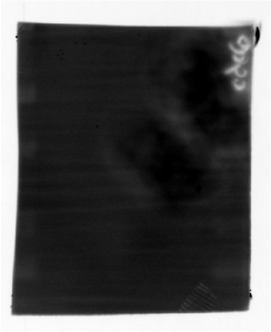

CDC6

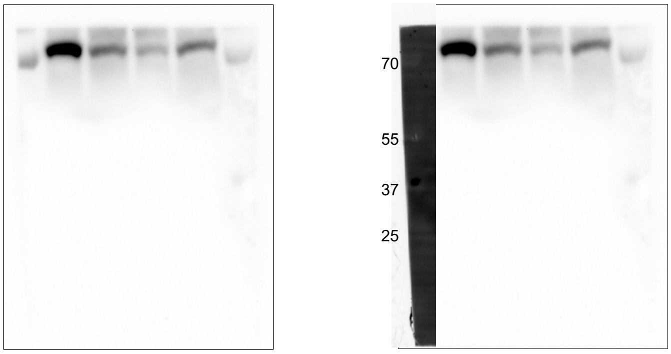

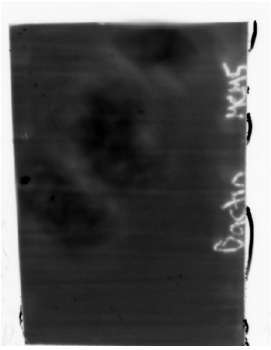

MCM5

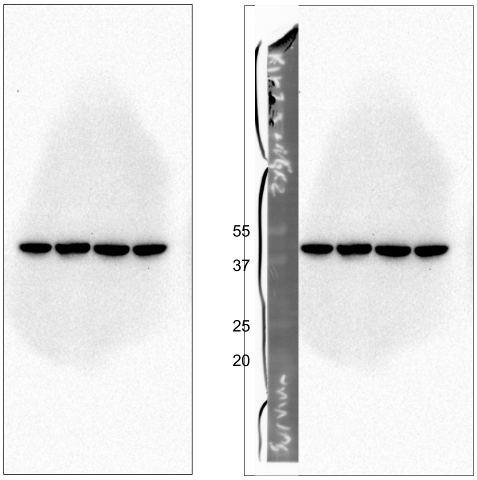

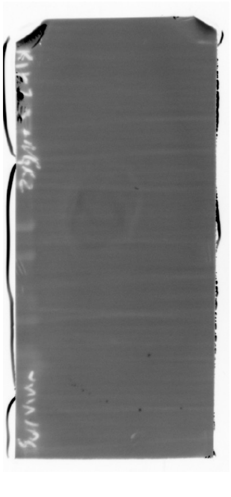

β-actin

Numerical values and p values of all experiments shown in the figures and supplementary figures

Fig. 2b

	WT					
BUB1	-4.4345348	-4.579621	-4.7255761	-4.658576	-4.3131862	-4.066
NEK2	-3.8758839	-4.230435	-4.2823381	-4.376887	-4.1737332	-4.250733
MCM5	-2.8028726	-2.838193	-3.1570389	-3.133039	-3.1244491	-3.068449
ORC1	-3.0855331	-3.381447	-4.0545008	-4.549501	-3.2745146	-3.308515

	SIN3B-/-											
BUB1	-4.246652	-4.01733	-5.219957	-5.313607	-5.355475	-5.448475	-5.2496451	-5.5286451	-4.9410055	-4.814	-5.136949	-5.248
NEK2	-4.83544	-4.952421	-6.201402	-6.621832	-5.640676	-5.870924	-6.6441485	-6.6262976	-4.9460281	-4.982028	-5.512844	-5.4558439
MCM5	-2.568727	-2.568727	-3.986149	-4.098616	-4.584917	-3.879917	-3.3458268	-3.9188268	-3.6793837	-3.671384	-3.948097	-4.0970968
ORC1	-4.317655	-4.362064	-5.75575	-5.848501	-5.136529	-4.919529	-6.1632717	-5.8182717	-5.1840419	-4.390042	-4.182531	-4.4305313

	T-Test / p values
BUB1	0.068438
NEK2	2.78E-06
MCM5	0.057524
ORC1	6.97E-05

	LIN37-/-					
BUB1	-1.0054506	-0.8224406	-1.5280188	-1.327019	-1.564792	-1.687
NEK2	-1.1953234	-1.1418089	-1.7884474	-1.814685	-2.015444	-1.893444
MCM5	-1.1202259	-1.0177639	-2.34869	-1.92969	-2.168126	-2.0411256
ORC1	-2.2602683	-2.0585719	-3.4380347	-3.506035	-3.170078	-3.353078

	SIN3B-/-;LIN37-/-											
BUB1	-1.2394825	-1.204533	-1.451673	-1.474345	-1.715984	-1.911984	-2.192798	-2.039798	-0.55181	-0.49	-0.606573	-0.547
NEK2	-0.9785082	-0.936493	-1.731886	-1.546307	-1.076041	-1.04814	-2.513822	-2.597531	-0.850126	-0.841126	-0.968014	-0.824014
MCM5	-1.2077726	-1.5086	-1.175331	-1.140446	-1.441892	-1.577892	-1.92374	-1.98574	-0.573217	-0.565217	-0.501725	-0.558725
ORC1	-1.8169789	-2.071717	-1.939387	-2.14746	-2.480003	-2.381003	-3.564611	-3.590611	-1.342035	-1.282035	-1.166675	-1.269675

	T-Test / p values
BUB1	0.585721
NEK2	0.10964
MCM5	0.120908
ORC1	0.023348

	RB-/-					
BUB1	-1.842301	-2.03598	-1.873126	-1.779126	-0.278675	-0.312
NEK2	-2.021825	-1.843867	-1.493299	-1.564596	0.075766	-0.102234
MCM5	0.853969	0.639101	0.941442	0.642442	0.918103	0.890103
ORC1	0.095549	0.268124	-0.499013	-0.352013	1.322718	1.293718

	SIN3B-/-; RB-/-											
BUB1	-1.266658	-1.304363	-2.322044	-2.545352	-1.721028	-1.531028	-1.840725	-1.840725	0.180292	0.2	-1.824186	-1.839
NEK2	-1.315963	-1.228976	-2.019521	-2.049327	-1.149391	-1.202596	-1.601918	-1.656048	-0.220291	-0.221291	-0.757153	-0.730153
MCM5	0.194594	0.201785	0.047092	-0.190437	-0.620175	-0.307175	0.799212	0.901212	0.781903	0.743903	0.204783	0.182783
ORC1	0.019546	0.080122	-0.373073	-0.259662	-0.134744	-0.192744	-0.653691	-0.621691	0.418751	0.377751	-0.229594	-0.299594

	T-Test / p values
BUB1	0.947708
NEK2	0.780297
MCM5	0.120039
ORC1	0.037346

	SIN3B-/-; RB-/-					
BUB1	-0.508936	-0.386272	-0.430148	-0.531148	-0.740962	-0.671
NEK2	-0.262586	-0.415898	-0.293724	-0.375674	-0.2281	-0.2091
MCM5	-0.174895	0.100435	-0.21952	-0.19852	-0.110796	-0.112796
ORC1	0.62948	0.354235	0.586612	0.602612	0.246	0.229

Fig. 2c

	WT							
BUB1	-6.0905761	-6.148576	-5.3922914	-5.092291	-5.9381862	-5.918186	-5.061292	-4.795292
NEK2	-7.8154935	-7.474889	-5.931	-6.365	-6.8917332	-6.658733	-4.948	-4.762
MCM5	-5.4500389	-5.082039	-4.616	-4.18	-4.6104491	-4.527449	-4.116	-4.033
ORC1	-6.4115008	-6.404501	-4.866	-5.12	-5.9765146	-5.802515	-4.571	-4.578

	SIN3B-/-											
BUB1	-6.274475	-6.115475	-6.077645	-5.638645	-5.55600555	-5.559005545	-5.2089486	-5.432949	-5.588528	-5.8955281	-5.6410632	-5.8590632
NEK2	-6.148357	-6.175624	-7.600655	-7.704613	-6.49402807	-6.933028072	-6.96984386	-6.334844	-6.975	-6.743	-5.148	-5.268
MCM5	-4.849917	-4.194917	-3.913827	-4.042827	-4.14638367	-4.117383669	-3.7830968	-3.819097	-4.519	-4.45	-4.661	-4.545
ORC1	-6.488529	-6.238529	-6.382272	-6.553272	-6.30404194	-6.302041935	-4.95353128	-4.702531	-6.321	-6.067	-5.504	-5.461

	T-Test / p values
BUB1	0.311943
NEK2	0.857962
MCM5	0.688726
ORC1	0.126268

	LIN37-/-							
BUB1	-2.0830188	-2.286019	-2.320792	-2.2007921	-2.1506002	-2.1996	-1.671589	-1.587589
NEK2	-2.7569246	-2.732802	-3.278444	-3.220444	-3.849	-3.965	-2.858	-3.075
MCM5	-2.82669	-2.54069	-2.164126	-2.1211256	-2.282	-2.778	-1.58	-1.484
ORC1	-3.7870347	-3.779035	-3.830078	-3.830078	-3.215	-3.405	-2.709	-2.904

	SIN3B-/-;LIN37-/-											
BUB1	-2.130984	-2.076984	-2.396798	-2.371798	-1.58181	-1.63081	-1.794573	-1.668573	-1.550415	-1.980415	-1.543449	-1.545449
NEK2	-2.585772	-2.704822	-2.704524	-2.809949	-2.521126	-2.658126	-2.613014	-2.423014	-2.722	-3.1	-2.653	-2.525
MCM5	-2.534892	-2.918892	-1.16774	-1.31774	-0.995217	-1.066217	-1.508725	-1.068725	-1.334	-1.292	-1.066	-0.928
ORC1	-3.870003	-4.318003	-3.300611	-3.420611	-2.833035	-2.823035	-2.842675	-2.611675	-3.379	-3.278	-2.834	-2.813

	T-Test / p values
BUB1	0.0209
NEK2	0.006399
MCM5	0.004528
ORC1	0.547106

	RB-/-					
BUB1	-4.349126	-4.337126	-3.675675	-3.735675	-5.288656	-5.087656
NEK2	-4.935404	-4.795942	-4.008234	-4.090234	-5.771	-5.99
MCM5	1.168442	1.311442	-0.050897	0.043103	-1.131	-1.036
ORC1	-1.609013	-1.688013	-1.180282	-1.304282	-3.216	-2.997

	SIN3B-/-; RB-/-											
BUB1	-3.850028	-3.889028	-4.753725	-4.671725	-3.006708	-3.225708	-4.560186	-4.827186	-4.788108	-5.059108	-4.948717	-4.909717
NEK2	-3.789352	-3.935203	-5.234202	-4.903489	-2.825291	-2.996291	-3.742153	-3.752153	-5.162	-5.565	-6.246	-5.872
MCM5	-0.410175	-0.553175	0.772212	1.488212	0.460903	0.483903	-0.581217	-0.706217	-0.596	-0.455	-1.515	-1.286
ORC1	-1.062744	-1.101744	-1.806691	-1.956691	-1.227249	-1.256249	-2.136594	-2.246594	-2.028	-2.206	-2.911	-3.055

	T-Test / p values
BUB1	0.756146
NEK2	0.162752
MCM5	0.703952
ORC1	0.304354

	SIN3B-/-; RB-/-					
BUB1	-1.343148	-1.392148	-1.266962	-1.522962	-1.517618	-1.372618
NEK2	-1.166065	-1.182585	-0.7471	-0.7561	-1.419	-1.789
MCM5	-1.57052	-0.86252	-0.952796	-1.160796	-0.471	-0.447
ORC1	-0.240388	-0.037388	-0.562	-0.85	-0.587	-0.899

Fig. 2e

	G0/G1				S				G2/M				T-Test / p values G0/G1	S	G2/M
WT	49.3	46.1	53.8	57.7	1.01	1.08	0.5	0.48	49.5	52.9	45.5	41.7			
SIN3B-/-	45.9	45.8	39.7	40.7	0.53	0.31	0.43	0.33	53.5	53.9	59.9	58.9	0.0282749	0.072205048	0.020831742
LIN37-/-	35.5	30.3	46.6	48.7	0.8	0.65	0.61	0.42	63.6	68.9	52.6	50.7	0.065458629	0.441209185	0.060195717
LIN37-/-;SIN3B-/-	37.4	36.6	47.9	48.9	1.63	0.9	0.67	0.72	61	62.4	51.4	50.3	0.073452579	0.468029715	0.067265826
RB-/-	37	33.8	30.8	31.2	1.2	0.95	0.71	0.68	61.5	65.2	68.5	68.1	0.000714118	0.580877029	0.000736872
RB-/-;SIN3B-/-	40.1	47.5	57.3	53.7	2.83	2.8	1.43	1.36	57.1	49.9	41.2	44.8	0.664304018	0.022939556	0.846921284
LIN37-/-;RB-/-	20.7	20.2	22.7	23	3.3	2.86	2.98	3.04	75.5	76.9	74.2	73.8	2.72569E-05	1.79436E-05	3.42753E-05

Fig. 2f

	G0/G1				S				G2/M				T-Test / p values G0/G1	S	G2/M
WT	56.2	58.5	53.6	57.8	0.57	0.66	0.56	0.49	43	40.7	45.7	41.7			
SIN3B-/-	51.6	55.3	54.5	55.7	1	0.94	0.52	0.43	47.4	43.8	44.9	43.8	0.166215984	0.344771525	0.160929969
LIN37-/-	35.8	32.6	53.7	59.1	1.21	1.2	0.63	0.57	62.7	66.1	45.6	40.3	0.141150662	0.111845859	0.140261542
LIN37-/-;SIN3B-/-	38.8	42.3	46.9	55.7	1.16	1.41	0.95	0.59	59.7	56.3	52	43.7	0.032041437	0.041489873	0.031028214
RB-/-	55.4	54.6	54.1	54.1	0.41	0.33	0.25	0.35	44	45	45.5	45.4	0.131036274	0.002734983	0.100817921
RB-/-;SIN3B-/-	67.1	71	64.1	62.4	0.65	0.35	0.57	0.78	32	28.6	35.3	36.6	0.004466101	0.862386769	0.00365907
LIN37-/-;RB-/-	39.7	40.3	43.3	44.4	0.52	0.63	0.38	0.31	59.6	58.8	56.1	55.1	8.93293E-05	0.216158163	7.30448E-05

Fig. 3a

	HCT116 WT ; IP: IgG			HCT116 WT ; IP: HDAC1			HCT116 WT ; IP: SIN3B			HCT116 WT ; IP: LIN37		
1x	3617	3948	4321	28200700	29430100	27439800	574194	573030	468270	27041	31023	27910
2x	4375	8625	4464	49381300	46501800	54376100	839949	813705	840489	35828	37960	35100
4x	4581	4493	5253	104498000	96590000	116281100	1012670	1051203	1036157	48030	49918	49931

	HCT116 WT ; IP: RBBP4			HCT116 SIN3B-/- ; IP: SIN3B			HCT116 SIN3B-/- ; IP: LIN37			HCT116 LIN37-/- ; IP: LIN37		
1x	2710329	2750801	2738068	5459	5386	4536	29155	32821	30931	28036	26117	26521
2x	3501476	3436706	3295577	8824	7868	6555	41183	43873	44224	40008	38348	38433
4x	3405425	3394652	3326879	9119	8225	8025	56007	57694	55930	49190	48982	49592

Fig. 3c

[illegible]

Fig. 3e

[illegible]

Fig. 3f

[illegible]

Fig. 4b

[illegible]

Fig. 4d

[illegible]

Fig. 4f

[illegible]

Fig. 4h

[illegible]

Fig. 4i

[illegible]

Fig. 5b

[illegible]

Fig. 5c

[illegible]

Fig. 5d

[illegible]

Fig. 5e

[illegible]

Fig. 6d

[illegible]

Fig. 6e

[illegible]

SIN3A siRNA-4	0.004939694	0.88421	0.006331511

Fig. 6g

WT - siCTRL

SIN3A	-1.00074637	-0.879746	-1.38987511	-1.435875	-1.3811602	-1.31716
CENPW	-2.7873318	-2.143332	-3.0462433	-3.123243	-4.0127174	-4.671717
SGO2	-4.354349	-4.438349	-4.594771	-4.752771	-4.679814	-4.468814
HJURP	-6.5936216	-6.173622	-6.1752453	-6.301245	-6.4870008	-6.223001
CDCA5	-4.5835542	-4.352554	-4.7508105	-4.558811	-4.4525855	-4.829586
KIF20A	-4.1990499	-3.93105	-2.7346259	-2.733626	-2.7386547	-2.448655
CENPE	-3.6445001	-3.7215	-3.8513044	-4.164304	-3.4141602	-3.70016
MCM5	-5.23823218	-4.786232	-4.11669868	-3.988699	-4.01040125	-4.242401

WT - SIN3A si1

SIN3A	-1.290246	-1.447246	-2.003875	-2.058875	-1.27416	-1.19016	0.089254	ns
CENPW	-2.830832	-2.521832	-2.129243	-1.898243	-4.820717	-4.106717	0.389268	ns
SGO2	-3.667849	-3.459849	-3.954771	-3.924771	-4.542814	-4.918814	0.060754	ns
HJURP	-6.731122	-6.933122	-6.559245	-6.732245	-6.624001	-6.818001	0.008491	**
CDCA5	-5.193054	-4.776054	-4.599811	-4.130811	-4.453586	-4.892586	0.811802	ns
KIF20A	-4.00055	-3.61055	-3.522626	-3.277626	-3.450655	-3.540655	0.077073	ns
CENPE	-3.192	-3.413	-3.590304	-3.890304	-3.97216	-4.30516	0.829624	ns
MCM5	-4.037732	-3.936732	-3.768699	-3.894699	-4.118401	-4.167401	0.084117	ns

WT - SIN3A si4

SIN3A	-2.2802464	-1.9692464	-2.3528751	-2.357875	-2.72016	-2.2751602	0.000233013	***
CENPW	-3.0788318	-3.3038318	-2.6662433	-2.144243	-5.176717	-4.5517174	0.833397761	ns
SGO2	-3.424849	-3.340849	-3.891771	-3.662771	-5.130814	-4.807814	0.08633274	ns
HJURP	-6.4131216	-6.2991216	-7.0342453	-6.697245	-7.039001	-6.9800008	0.01529388	*
CDCA5	-5.2550542	-5.0230542	-4.9988105	-4.676811	-5.393586	-5.7015855	0.021101029	*
KIF20A	-4.6885499	-4.2445499	-3.6696259	-3.996626	-4.220655	-4.0356547	0.008178903	**
CENPE	-2.8680001	-2.7810001	-3.3123044	-3.878304	-4.20716	-3.8311602	0.263790981	ns
MCM5	-4.3587322	-4.4457322	-4.0916987	-4.211699	-4.354401	-4.6424013	0.927953678	ns

SIN3B-/- - siCTRL

SIN3A	-1.8708367	-1.6978367	-1.4135055	-1.352506	-0.690509	-0.6025087
CENPW	-2.3212266	-2.3942266	-2.0652509	-2.424251	-3.282501	-3.5435009
SGO2	-4.4380273	-4.5150273	-5.3126259	-5.537626	-4.412073	-4.0120729
HJURP	-6.7006734	-6.8046734	-7.0772447	-6.856245	-6.310119	-6.8991186
CDCA5	-5.5052984	-6.0702984	-4.663833	-4.315833	-4.891054	-4.6710542
KIF20A	-3.1100008	-3.0920008	-2.5283724	-2.686372	-1.70053	-1.9065303
CENPE	-4.8152017	-5.4242017	-4.3645136	-4.467514	-3.379652	-3.4436518
MCM5	-5.2515679	-5.0905679	-4.2380542	-4.123054	-3.589322	-3.6123224

SIN3B-/- - SIN3A si1

SIN3A	-2.1518367	-2.275837	-2.046006	-2.127006	-1.644009	-1.387009	0.046751	*
CENPW	0.34177339	-0.225227	0.197249	0.388249	-1.286001	-1.025001	0.000542	***
SGO2	-3.3910273	-3.347027	-3.378126	-3.409126	-2.960573	-2.805573	0.000744	***
HJURP	-5.9856734	-5.634673	-5.007745	-5.060745	-5.500619	-5.537619	0.000293	***
CDCA5	-4.3142984	-4.456298	-3.103333	-3.079333	-3.874554	-4.018554	0.016027	*
KIF20A	-1.8340008	-1.902001	-1.078872	-1.075872	-0.97903	-0.74403	0.009629	**
CENPE	-3.2782017	-3.435202	-2.695014	-2.482014	-3.672152	-3.379152	0.010983	*
MCM5	-3.4555679	-3.490568	-2.693554	-2.574554	-2.836822	-3.203822	0.001928	**

SIN3B-/- - SIN3A si4

SIN3A	-2.313337	-2.639337	-2.478506	-2.544506	-2.053009	-2.325009	0.005695	**
CENPW	-0.080727	-0.030727	0.381749	0.426749	-1.581001	-1.503001	0.001475	**
SGO2	-3.023527	-2.823527	-1.766626	-1.554626	-2.776573	-2.985573	0.00491	**
HJURP	-5.946173	-5.927173	-5.540245	-5.082245	-5.488619	-5.676619	0.000309	***
CDCA5	-4.558798	-4.455798	-3.559833	-3.591833	-4.066554	-4.321554	0.019235	*
KIF20A	-1.667501	-2.191501	-1.915372	-1.842372	-1.32203	-1.02903	0.066633	ns
CENPE	-2.917702	-3.017702	-2.824514	-2.698514	-2.614152	-2.880152	3.81E-05	***
MCM5	-3.390068	-3.398068	-2.989054	-3.056054	-3.045822	-3.173822	0.000459	***

SIN3B-/-;LIN37-/- - siCTRL

SIN3A	-1.279502	-1.307502	-1.369589	-1.432589	-1.170372	-1.248372
CENPW	-0.197331	0.196669	-0.603955	-0.670955	-0.076727	-0.287727
SGO2	-1.185866	-1.394866	-1.97009	-2.55809	-2.643289	-2.499289
HJURP	-3.145998	-2.740998	-3.179437	-3.122437	-1.955312	-1.848312
CDCA5	-1.390166	-1.162166	-1.666886	-1.800886	-1.11945	-1.32645
KIF20A	0.573686	0.356686	0.398973	0.050973	1.219014	1.221014
CENPE	-1.324009	-1.570009	-1.991925	-2.133925	-0.9602	-0.8212
MCM5	-1.557271	-1.725271	-1.359734	-1.371734	-1.113475	-1.108475

SIN3B-/-;LIN37-/- - SIN3A si1

SIN3A	-2.367002	-2.519002	-1.683589	-1.694589	-1.676872	-1.826872	0.000921	***
CENPW	0.643169	0.455169	1.246045	1.227045	1.525773	0.819773	0.000922	***
SGO2	-1.671366	-1.971366	-1.78009	-1.88609	-1.829789	-1.691789	0.352556	ns
HJURP	-2.631498	-2.655498	-1.408437	-1.480437	-0.544812	-0.786812	0.041674	*
CDCA5	-1.624666	-1.702666	-0.888886	-0.844886	-0.39895	-0.66995	0.171875	ns
KIF20A	0.096186	0.156186	1.344973	0.838973	1.205514	1.192514	0.516747	ns
CENPE	-0.935509	-1.071509	-0.755925	-0.627925	-0.4097	0.3073	0.035017	*
MCM5	-1.479771	-1.504771	-0.585734	-0.634734	-0.718975	-0.783975	0.044237	*

SIN3B-/-;LIN37-/- - SIN3A si4

SIN3A	-2.336002	-2.346002	-1.886589	-1.693589	-1.800372	-1.865372	0.000145	***
CENPW	0.345169	0.907169	-0.115955	-0.317955	0.211273	0.292273	0.044677	*
SGO2	-0.941366	-0.972366	-1.18009	-1.13909	-1.736289	-1.710289	0.011804	*
HJURP	-2.246498	-2.318498	-1.518437	-1.460437	-1.205312	-1.220312	0.013634	*
CDCA5	-1.428666	-1.387666	-0.921886	-0.826886	-0.81745	-0.87845	0.108	ns
KIF20A	0.501186	0.649186	0.642973	0.394973	1.452014	1.451014	0.423421	ns
CENPE	-0.641509	-0.177509	-1.032925	-0.830925	-0.6272	-0.4202	0.007688	**
MCM5	-1.203771	-1.312771	-0.704734	-0.624734	-0.619475	-0.716475	0.008744	**

SIN3B-/-;RB-/- - siCTRL

SIN3A	-0.582031	-0.643031	-0.5135	-0.5535	-0.419033	-0.364033
CENPW	-1.254541	-1.418541	-1.378014	-1.060014	-1.734818	-1.789818
SGO2	-4.32777	-4.21177	-3.749652	-3.705652	-3.397397	-3.943397
HJURP	-6.126501	-5.403501	-6.193299	-5.577299	-5.444009	-5.174009
CDCA5	-4.669159	-4.395159	-3.54081	-3.46281	-5.342974	-4.974974
KIF20A	-4.109532	-4.221532	-3.099331	-2.665331	-2.3129	-3.6899
CENPE	-3.936592	-3.907592	-3.958089	-3.555089	-4.340262	-4.507262
MCM5	-1.064501	-0.957501	-0.190006	-0.190006	0.091	0.078

SIN3B-/-;RB-/- - SIN3A si1

SIN3A	-2.217531	-2.280531	-1.525	-1.407	-1.281533	-1.238533	2.22E-06	***
CENPW	-0.513041	-0.775041	0.899486	0.911486	0.149682	0.214682	0.006422	***
SGO2	-4.01027	-3.79627	-2.705152	-2.669152	-2.623897	-2.223897	0.018087	*
HJURP	-5.288001	-4.960001	-3.319799	-3.569799	-4.720509	-4.612509	0.043404	*
CDCA5	-3.981659	-3.981659	-2.19831	-1.70031	-4.080474	-4.127474	0.13253	ns
KIF20A	-4.723032	-3.736032	-2.296831	-1.897831	-2.0914	-3.0834	0.339873	ns
CENPE	-3.259092	-3.457092	-1.085589	-1.832589	-2.940762	-3.808762	0.068183	ns
MCM5	-1.350001	-1.070001	0.186494	0.410494	0.3365	0.3045	0.44325	ns

SIN3B-/-;RB-/- - SIN3A si4

SIN3A	-2.514531	-2.630531	-1.8355	-1.8905	-1.599033	-1.811033	1.96E-08	***
CENPW	-0.529041	-0.614041	0.023986	-0.290014	-0.507818	-0.526818	0.000545	***
SGO2	-3.07327	-3.68227	-2.300652	-1.792652	-2.547397	-2.311397	0.002036	**
HJURP	-5.379001	-5.825001	-4.171299	-4.085299	-4.382009	-4.530009	0.060254	ns
CDCA5	-4.688659	-4.878659	-3.02781	-3.01881	-4.338974	-4.391974	0.452737	ns
KIF20A	-4.239032	-4.289032	-2.321331	-2.473331	-2.8069	-2.6899	0.60735	ns
CENPE	-3.243092	-3.157092	-1.648089	-1.419089	-2.949262	-2.996262	0.020714	*
MCM5	-1.021001	-1.131001	-0.091006	0.076994	-0.092	-0.075	0.967665	ns

Fig. 7a

		DMSO						Idasanutlin						Idasanutlin+Romidepsin						T-Test DMSO Vs Idasa p values	T-Test Nutlin vs Idasa+Romi p values
CCNB2	IgG	0.0592	0.0175	0.0252325	0.01734217	0.0139647	0.021064	0.0126	0.0159	0.015738	0.00921	0.02695	0.02488514	0.0809	0.0458	0.014533	0.014983	0.07226436	0.07984955		
	H3	0.4853	0.4456	0.1765842	0.23300424	0.1383053	0.148232	0.5966	0.5872	0.396785	0.372788	0.186653	0.20538861	0.3813	0.5084	0.211309	0.258176	0.51563714	0.43359743	0.240985744	0.946206011
	H3K27ac	5.3071	5.6448	2.4732843	2.38242054	2.9158734	2.895732	1.6958	1.5325	0.339017	0.369444	1.327239	1.36455268	3.4532	3.7553	3.089617	2.528756	3.20745293	2.73289133	0.003175399	5.89904E-05
BUB1	IgG	0.0216	0.0159	0.013926	0.01939634	0.0224666	0.018723	0.0007	0.0014	0.018261	0.010798	0.022173	0.02319452	0.0283	0.0216	0.011296	0.018529	0.08026574	0.06881792		
	H3	0.5073	0.4466	0.1905086	0.2121164	0.1651591	0.164588	0.4302	0.4519	0.416367	0.412061	0.201092	0.12735505	0.4293	0.3458	0.20732	0.217929	0.39064447	0.44010757	0.502997084	0.985522315
	H3K27ac	2.9566	2.8049	2.2375627	2.27983291	2.7662434	2.871746	1.4181	1.5932	0.354026	0.442555	1.393708	1.62329825	3.3332	2.9792	3.382121	3.20412	3.24659651	3.51111219	0.00021813	6.12116E-06
NEK2	IgG	0.0228	0.002	0.0152709	0.07530702	0.0306691	0.017181	0.0034	0.0034	0.021211	0.021211	0.013007	0.10086014	0.0045	0.2687	0.010191	0.010191	0.01991402	0.00893032		
	H3	0.2912	0.338	0.3278092	0.42836963	0.3786477	0.305645	0.549	0.4481	0.541836	0.481273	0.078098	0.15361895	0.331	0.4934	0.443937	0.566217	0.68630801	0.75154328	0.732926942	0.137877208
	H3K27ac	4.0867	4.6716	3.1841937	5.1584159	3.0020086	3.688229	1.8621	1.6199	0.305645	0.189193	1.314422	0.52720213	6.8777	4.8802	6.001937	7.681713	4.70413054	4.81297222	5.90216E-05	8.78465E-06
ORC1	IgG	0.0022	0.0675	0.0975258	0.15625	0.0267268	0.056776	0.0177	0.0799	0.258624	0.255949	0.062389	0.06658902	0.0044	0.0044	0.176645	0.213444	0.22577037	0.22035906		
	H3	0.38	0.5835	0.4762953	0.42423281	0.1112154	0.211309	0.6511	1.1566	0.902456	0.786723	0.426148	0.45420897	0.4377	0.4058	0.450915	0.336558	0.37059828	0.5366034	0.021643145	0.026073071
	H3K27ac	11.991	9.7198	5.892697	6.52929894	4.1653222	4.211774	3.269178	3.2152	0.505201	0.420136	2.464727	1.51196851	10.8	9.1193	2.766243	3.179783	4.75659138	3.9066394	0.003850758	0.025545557

Fig. 7b

G2/M genes		G1/S genes		HDAC1/2 regulated genes	
Idasa	Idasa+Romi	Idasa	Idasa+Romi	Idasa	Idasa+Romi
-4.85054583	-4.85754583	-4.504475	-3.96747466	0.609922	3.080903504
-6.99600035	-8.16300035	-4.398765	-5.14676545	0.3285362	5.035517703
-6.53467086	-7.21967086	-5.345525	-4.92752504	-0.453525	1.703456442
-5.86100555	-5.34500555	-5.266375	-4.72737511	-1.13945	2.190531899
-7.39850702	-7.46450702	-3.756619	-3.34661861	-0.777375	1.445606368
-6.71069278	-6.15859278	-4.670502	-5.01350217	-1.769168	2.203813746
-4.33100866	-3.87500866	-3.633555	-4.61955521	1.9170282	3.72200971
-4.18590125	-4.42890125	-3.613126	-3.76112591	0.685922	3.106903504
-7.09833666	-7.84333667	-5.217266	-4.79526593	0.3075362	5.066517703
-6.01824731	-5.74324731	-6.204882	-5.40488188	0.149475	1.702456442
-5.72827322	-4.96727322	-3.794912	-3.34991247	-1.64545	2.179531899
-6.05999544	-6.19699544	-5.353897	-3.61389702	-1.010375	1.337606368
-3.84256316	-4.30956316	-3.713846	-2.6568461	-1.318168	2.080813746
-7.50458789	-8.15358789	-4.694475	-4.33747466	2.0230282	3.68300971
-6.26623218	-7.07823218	-4.107765	-5.21776545	0.8336849	2.707018259
-3.56542272	-4.80942272	-5.678525	-5.03752504	1.7295773	6.731910618
-2.85731189	-4.79131189	-5.448375	-5.21337511	-1.033076	1.001257143
-5.54480158	-4.27880159	-3.636619	-3.14961861	-1.962768	0.899565159
-4.91738367	-4.93138367	-4.727502	-5.02650217	-0.655646	1.475687691
-4.99954583	-4.70154583	-2.763555	-3.82855521	-1.659001	1.519331947
-6.93400035	-7.64500035	-3.729126	-2.98612591	1.9676226	2.886955948
-6.67567086	-7.03067086	-4.502266	-4.00326593	1.2226849	3.189018259
-5.99400555	-5.40000555	-5.632882	-4.66388188	2.1165773	7.120910618
[illegible]	[illegible]	[illegible]	[illegible]	[illegible]	[illegible]

P value [illegible]
P value summary [illegible]

	Idasa				Idasa+Romi				T-Test / p values	
KIF20A	13.51843	16.28934	12.25124	16.8521	3.537809	5.66413711	2.5524031	2.8775803	0.000146	***
CENPW	8.03254	5.675928	6.193935	8.537752	3.391342	3.23520289	1.68279121	1.85042439	0.001477	**
SGO1	7.036491	10.1602	5.425876	3.441067	5.090672	8.68698887	4.800516119	4.093087542	0.645677	ns
KIF23	4.985908	6.202527	2.043236	2.292398	6.839336	6.31094561	2.75335288	2.588631348	0.643808	ns
PLK1	5.695633	5.425876	1.303921	1.159784	4.812732	4.87991558	1.531759207	1.224497241	0.865232	ns
BUB1	3.493943	3.151101	1.93973	3.467405	3.477032	3.87408656	1.749799381	3.607975453	0.795543	ns
CENPE	3.197305	2.859685	2.640582	1.983236	3.166428	4.18682376	2.73939288	4.16655837	0.091073	ns
AURKA	2.180798	6.36807	0.531395	0.594544	3.695722	7.22930115	0.618647504	0.636481075	0.774001	ns
SGO2	2.186853	2.335702	1.841468	2.056023	5.259247	4.30453139	3.3100573	3.50850444	0.004725	**
CDCA5	1.448798	2.463751	1.241304	2.403033	0.825223	1.73972128	1.46698711	1.92499699	0.353816	ns
UBE2C	1.60198	2.398041	1.390743	1.363067	1.456854	2.04323648	1.581385962	1.495045636	0.878456	ns
NEK2	1.725311	1.573368	1.370646	1.376358	2.467168	2.37488259	2.906311869	2.247295008	0.00103	**
DLGAP5	1.609772	2.76036	0.242298	0.216563	1.947814	5.12963506	0.339195638	0.343454174	0.588815	ns
CDC25C	1.021618	1.818635	0.573496	0.324402	1.497812	3.06918624	0.640908151	0.756907823	0.423527	ns
FOXM1	1.046706	0.949247	0.303729	0.596195	0.651057	0.7421878	0.318020026	0.882207251	0.729038	ns
CCNB2	0.783962	0.818388	0.42099	0.540683	0.349135	0.49995016	0.449436601	0.53373278	0.129064	ns
HJURP	0.62627	0.628009	0.49995	0.386049	0.399386	0.53657665	0.54331327	0.64925458	0.970963	ns
MKI67	0.594544	0.655131	0.227487	0.263862	0.567958	0.52626312	0.275321565	0.294877883	0.891159	ns
BIRC5	0.68675	0.510101	0.145577	0.171568	0.409761	0.55166188	0.135765566	0.123380775	0.679469	ns
RBL1	7.254399	7.314992	12.48268	11.49438	15.09351	16.1656116	26.68929928	25.00577689	0.014751	*
RAD54L	8.449443	15.44273	4.863032	4.57844	4.265918	7.38120413	1.431164516	1.740123286	0.159176	ns
E2F8	7.304858	7.938444	6.601785	9.062168	9.705861	11.1259467	9.701376914	9.627688889	0.010836	*
CDC6	4.186824	3.670194	6.939618	7.199298	6.074881	4.70063672	7.439416774	9.396930139	0.34052	ns
BRCA1	7.381204	8.315807	2.326008	2.56304	10.04814	14.4987457	3.178155079	2.561856245	0.486093	ns
MCM5	4.591152	5.61722	4.613483	4.553122	2.733702	2.60242428	2.721099145	3.602977202	0.001425	**
GINS2	8.42021	7.769685	1.138281	0.973232	7.599246	13.0037533	2.043708624	2.124597458	0.642965	ns
RAD51	3.933616	3.781232	2.484329	2.214308	3.101264	3.07344398	1.619097116	1.541344686	0.260115	ns
E2F1	2.716702	2.394719	2.571939	4.123462	3.947273	2.81836056	2.85638317	5.378446483	0.31061	ns
ORC1	2.445037	1.941075	3.438683	2.796953	3.26675	3.02693184	4.317480085	4.240368143	0.059768	ns
ATAD2	2.911689	4.779488	1.246477	1.256888	3.901033	6.75453376	1.175430732	1.086877633	0.682585	ns
MYBL2	2.557716	4.175231	0.973907	1.014561	8.543672	6.51089643	1.13146309	1.032534168	0.340564	ns
ZNF367	1.470041	2.185338	0.707036	0.914374	2.55949	4.27776192	1.288050826	1.312383648	0.230014	ns
HIST1H2BI	340.8176	366.8016	382.3781	423.3938	1193.395	1161.56662	723.1692497	712.2251265	0.005396	**
CTGF	120.1636	118.4272	349.6714	457.2553	3145.026	3213.33614	11207.59823	14676.1865	0.0364	*
ARRDC4	151.0473	159.2176	184.4206	241.4961	839.1915	854.452326	675.6768064	943.7032904	3.66E-05	***
VGF	73.45843	111.5739	55.74831	67.17517	328.2966	328.069164	228.3636985	276.7020502	0.000211	***
RGL1	61.00503	51.90696	62.58982	63.37563	285.4029	264.817782	274.2199119	289.4538475	3.37E-08	***
DHRS2	29.79206	40.72538	31.62199	26.75722	468.8089	430.49605	286.2614368	250.0702323	0.000885	***
MTX1	40.33209	28.40073	24.62166	21.00784	406.4281	403.340982	179.0463779	201.9969253	0.004948	**

P value 0.001 Ratio Paired T-Test
P value sum ***

Fig. 7d

G2/M genes		G1/S genes		HDAC1/2 regulated genes	
Idasa	Idasa+Romi	Idasa	Idasa+Romi	Idasa	Idasa+Romi
[illegible]	[illegible]	[illegible]	[illegible]	[illegible]	[illegible]

	Idasa				Idasa+Romi				T-Test / p values	
SGO1	12.01507	11.68652	12.65045	10.83867	9.995869	13.1076031	20.05360513	19.1170726	0.173407	ns
AURKA	5.380624	5.990903	4.708007	5.162666	8.411319	5.92301602	6.583203134	6.354548903	0.048322	*
KIF23	4.742913	4.195299	5.18059	3.396659	14.37343	13.1987737	22.12782451	19.03773203	0.000909	***
PLK1	4.613218	5.0203	4.129927	4.112787	3.717498	3.95953768	5.974382948	5.601408771	0.593092	ns
IQGAP3	2.997529	3.339827	3.506706	3.704083	4.258461	5.31228294	9.303604154	8.309661534	0.030202	*
BUB1	2.573239	2.814106	3.030051	2.727318	4.68195	4.98879323	10.12765163	8.661397863	0.018699	*
DLGAP5	2.080274	2.616745	2.622816	2.781988	3.907733	4.43929933	5.358353327	5.770873532	0.002027	**
FOXM1	0.660108	0.695332	3.575429	4.783665	1.298037	2.60870171	8.472511646	9.245745908	0.237586	ns
CDC25C	1.523906	2.088944	2.635573	1.477457	2.97385	3.454153	10.24458555	7.949090014	0.055835	ns
UBE2C	1.193148	1.084307	0.950736	0.984263	2.164947	2.25531975	4.120415185	3.754939635	0.00727	**
NEK2	1.242548	0.659666	0.573903	0.448801	3.202175	3.28701794	5.628620334	5.332058384	0.001657	**
BIRC5	0.646523	0.717362	0.681184	0.71406	1.007886	0.95550279	1.870974265	1.773734055	0.026784	*
MKI67	0.45843	0.600307	0.245045	0.283048	2.024172	2.19060898	4.674427607	4.687405855	0.00707	**
CCNB2	0.191946	0.20558	0.235226	0.271141	0.61828	0.66541763	0.717370197	1.0723603	0.002065	**
RBL1	25.96999	26.35078	19.782	19.64536	29.12794	29.5550343	48.29459113	48.227687	0.033343	*
GINS2	15.27154	12.77962	13.1968	12.87157	29.26962	29.2696175	40.21856536	37.52524835	0.000387	***
ATAD2	11.45395	11.94035	10.97475	12.668	15.94831	14.8906207	20.58882674	18.54283226	0.005137	**
E2F8	10.03364	11.65416	9.177597	9.260669	37.40948	37.8004627	54.44734194	61.04451283	0.00076	***
BRCA1	8.240738	8.478291	7.917892	7.43904	9.191702	8.63581291	14.49807764	13.25798587	0.062241	ns
MCM5	7.375667	7.284215	4.904535	6.685875	10.29115	10.2911478	11.67046156	11.67046156	0.000728	***
RAD51	5.53573	4.391682	3.917997	3.415546	7.024191	7.4919018	7.790898476	7.556874769	0.000603	***
ORC1	5.20618	4.833238	2.560051	2.470508	10.2805	9.55348326	12.19482298	12.51993404	0.000367	***
ZNF367	3.314459	3.574579	3.869417	3.964438	11.00687	12.6698891	26.44255567	26.44255567	0.010668	*
E2F1	3.135669	3.488903	4.918152	2.452268	13.0532	13.0713114	23.37330295	19.06414223	0.001804	**
CDC6	2.785186	2.675435	2.190282	2.61012	14.72639	15.92622	30.12291987	30.01870249	0.003258	**
RAD54L	1.841362	2.078832	2.41181	1.681929	4.602227	4.69241948	11.52575789	12.14078427	0.02425	*
MYBL2	1.42284	1.696749	1.621261	1.604492	3.091554	2.59787495	6.066185919	5.429388482	0.019422	*
DHRS2	533.6587	441.6542	705.3113	599.2924	10172.11	10538.1353	15001.19401	14907.90298	0.0001	***
ARRDC4	483.2994	402.2012	494.2566	478.0827	2966.06	2859.07349	5248.864775	5385.220547	0.00192	**
MTX1	143.5862	151.563	272.6897	297.7828	1251.404	1191.30903	1558.335025	2688.880109	0.005983	**
RGL1	154.6404	152.723	223.3429	193.8929	1258.363	1278.58467	1787.577053	1681.799161	7.27E-05	***
VGF	91.56812	83.2728	152.9712	149.2013	473.5366	348.818317	627.203463	539.2422455	0.000848	***
CTGF	39.47034	38.48003	54.57961	55.18635	1069.258	1043.70056	2165.206015	2139.819111	0.002654	**

P value 0.0006 Ratio Paired T-Test
P value sum ***

Fig. 7f

G2/M genes		G1/S genes		HDAC1/2 regulated genes	
Idasa	Idasa+Romi	Idasa	Idasa+Romi	Idasa	Idasa+Romi
[illegible]	[illegible]	[illegible]	[illegible]	[illegible]	[illegible]

	Idasa				Idasa+Romi				T-Test / p values	
Bub1	5.056385	5.105691	3.901033	4.063875	3.746014	3.92952843	6.294562858	5.610409942	0.624111	ns
Nek2	4.91471	5.464563	5.371616	6.789739	13.254	13.26319264	12.50649994	11.75828861	1.23E-05	***
Ttk	18.3487	23.32178	11.56631	17.51909	14.72153	19.07824011	17.84077262	16.09015356	0.780392	ns
Foxm1	3.082511	4.296334	3.62092	6.813312	4.761302	5.964313906	5.928249593	7.390163023	0.164802	ns
Ccnb2	3.727236	4.389651	2.297964	2.805691	3.106642	3.213969392	2.919267045	2.837472066	0.569358	ns
Aurkb	13.82403	14.23239	6.978206	13.10328	10.03074	11.13752056	6.812131051	9.481440025	0.216668	ns
Kif11	2.685343	2.86018	5.052005	3.138024	2.637838	3.1347626	7.180608916	7.270760077	0.281474	ns
Ube2c	3.971286	5.331739	2.658949	3.602145	6.219748	6.768594035	9.409422559	10.21846218	0.009073	**
Kif23	10.42956	11.97212	7.694653	8.449443	10.29489	11.80115524	15.63932335	17.91512482	0.075909	ns
Plk1	12.78257	10.34675	11.67504	10.62474	8.511162	10.03769556	14.41607173	13.76188502	0.839537	ns
Ccnb1	8.236927	7.145853	5.613327	4.591152	9.236563	10.10751325	7.637530254	6.974579262	0.101033	ns
Birc5	2.579526	3.145101	2.081114	2.377353	2.571047	3.590926664	3.661935017	3.571688318	0.057994	ns
Cdc25c	5.722344	4.974688	2.28922	3.22401	3.316948	4.385849144	4.67869502	4.860504913	0.774233	ns
Cks1	19.82304	22.0714	18.88745	18.80906	18.43475	17.58600879	27.25796946	27.40953959	0.360391	ns
Cks2	10.52396	11.9307	10.4386	11.15684	12.23427	11.58235077	14.8059606	17.14963766	0.068402	ns
Plk4	16.18523	23.99407	10.28775	10.4821	8.03254	9.814101483	9.822608439	10.91398597	0.138951	ns
Sgol1	8.724704	7.116196	5.741215	7.196803	7.256914	6.813311606	10.78987458	10.63395374	0.220525	ns
Mcm5	5.389331	6.931205	5.825396	6.436856	3.803575	4.573682408	6.614381184	6.442435507	0.347558	ns
Cdc45	5.538926	9.328248	7.026743	3.762929	11.01087	12.94081155	14.70368836	11.08169138	0.006388	**
E2f2	9.393131	11.96382	9.705861	12.81584	15.86589	17.54339658	17.65623938	19.91945389	0.001227	**
E2f7	10.68197	10.1268	9.391504	10.24151	25.55186	34.23395786	34.83839341	42.53581494	0.000442	***
Mybl2	4.62549	3.677196	5.448489	5.292158	6.515411	4.31648265	5.856773857	7.511526845	0.150429	ns
E2f8	14.39111	14.76497	17.89961	26.37056	6.256502	10.44945787	11.31454128	17.72982252	0.107357	ns
Atad2	40.12991	42.44746	16.85794	16.3856	21.82419	19.06502067	23.35413636	21.63966395	0.338152	ns
Orc1	28.45492	23.77884	16.78797	16.78797	11.57433	8.887983816	14.96589019	12.01367961	0.021722	*
Cdc6	2.062803	2.945691	2.262403	2.127053	3.887536	3.212855708	4.178850625	4.67869502	0.004261	**
Mcm3	12.18561	13.92983	11.02996	10.69123	9.281488	9.242968009	12.85810552	11.24028094	0.294851	ns
Ctgf	88.19385	103.3652	116.2314	137.4589	1319.574	1246.663327	1353.150123	1289.956043	5.59E-09	***
Vgf	96.94578	98.98282	145.599	194.3962	2607.328	1646.693334	3937.614581	2551.458021	0.00166	**
Rgl1	148.5296	123.5205	191.9193	168.9387	593.398	532.5806453	925.8317253	1018.765426	0.002384	**

P value 0.0297 Ratio Paired T-Test
P value sum *

Fig. 7h

G2/M genes		G1/S genes		HDAC1/2 regulated genes	
Idasa	Idasa+Romi	Idasa	Idasa+Romi	Idasa	Idasa+Romi
[illegible]	[illegible]	[illegible]	[illegible]	[illegible]	[illegible]

	Idasa				Idasa+Romi				T-Test / p values	
KIF23	11.23055	12.75823	11.39916	10.91209	12.63064	12.42226367	11.01468391	11.57833733	0.490397	ns
BUB1	8.750444	12.38358	11.00324	9.692415	15.36799	16.7125062	15.6745942	12.1708345	0.012869	*
AURKA	10.78801	9.926989	3.632232	9.807301	4.192632	6.176785322	7.586088434	8.376550103	0.44599	ns
UBE2C	6.682353	6.714855	9.951101	10.02726	6.682353	6.71485474	8.99021856	9.50282321	0.208022	ns
PLK1	7.902758	8.130568	6.574386	6.978206	4.655242	4.76625517	4.81773884	4.38432939	0.005059	**
CCNB2	5.250141	6.975788	7.065816	6.638495	3.472215	4.91045323	4.00376192	4.48577168	0.003855	**
FOXM1	7.08789	5.264718	4.404127	3.215083	6.172505	7.69465258	4.32696722	5.33451159	0.357782	ns
DLGAP5	5.067788	5.745196	3.757716	3.958232	8.258365	7.960484221	7.766993129	6.196081797	0.006583	**
CDC25C	2.809583	4.454786	5.284827	3.855335	11.54228	10.5039219	9.109400993	13.08059435	0.011598	*
NEK2	2.142591	0.782605	1.059113	1.914352	4.684374	4.37370583	6.47264863	7.30738991	0.009346	**
BIRC5	1.161796	1.296261	1.49885	1.208197	1.505097	1.76034254	1.76217376	1.49729261	0.00469	**
MKI67	1.177197	0.663586	0.992994	1.13907	2.424784	2.23900258	2.94722293	2.94110072	0.001741	**
ATAD2	30.96049	26.85942	39.88726	43.52753	27.56673	31.1866393	43.72408201	39.70795589	0.92162	ns
GINS2	28.09723	28.23389	32.75984	34.89277	32.69178	33.98570605	53.64441936	48.75087438	0.059594	ns
BRCA1	27.90315	13.31847	31.73177	30.86407	27.35734	25.8995691	30.6190197	30.1974786	0.498725	ns
MCM5	17.79446	16.21611	18.03661	18.65973	26.0978	27.3573425	28.7872124	27.1966338	0.000945	***
RAD51	13.07153	13.06247	15.81099	15.23013	11.46255	12.3364568	12.0032751	12.3664216	0.045228	*
CDC6	11.03761	9.783537	18.56941	15.27241	62.93786	54.6010273	64.0601908	70.588252	0.000298	***
RAD54L	10.49301	8.576305	10.76933	7.59398	14.40858	13.12145855	17.05775868	12.76707554	0.002228	**
ORC1	8.023016	9.491144	10.86034	6.650428	32.65503	33.3598948	39.368542	36.9767337	0.000416	***
E2F1	6.339293	6.221935	12.87724	9.134255	21.96391	23.1561998	40.1030463	47.2489474	0.018479	*
B-MYB	5.56876	8.458232	9.97873	8.993335	6.844078	8.1333866	12.3921636	10.8781113	0.113865	ns
ZNF367	2.711998	2.456078	2.90564	3.698284	13.12146	12.99474294	19.7168365	20.68275106	0.005148	**
DHRS2	1331.98	1331.98	3310.552	3451.137	55027.16	55333.1406	118650.297	90985.9518	0.013648	*
RGL1-2	214.429	211.4769	338.4634	346.2946	396.6867	387.715927	487.871092	600.223581	0.003385	**
CTGF	168.9448	126.2933	231.3275	229.44	274.851	271.212896	431.62016	440.878438	0.006733	**
VGF	94.70418	84.76261	316.0165	264.4509	273.9666	335.659855	845.321416	744.617746	0.024468	*
MTX1	48.48129	53.45882	42.69089	38.55527	526.3418	547.174122	566.273886	626.146394	0.000221	***

-4.1905785	-3.7200785	-2.828358	-1.86885758	
-4.521724476	-3.87522448	-1.629602	-1.66110196	
-3.365366037	-4.04986604	-3.584653	-3.64115283	
-3.232694533	-3.46019453	-3.435068	-3.16056839	
-4.020943508	-3.96944351			
-3.406561742	-3.48406174			
-2.898871468	-2.93737147			
-3.113973208	-3.02847321			

P value	0.1201	P value	0.0105	P value	0.0485	Ratio Paired T-Test
P value summary	ns	P value sun	*	P value sum	*	

Supplementary Fig. 1a

		WT				SIN3B-/-				LIN37-/-				SIN3B-/-;LIN37-/-				T-Test / p values		
																		WT vs SIN3B-/-	WT vs LIN37-/-	WT vs SIN3B-/-;LIN37-/-
BUB1	0	100	96.5267025	100	103.096832	100	117.121	100	101.6775	100	98.82857	100	158.117823	100	105.921833	100	104.3912			
	48	46.2973	42.8094142	11.797066	14.1954452	52.814281	60.54165	96.99619	83.5667	125.6578	132.6385	191.1228	196.768598	118.2631	102.740144	78.24118	67.12863	0.017208653	0.000714026	0.005358403
	72	9.195042	12.0157616	5.2720213	5.33821048	43.271829	45.42316	40.15078	39.79061	77.40507	76.18083	98.00994	98.8285652	47.8304079	47.204621	74.63892	78.40405	3.54149E-06	1.73619E-05	0.000738539
	96	7.082979	10.654243	5.0310384	6.7405027	29.504359	28.71746	32.5561	33.37871	63.00333	72.37182	74.97596	67.6658587	47.7641468	55.9806444	79.38839	78.89468	6.93263E-06	6.67319E-07	0.00037874
	120	5.192237	7.23180707	7.1669346	7.1223641	23.70889	23.31774	36.58946	35.44122	87.90396	87.41786	59.72886	56.0388789	60.8150374	62.8724549	73.84131	69.5682	0.000731506	0.00026122	1.10234E-06

		WT				SIN3B-/-				LIN37-/-				SIN3B-/-;LIN37-/-				T-Test / p values		
																		WT vs SIN3B-/-	WT vs LIN37-/-	WT vs SIN3B-/-;LIN37-/-
CCNB2	0	100	104.174343	100	95.1318276	100	92.40226	100	92.53044	100	75.419	100	93.7571096	100	85.1453708	100	90.0626			
	48	44.16575	41.6387885	11.098988	12.3921636	54.563194	47.17191	64.97695	62.28687	138.7512	130.7218	130.1793	113.249133	107.698638	81.5637493	64.52812	64.48341	0.023024394	7.21673E-05	0.008580965
	72	7.683993	8.74741166	4.5483907	5.76114748	40.909279	37.74873	40.68306	39.70796	101.0802	86.24424	57.47474	56.2139462	47.1392268	48.3973513	65.24775	62.07137	1.40862E-07	0.000825271	4.74663E-05
	96	5.224729	6.14475798	3.3008926	3.96922182	22.159543	25.86369	27.68161	23.52065	76.81713	70.6372	36.53877	38.0640242	48.9031737	48.4309095	65.38357	70.907	6.51439E-06	0.003028045	8.67532E-05
	120	4.30304	3.81678042	3.9023852	5.20304502	16.070648	14.81879	23.43116	23.84072	95.2638	76.15444	32.34243	36.2361102	48.6158955	47.5164842	65.90684	64.10461	0.00071778	0.011055511	4.11165E-05

		WT				SIN3B-/-				LIN37-/-				SIN3B-/-;LIN37-/-				T-Test / p values		
																		WT vs SIN3B-/-	WT vs LIN37-/-	WT vs SIN3B-/-;LIN37-/-
BIRC5/ Survivin	0	100	97.3329374	100	99.8614666	100	104.7536	100	102.8114	100	100.7654	100	133.329868	100	101.88912	100	105.4822			
	48	39.06639	36.2486708	9.5821951	9.74968893	60.37403	57.59438	81.6203	80.05148	128.1204	118.9619	129.1905	130.721822	94.8684315	81.2815602	59.91545	59.99857	0.004124151	1.94045E-05	0.00527538
	72	8.999571	8.75347701	4.4982261	4.43322378	58.2165	56.42866	48.88623	49.87885	93.65968	93.52993	72.14643	69.1116103	29.6478625	35.9733395	75.4713	69.44776	2.17484E-06	3.13944E-05	0.007588054
	96	8.295657	7.98535303	3.7996228	3.02483446	32.941999	32.26406	35.18421	35.08679	55.8063	55.61323	45.61246	54.2802774	42.4548123	41.3225159	80.16253	80.66418	1.91479E-06	2.78607E-06	0.002603095
	120	6.594925	6.64540069	4.6892467	4.4087088	26.508005	25.32264	42.67609	42.3226	67.68931	67.315	48.31356	48.3470578	58.3781339	55.9224704	71.92175	72.57276	0.00103101	8.23567E-05	1.0956E-05

																		T-Test / p values		
																		WT vs SIN3B-/-	WT vs LIN37-/-	WT vs SIN3B-/-;LIN37-/-
MCM5	0	100	106.215919	100	106.437018	100	101.6775	100	99.10296	100	93.95227	100	98.6916546	100	100	100	100.9052			
	48	24.74142	22.9569041	20.539885	20.2992735	44.288376	27.37631	103.6701	99.17167	178.0769	174.654	130.9032	125.65778	107.549439	88.5153765	132.6845	128.7882	0.0526217	8.67993E-05	0.000107861
	72	15.3467	15.1879599	4.5075897	10.9158774	45.015695	43.63325	41.82681	40.79601	140.0071	124.4443	94.27845	99.3781093	71.2025098	69.1595315	130.2244	130.4955	2.52565E-05	8.60868E-05	0.002321739
	96	12.30656	11.5703146	12.534705	11.809338	28.440131	28.24368	45.09377	42.45481	95.76033	98.99997	65.49697	71.5736272	75.7333158	75.9435845	137.9361	140.5419	0.001757539	0.00015575	0.001986803
	120	13.21731	13.4669962	10.878111	11.0146839	20.826611	20.76895	54.09248	47.38492	100.4864	95.59453	61.1532	61.4080629	68.8486371	71.0792321	114.7505	114.195	0.035998523	0.000732485	0.000798542

																		T-Test / p values		
																		WT vs SIN3B-/-	WT vs LIN37-/-	WT vs SIN3B-/-;LIN37-/-
ORC1	0	100	94.2131274	100	103.239854	100	103.2399	100	95.85994	100	91.955	100	101.81852	100	96.1260928	100	97.87417			
	48	18.72451	19.1179535	10.573317	12.0867644	55.748311	55.67108	99.44702	97.73858	110.3051	118.4682	83.71163	81.988871	75.4712984	61.8566239	69.39964	64.61764	0.002629579	0.000121963	7.67087E-06
	72	11.33613	17.3138684	5.7851573	7.23180707	43.452166	42.76493	44.76677	47.7807	105.8118	98.1119	48.26335	45.5966583	35.1842094	36.1733721	70.85787	68.01854	1.85075E-05	0.007475901	0.005863734
	96	7.756233	8.75044381	7.8508899	7.47905602	29.709578	29.3412	48.43091	45.53349	63.04702	65.99826	36.46287	34.1865324	45.7866843	41.3511684	77.64689	81.00035	0.000997891	0.002562563	0.002116806
	120	11.26954	10.654243	9.9960366	10.3413755	26.729412	23.64325	36.38713	35.39212	96.7947	94.8027	34.80822	37.5399856	52.0330435	49.9134316	74.09767	69.76136	0.000747016	0.018208027	0.000166441

Supplementary Fig. 1b

	C2C12-WT									C2C12-SIN3B-/-								
	clone 1			clone 2			clone 3			clone 1			clone 2			clone 3		
proliferating	47.9	52.1	52.6	40.9	40	40.2	46.7	45.7	47	50.6	49.5	51.6	44.9	46.9	46	44.8	48.6	47.9
starved	11	12.5	10.8	19.2	19.5	17.5	7.03	6.05	6.46	3.99	6.31	5.36	10.4	12.3	11.1	11.1	11.8	12

T-Test WT vs SIN3B-/-	proliferating	0.286201
	starved	0.19087

Supplementary Fig. 2b

	BUB1		*NEK2*		*CCNB2*		*MCM5*		*ORC1*		*CDC6*	
siCTRL	100	98.62327	100	93.303299	100	92.466328	100	105.0445	100	100.4168	100	93.56235
1	19.4656173	16.99285	17.7882933	16.910603	14.1806936	11.719639	28.93726	28.59827	36.70377	35.60125	27.05562	27.03687
2	42.5284447	43.33186	47.6153948	40.626698	32.6578111	31.742772	60.73079	59.68747	67.43175	63.09073	75.07997	73.73901
3	100.486382	100.0693	87.6605721	88.761134	43.4973676	50.138822	118.0993	108.7488	133.6074	139.1846	140.5419	147.8362
4	33.7041933	29.83339	33.8681244	30.735972	35.8488812	20.862732	49.96535	50.83873	64.61764	58.64175	32.33122	32.42099

Supplementary Fig. 2d

	WT - siCTRL		WT - siSIN3A-1		WT - siSIN3A-2		WT - siSIN3A-4		SIN3B$^{-/-}$ - siCTRL		SIN3B$^{-/-}$ - siSIN3A-1		SIN3B$^{-/-}$ - siSIN3A-2		SIN3B$^{-/-}$ - siSIN3A-4	
NEK2	-5.0971123	-5.463112	-5.7826123	-5.895612	-4.7271123	-4.452112	-5.377112	-5.572112	-5.537153	-5.625153	-4.686153	-5.509153	-5.0351528	-5.1641528	-5.3851528	-5.373153
BUB1	-5.168419	-4.924419	-5.120919	-4.776919	-4.700419	-4.668419	-4.774419	-5.113419	-4.943425	-5.155425	-4.097425	-4.696425	-4.0984245	-4.2144245	-4.4704245	-4.492425
CCNB2	-4.8451251	-4.841125	-4.5456251	-4.279625	-5.3301251	-5.267125	-5.562125	-4.937125	-5.900611	-5.512611	-4.419611	-4.394611	-5.0936113	-4.9416113	-4.5356113	-4.859611
ORC1	-5.1177253	-4.989725	-4.5092253	-4.528225	-4.1427253	-4.137725	-4.547725	-4.874725	-5.564125	-5.562125	-4.164125	-4.048125	-3.8081251	-3.6791251	-4.1671251	-4.233125
MCM5	-4.0981251	-4.100125	-3.8896251	-3.860625	-4.0091251	-3.955125	-4.199125	-4.021125	-4.159611	-4.089611	-2.632611	-2.541611	-2.9676113	-2.9236113	-3.1666113	-3.479611
CDC6	-4.5581251	-4.286125	-4.7756251	-4.716625	-4.4221251	-4.571125	-5.113125	-5.219125	-4.807611	-4.822611	-3.742611	-3.837611	-3.7086113	-3.7176113	-4.2206113	-4.464611

		WT - siSIN3A-1		WT - siSIN3A-2		WT - siSIN3A-4		SIN3B$^{-/-}$ - siCTRL	SIN3B$^{-/-}$ - siSIN3A-1		SIN3B$^{-/-}$ - siSIN3A-2		SIN3B$^{-/-}$ - siSIN3A-4	
NEK2		0.127098326	ns	0.09177653	ns	0.445426	ns		0.378388	ns	0.029284084	*	0.039936258	*
BUB1		0.677247051	ns	0.081667984	ns	0.661257	ns		0.215184	ns	0.014668173	*	0.023212247	*
CCNB2	T-Test / p values	0.104814446	ns	0.003471819	**	0.300842	ns		0.008723	**	0.062874946	ns	0.061174296	ns
ORC1		0.010110239	*	0.002531813	**	0.209426	ns		0.003967	**	0.00386087	**	0.001396779	**
MCM5		0.004862328	**	0.053003959	ns	0.934274	ns		0.002466	**	0.001112512	**	0.058737091	ns
CDC6		0.165230853	ns	0.664934685	ns	0.052311	ns		0.004182	**	5.46577E-05	***	0.079246135	ns

Supplementary Fig. 2e

	WT - NS si						WT - SIN3A si1						WT - SIN3A si4					
BUB1	-4.99874347	-5.480743	-5.11168333	-5.802683	-5.18368333	-4.762683	-4.902244	-4.907244	-5.635683	-5.663683	-4.968683	-4.917683	-4.8592435	-5.1002435	-5.8246833	-5.907683	-5.806683	-5.8066833
NEK2	-5.76588692	-5.392887	-5.00568333	-5.621683	-5.99783303	-6.055833	-4.715387	-4.898387	-5.136683	-5.732683	-5.741833	-6.212833	-5.3993869	-4.2703869	-5.0356833	-5.387683	-7.062833	-6.568833
ORC1	-4.64887124	-4.351871	-4.86406072	-5.285061	-4.60804583	-5.082046	-4.000371	-3.461371	-3.998061	-4.286061	-5.296046	-5.513046	-4.0283712	-4.4823712	-4.1550607	-4.403061	-4.820046	-5.2290458

	SIN3B$^{-/-}$ - siCTRL						SIN3B$^{-/-}$ - SIN3A si1						SIN3B$^{-/-}$ - SIN3A si4					
BUB1	-5.3406191	-5.3406191	-5.3349281	-5.598928	-4.0606	-4.4696002	-3.9786191	-4.931619	-4.272428	-4.650428	-4.6881	-4.7011	-4.522119	-4.718119	-4.990928	-5.341928	-4.6041	-4.5531
NEK2	-5.5731102	-5.2101102	-5.8440729	-6.060073	-6.120528	-5.2985281	-4.7751102	-4.53611	-4.895573	-5.182573	-5.872028	-5.646028	-5.44861	-4.71761	-5.586073	-5.268073	-6.624028	-6.290028
ORC1	-6.6554518	-6.2704518	-5.1390004	-5.569	-5.481747	-5.2447473	-5.5214518	-5.519452	-3.6875	-3.9145	-4.848247	-4.891247	-4.578952	-4.981952	-3.968	-4.103	-4.542247	-4.403247

	SIN3B$^{-/-}$;LIN37$^{-/-}$ - NS si						SIN3B$^{-/-}$;LIN37$^{-/-}$ - SIN3A si1						SIN3B$^{-/-}$;LIN37$^{-/-}$ - SIN3A si4					
BUB1	-1.767372	-1.920372	-1.944225	-1.955225	-1.714611	-1.688611	-1.854872	-1.921872	-1.347225	-1.423225	-1.399111	-1.366111	-1.533872	-1.549872	-1.021225	-1.061225	-1.123611	-1.123611
NEK2	-2.502973	-2.487973	-2.722344	-2.700344	-2.497765	-2.514765	-1.811473	-1.829473	-1.148344	-1.080344	-1.773265	-1.815265	-1.684473	-1.563473	-1.244344	-1.310344	-1.587765	-1.550765
ORC1	-2.324822	-2.397822	-2.312501	-2.286501	-2.191068	-2.281068	-1.720322	-1.557322	-0.556501	-0.496501	-1.134568	-1.077568	-1.443322	-1.457322	-0.651501	-0.704501	-0.923068	-0.816068

	SIN3B$^{-/-}$;RB$^{-/-}$ - NS si						SIN3B$^{-/-}$;RB$^{-/-}$ - SIN3A si1						SIN3B$^{-/-}$;RB$^{-/-}$ - SIN3A si4					
BUB1	-4.139228	-4.546228	-4.575698	-4.801698	-4.590923	-4.410923	-4.701728	-4.053728	-3.561198	-3.952198	-4.372423	-4.015423	-4.917728	-4.963728	-3.620698	-3.921698	-4.236923	-4.244923
NEK2	-5.204191	-5.147191	-5.303538	-5.086538	-5.401027	-5.484027	-5.558691	-5.443691	-3.467038	-3.257038	-5.164527	-4.764527	-5.503691	-5.770691	-3.683538	-4.143538	-4.771027	-4.623027
ORC1	-2.145459	-2.093459	-1.770291	-1.599291	-1.753781	-1.828781	-0.686959	-1.353959	-0.462791	-0.509791	-1.183281	-1.244281	-1.465959	-2.093959	-0.739291	-0.925291	-1.376781	-1.437781

		WT - SIN3A si1		WT - SIN3A si4		SIN3B$^{-/-}$ - SIN3A si1		SIN3B$^{-/-}$ - SIN3A si4		SIN3B$^{-/-}$;LIN37$^{-/-}$ - SIN3A si1		SIN3B$^{-/-}$;LIN37$^{-/-}$ - SIN3A si4		SIN3B$^{-/-}$;RB$^{-/-}$ - SIN3A si1		SIN3B$^{-/-}$;RB$^{-/-}$ - SIN3A si4	
BUB1		0.719362	ns	0.207436769	ns	0.149051	ns	0.489126	ns	0.040708	*	0.000819	***	0.072015	ns	0.320383	ns
NEK2	T-Test / p values	0.258396	ns	0.565158468	ns	0.04936	*	0.716946	ns	0.000706	***	1.24E-06	***	0.142022	ns	0.123065	ns
ORC1		0.174209	ns	0.180958166	ns	0.052878	ns	0.001062	**	0.002543	**	0.000132	***	0.001712	**	0.014942	*

Supplementary Fig. 3a

G2/M genes		G1/S genes		HDAC1/2 regulated genes	
Idasa	Idasa+Pano	Idasa	Idasa+Pano	Idasa	Idasa+Pano
-5.780762	-5.256762	-4.524822	-4.35782238	2.2007474	3.402747363
-6.825507	-8.504507	-4.579675	-5.09167545	1.9097474	3.140747363
-7.509528	-7.531528	-5.994646	-5.36864556	1.4548472	4.119847166
-6.545515	-6.794515	-5.788419	-4.4404191	-0.735555	0.81044479
-8.066708	-7.884708	-3.563349	-3.92034903	-1.1122	0.382800383
-6.073162	-5.274162	-5.577315	-5.03331507	-1.326563	0.827436838
-7.496017	-7.622017	-6.192594	-6.13459435	-0.299217	1.184783065
-6.299206	-7.041206	-3.910068	-3.28606793	1.8957474	3.148747363
-5.123271	-5.268271	-3.434781	-3.71978149	1.0308472	4.278847166
-5.397762	-5.304762	-4.693822	-4.45982238	-0.573555	0.94044479
-7.982507	-8.967507	-5.046675	-5.26667545	-1.1352	0.005800383
-6.987528	-7.447528	-5.848646	-5.35464556	-1.144563	0.790436838
-6.676515	-5.970515	-4.742419	-5.0954191	0.0787831	1.305783065
-8.241708	-7.938708	-3.722349	-3.70734903	1.3724909	2.237490852
-5.988162	-4.772162	-5.382315	-4.90331507	2.7990586	5.609058647
-7.857017	-7.988017	-5.748594	-6.16859435	-0.546072	1.671928247
-6.051206	-7.053206	-3.627068	-3.35106793	-2.399041	0.348959326
-4.997271	-5.248271	-3.614781	-3.83778149	-1.164583	1.487416737
-5.3998889	-4.8568889	-3.703781	-3.68978149	-2.064068	0.940932073
-8.03860614	-8.4846061	-5.333068	-4.80706793	1.3884909	2.149490852
-6.41313862	-6.2641386	-6.003125	-4.48512511	2.5870586	5.626058647
-6.4100968	-5.9640968	-4.664138	-4.72313813	-0.289072	1.557928247
-8.0632796	-7.4272796	-4.506173	-4.30817339	-3.319041	0.465959326
-5.83088891	-5.7538889	-4.551249	-4.52824925	-0.904583	1.419416737
-8.29800555	-7.1990055	-5.865444	-4.98144404	-1.478068	0.653932073
-6.19316774	-5.9981677	-4.333126	-3.46912573		
-4.66115283	-4.2781528	-3.068125	-3.39412511		
-4.8338889	-4.8188889	-3.636781	-3.60578149		
-7.47160614	-7.8126061	-5.169068	-4.81806793		
-6.85613862	-6.2791386	-5.681125	-5.28012511		
-6.5550968	-6.1690968	-4.972138	-5.20113813		
-7.7462796	-7.4822796	-4.328173	-4.71617339		
-7.05988891	-5.4858889	-4.834249	-4.94424925		
-7.87200555	-7.3380055	-5.683444	-5.53144404		
-6.59016774	-6.1511677	-4.100126	-3.25712573		
-4.64715283	-4.5021528	-3.055125	-3.12412511		

	Idasa				Idasa+Pano				T-Test / p values
PLK1	3.018551	3.294036	4.010706	4.0498155	2.7299153	2.768023497	5.230164	4.478005	0.769136899
BUB1	1.784916	2.327621	2.31475	3.4267862	2.56659594	2.4826075	3.372588	3.462601	0.283991344
CDC25C	1.408204	1.493665	1.71696	0.7324774	2.450126419	3.469808855	1.811087	2.180798	0.033125797
UBE2C	1.323957	1.572278	1.346166	1.0223266	0.791606953	0.78504986	1.540989	1.385932	0.434836944
NEK2	1.06574	0.973232	1.143024	1.0337277	0.896798761	1.587609598	1.557094	1.350839	0.121597494
FOXM1	0.515433	0.740133	1.157375	0.8513688	0.507632446	0.538066424	1.283298	1.270024	0.754305385
CCNB2	0.884452	0.396627	0.38498	0.570325	0.276213586	0.200386357	0.282605	0.450268	0.091335475
MKI67	0.379417	0.336075	0.345763	0.4307301	0.430431686	0.414618441	0.537321	0.517222	0.034403385
BIRC5	0.55128	0.42924	0.318608	0.4280515	0.505175364	0.391981152	0.682479	0.619792	0.189733729
BRCA1	9.068451	8.004749	12.08258	12.191943	7.442855309	6.85832461	9.638818	11.62256	0.378577628
RAD51	8.759547	7.84545	4.623086	5.230164	6.839335633	7.927446246	5.303174	3.99683	0.66656534
E2F8	6.588071	8.015854	5.165315	6.0706718	10.15315495	9.705860938	9.401273	10.88943	0.001873763
CDC6	4.254107	3.783853	7.829153	8.2013203	4.776176627	4.450156861	7.905497	8.379454	0.823539955
ZNF367	2.026312	2.319568	4.407181	3.6221748	2.954381615	3.232961192	4.478005	3.356264	0.550675959
MCM5	4.11775	2.979058	2.456929	2.7527168	2.887570539	2.557716192	3.537809	3.510937	0.917831683
E2F1	1.732501	3.577263	3.682936	2.974931	4.410237011	2.800833436	3.535358	2.538289	0.610989782
MYBL2	1.383053	1.881465	1.620969	1.8389168	1.439787791	1.406253015	2.991473	2.043237	0.484228305
ORC1	1.50823	1.668852	1.538854	1.9236631	2.327621036	2.350318362	4.407181	2.540049	0.050655382
CTGF	270.1948	201.3911	666.2206	575.17461	1713.649317	1913.311675	4672.102	4727.482	0.015243556
ARRDC4	368.8412	365.2793	271.3209	274.34666	865.7797156	870.5939533	494.1675	464.9256	0.0221824
VGF	62.9815	70.46604	95.92641	114.63122	183.9100092	201.2515647	446.2957	412.3873	0.018074324
RGL1	86.03526	111.8062	23.68425	35.551935	240.6606052	261.7156143	190.1318	155.8329	0.003507459
MTX1	39.50207	44.81333	44.13515	52.850902	175.8079122	171.356391	277.4062	264.6343	0.000789835
DHRS2	47.04131	46.2973	19.49262	10.302026	132.592576	102.1012126	130.9485	142.0107	0.000279631

P value	0.3926	P value	0.1185	P value	0.0005	Ratio Paired T-Test
P value summary	ns	P value sun	ns	P value sum	***	

Supplementary Fig. 3c

Idasa	Idasa+Pano	Idasa	Idasa+Pano	Idasa	Idasa+Pano
-5.4380008	-6.0485008	-4.744353	-4.03085338	1.7197565	3.26725653
-7.8171113	-8.3246113	-3.495791	-3.52229145	0.361603	1.963102995
-6.7625003	-7.6310003	-5.111578	-3.87807798	0.6595023	2.906002348
-6.973517	-5.331017	-4.833855	-4.24735486	0.8707318	1.711231826
-8.9522332	-7.4637332	-3.398955	-3.31645503	2.0389327	3.55743273
-7.6250827	-5.6665827	-4.428875	-3.83537511	3.2258086	4.341308613
-7.2545055	-7.6310055	-6.412512	-6.04301248	1.6247565	3.31125653
-7.9901061	-8.3956061	-4.606353	-4.02885338	0.402603	1.856102995
-5.4990008	-6.1685008	-3.574791	-3.48729145	0.6885023	3.557002348
-7.5361113	-8.2136113	-4.714578	-3.83107798	0.8867318	1.606231826
-6.9525003	-7.1800003	-5.788855	-4.06835486	2.0569327	3.51843273
-6.257517	-5.555017	-3.298955	-3.30245503	3.2168086	4.399308613
-8.6902332	-7.0777332	-4.419875	-3.87937511	1.7739349	3.902434926
-6.6690827	-5.2545827	-6.312512	-5.88101248	-0.445818	1.640681607
-6.8015055	-7.4020055	-4.975616	-4.68511604	1.1041115	2.430611472
-8.1561061	-8.3616061	-2.86875	-4.08525	-0.193008	1.010491543
-7.07226949	-8.08076949	-5.629616	-4.60911604	2.0628785	3.722378532
-6.77329583	-6.71879583	-4.199225	-4.81572466	1.4775643	3.195084263
-4.66047535	-5.54397535	-3.902385	-4.72088462	1.8729349	3.983434926
-7.4678468	-8.3933468	-6.132518	-6.31101817	-0.412818	1.597681607
-6.33971167	-7.66421167	-4.875616	-4.78211604	1.2461115	2.056611472
-7.42426949	-8.04776949	-3.63175	-3.99025	0.0989915	1.346491543
-6.73929583	-6.99779583	-5.091616	-4.63111604	2.0098785	3.848378532
-4.65547535	-5.19797535	-5.426225	-4.15672466	1.4665643	3.121064263
-7.2258468	-7.7293468	-3.628385	-4.99488462		
-6.80071167	-7.43821167	-5.731518	-6.16701817		

	Idasa				Idasa+Pano				T-Test / p values
CDC25C	0.465662	0.90335	4.025328	4.039302493	1.80983251	2.408034714	2.181932	2.773305	0.949749217
BUB1	2.309141	2.213541			1.51241723	1.391707657			0.008929557
NEK2	0.799603	1.313445			2.496412239	2.137398912			0.056634433
FOXM1	0.920415	0.806841	0.921532	0.943507695	0.504125973	0.689133879	0.95701	0.788732	0.152125887
UBE2C	0.409336	0.364845	1.204226	0.874849156	0.309038008	0.316407607	0.480832	0.562376	0.207659671
BIRC5	0.656723	0.898977	0.581603	0.687821534	0.505876171	0.592898146	0.306213	0.485185	0.042066255
CCNB2	0.430879	0.523534	0.746962	0.585242396	0.30309799	0.327338724	0.371287	0.379878	0.016777995
MKI67	0.208534	0.250062			0.58514099	0.764641914			0.040182012
MCM5	8.69	8.226942	13.69053	8.06741342	8.531835737	8.741350517	5.891382	6.292382	0.184481593
RAD51	9.83794	10.54404	7.071941	8.55107731	10.41691509	10.51849353	4.010011	3.316373	0.394820215
ZNF367	4.448615	4.476453			6.712527948	6.510896431			0.002234884
CDC6	3.899681	4.291126	3.108258	3.331348279	6.394609186	6.403480144	3.801599	3.5544	0.147954229
E2F1	3.585952	1.849783	5.744201	2.453950571	5.384663498	6.095971635	3.746663	5.915935	0.112639902
ORC1	2.86266	3.769456	2.066381	3.000298808	6.731164851	6.954063254	4.191905	4.128467	0.023119033
MYBL2	1.169066	1.252974	1.368036	1.806385975	1.510322027	1.689804318	1.208826	1.335709	0.840448694
DHRS2	920.5525	914.8277	290.2407	288.0361722	1994.561911	2076.382027	954.5032	906.7784	0.053739059
RGL1	427.3743	432.7399	440.2277	424.3486242	1224.397416	1191.74197	1390.71	1517.632	2.19207E-05
ARRDC4	310.2648	290.4923	330.8667	354.3684097	906.9355302	935.0217988	1446.756	1530.308	0.001760681
VGF	147.6826	150.6813	189.5069	209.1082526	700.7986521	1100.433455	475.2709	366.7381	0.024424322
MTX1	175.5035	177.4608	93.77336	114.8101347	314.2690067	292.2088757	215.9579	272.5933	0.004309432
CTGF	122.2217	125.7449	71.41257	73.06487901	370.8922186	344.3796753	303.3009	294.3944	6.26363E-05

P value	0.8915	P value	0.2133	P value	0.0002	Ratio Paired T-Test
P value summary	ns	P value sun	ns	P value sum	***	

Supplementary Fig. 3e

	WT Palbo				WT Palbo+Romi				WT Palbo+Pano				SIN3B$^{-/-}$ Palbo				SIN3B$^{-/-}$ Palbo+Romi				SIN3B$^{-/-}$ Palbo+Pano			
BUB1	-3.194914	-3.2209143	-2.778414	-2.8251325	-4.549414	-4.453414	-3.541914	-3.573559	-3.844414	-3.987414	-4.530914	-4.3609493	-2.8463736	-2.8053736	-2.867874	-2.808874	-4.1788736	-3.9228736	-3.1818736	-3.1248736	-4.600374	-4.719374	-3.6158736	-3.5428736
ORC1	-3.405625	-3.5886247	-2.871125	-2.8914791	-4.629125	-4.835125	-3.749625	-3.829296	-3.564125	-3.472125	-4.878625	-4.9038994	-2.7800415	-3.1330415	-3.172542	-3.008542	-4.7835415	-4.7865415	-3.1225415	-3.1255415	-4.887042	-5.514042	-4.2285415	-4.3305415
CTGF	0.2072	0.17920036	0.7047004	0.72483898	3.8557004	3.7907	3.1822	3.557593	2.4227	2.3857	1.3072	1.78941688	-0.3944146	-0.3854146	-0.166915	-0.082915	5.65208543	4.77008543	3.60808543	4.40008543	2.200585	2.180585	1.54408543	1.44408543

	LIN37$^{-/-}$ Palbo				LIN37$^{-/-}$ Palbo+Romi				LIN37$^{-/-}$ Palbo+Pano				SIN3B$^{-/-}$;LIN37$^{-/-}$ Palbo				SIN3B$^{-/-}$;LIN37$^{-/-}$ Palbo+Romi				SIN3B$^{-/-}$;LIN37$^{-/-}$ Palbo+Pano			
BUB1	-1.340191	-1.295191	-1.564691	-1.674691	-1.389191	-1.253191	-1.783691	-1.723691	-0.997191	-1.109191	-1.759691	-1.822691	-1.121799	-1.192799	-0.672299	-0.795299	-0.919799	-0.858799	-1.693799	-1.664799	-0.920299	-0.946299	-1.682799	-1.822799
ORC1	-2.412587	-2.335587	-2.749087	-2.828087	-2.463587	-2.619587	-2.542087	-2.589087	-2.085587	-1.872587	-2.740087	-2.754087	-1.477502	-1.603502	-1.630002	-1.788002	-1.238502	-1.247502	-2.311502	-2.365502	-1.527002	-1.641002	-2.408502	-2.399502
CTGF	-0.197545	-0.268545	-0.602045	-0.696045	4.867455	4.858455	5.435955	5.330955	1.822455	1.868455	2.582955	2.349955	-0.812038	-0.854038	-1.326538	-1.275538	5.343962	5.322962	3.530962	3.234962	2.557462	2.595462	1.704962	1.628962

		WT Palbo+Romi		WT Palbo+Pano		SIN3B$^{-/-}$ Palbo+Romi		SIN3B$^{-/-}$ Palbo+Pano		LIN37$^{-/-}$ Palbo+Romi		LIN37$^{-/-}$ Palbo+Pano		SIN3B$^{-/-}$;LIN37$^{-/-}$ Palbo+Romi		SIN3B$^{-/-}$;LIN37$^{-/-}$ Palbo+Pano	
BUB1		0.007719	**	0.001102	**	0.012155206	*	0.000862	***	0.71325	ns	0.727159	ns	0.263105	ns	0.201535	ns
ORC1	T-Test	0.015376	*	0.046963	*	0.090933643	ns	0.000227	***	0.917084	ns	0.377782	ns	0.827888	ns	0.201542	ns
CTGF		0.000113	***	0.008754	**	0.016323003	*	0.001364	**	8.49E-05	***	0.000674	***	0.027712	*	0.002309	**
